# Nitrogen Reduction to Ammonia on a Biomimetic Mononuclear Iron Centre: Insights into the Nitrogenase Enzyme

**DOI:** 10.1002/chem.201704688

**Published:** 2017-12-14

**Authors:** Monika A. Kaczmarek, Abheek Malhotra, G. Alex Balan, Amy Timmins, Sam P. de Visser

**Affiliations:** ^1^ Manchester Institute of Biotechnology and School of Chemical, Engineering and Analytical Science The University of Manchester 131 Princess Street Manchester M1 7DN UK; ^2^ Department of Chemistry University of Warsaw Ludwika Pasteura 1 02-093 Warsaw Poland

**Keywords:** biomimetic synthesis, density functional theory, enzyme models, iron, nitrogen

## Abstract

Nitrogenases catalyse nitrogen fixation to ammonia on a multinuclear Fe‐Mo centre, but their mechanism and particularly the order of proton and electron transfer processes that happen during the catalytic cycle is still unresolved. Recently, a unique biomimetic mononuclear iron model was developed using tris(phosphine)borate (TPB) ligands that was shown to convert N_2_ into NH_3_. Herein, we present a computational study on the [(TPB)FeN_2_]^−^ complex and describe its conversion into ammonia through the addition of electrons and protons. In particular, we tested the consecutive proton transfer on only the distal nitrogen atom or alternated protonation of the distal/proximal nitrogen. It is found that the lowest energy pathway is consecutive addition of three protons to the same site, which forms ammonia and an iron‐nitrido complex. In addition, the proton transfer step of complexes with the metal in various oxidation and spin states were tested and show that the p*K*_a_ values of biomimetic mononuclear nitrogenase intermediates vary little with iron oxidation states. As such, the model gives several possible NH_3_ formation pathways depending on the order of electron/proton transfer, and all should be physically accessible in the natural system. These results may have implications for enzymatic nitrogenases and give insight into the catalytic properties of mononuclear iron centres.

## Introduction

Nitrogenases are vital enzymes for life on Planet Earth and catalyse the conversion of N_2_ from the atmosphere into ammonia: one of the building blocks of biological molecules.^1^ The active site of nitrogenase is still controversial, but it is now accepted to contain an iron‐molybdenum cluster (FeMoco) with seven iron atoms and one terminal molybdenum atom with all metal atoms bridged by nine sulfur atoms. The inner atom of the FeMoco cluster has long remained uncharacterised but recent experimental evidence has implicated it to be a carbon atom, although its function remains unclear.^2^ Nitrogenase catalyses the overall reduction of N_2_ to two NH_3_ molecules by using eight protons and eight electrons that are delivered through a proton‐transfer channel and electron‐transfer machineries, respectively.^3^ The exact position of the nitrogen binding to the FeMoco cluster is unknown and its catalytic mechanism remains a mystery; therefore, extensive spectroscopic, kinetics and electrochemical studies have been performed to obtain insights into the intricate details of the mechanism.^4^ Detailed computational studies tested possible mechanisms as well as the coordination environment and the function of the central carbon of the FeMoco cluster.^5^, ^6^ However, as the reaction process on the FeMoco cluster is fast, there remain many controversies related to the mechanism and particularly to the order of proton and electron transfer. As a result, model complexes have been developed to gain insight into these chemical processes.

**Scheme 1:**
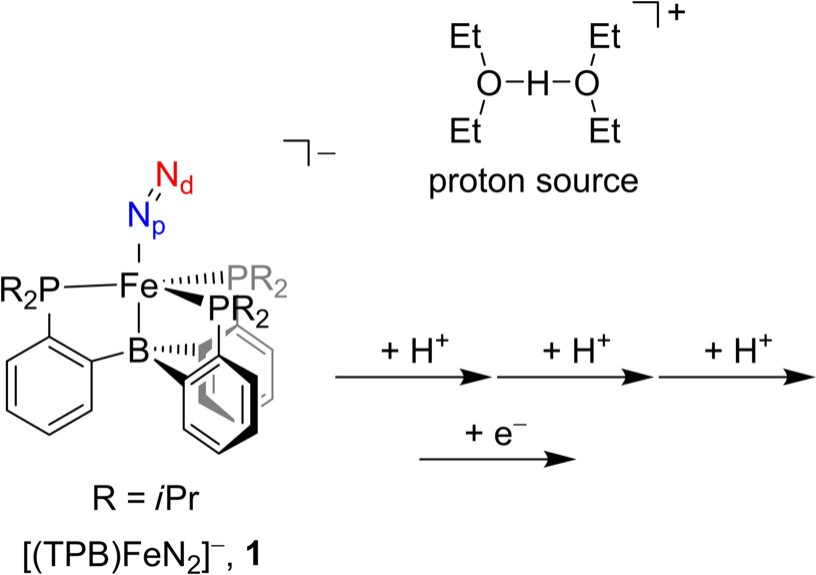
Complex and reaction mechanism investigated in this work.

Thus, to understand the structural and functional features of enzyme active sites, biomimetic models are often developed that mimic the reactive centre; that is, the metal with its ligand coordination features, but these are then studied in solution.^7^ Specific biomimetic models of nitrogenase have been designed and developed to gain insight into the nitrogen fixation process and the features of the reaction complex needed for efficient turnover.^8^ In particular, studies focused on the synthesis and characterisation of mononuclear iron and manganese systems with nitride and/or imido bound.^9^, ^10^ In one such biomimetic model complex, the Peters group used a mononuclear iron system and found evidence of nitrogen reduction to ammonia.^11^ As such, this would be one of the few synthetic mononuclear iron complexes known to be able to reduce nitrogen to ammonia through the addition of external protons and electrons in analogy to the FeMoco cluster of nitrogenase. Scheme 1 displays the mononuclear iron centre with tris(phosphine)borane (TPB) and N_2_ bound, [(TPB)FeN_2_]^−^, **1**. By using electron paramagnetic resonance (EPR), Mössbauer and extended X‐ray absorption fine structure (EXAFS) spectroscopy, several nitrogen‐based intermediates were trapped and characterised including a doubly protonated hydrazido complex.11a The authors studied the proton transfer reactions with (Et_2_O)_2_H^+^ as an acid in tetrahydrofuran solvent.

**Scheme 2:**
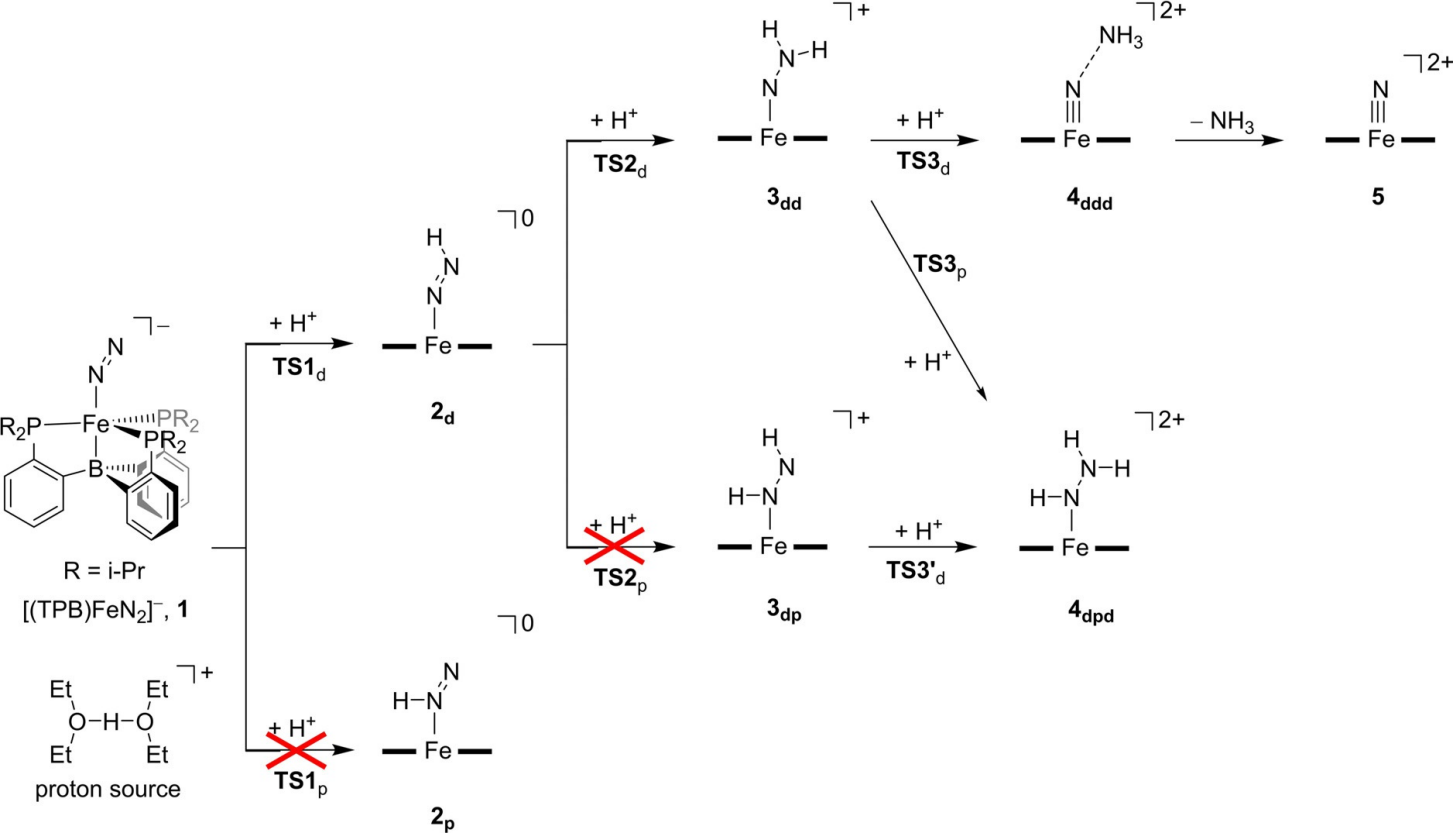
Reaction mechanism for the protonation steps of [(TPB)FeN_2_]^−^ in the absence of reduction partner with nomenclature of structures involved.

Currently, little is known on the proton‐ and electron‐transfer processes in nitrogenases and particularly lacking is the order in which these processes happen. The same is true for the synthetic mononuclear model shown in Scheme 1. Technically, the first three proton transfers can take place consecutively on the terminal nitrogen atom of N_2_ (the distal N atom, N_d_), so that it can split off an ammonia molecule and leave an iron‐nitrido complex. Scheme 2 describes the possible reaction mechanisms that were studied in this work starting from the reactant complex **1**. The first step considers single proton transfer to either the distal nitrogen (via **TS1**_d_) or the proximal nitrogen atom (via **TS1**_p_) to form the singly‐protonated species **2**_d_ and **2**_p_, respectively. The next proton transfer onto **2**_d_ gives an [FeNHNH] (**3**_dp_) or [FeNNH_2_] (**3**_dd_) complex via transition states **TS2**_p_ or **TS2**_d_. Proton transfer towards **3**_dp_ and **3**_dd_ can give the ammonia complex **4**_ddd_ or the [FeNHNH_2_] complex **4**_dpd_ and their mechanistic pathways were studied here as well. Thus, we consider consecutive proton transfer as well as an alternating proton transfer, in which the first proton is donated to the distal nitrogen atom, the second proton to the proximal nitrogen atom (N_p_), the third proton to the distal nitrogen atom again, etc.

In addition, each proton‐transfer step can be followed by electron transfer directly. Clearly, the mechanism of nitrogen reduction to ammonia on a nonheme iron centre can take place through many possible reaction pathways. To gain insight into the mechanism of ammonia production from N_2_ on a mononuclear iron centre, we performed a computational study, which may have further relevance to the mechanism of nitrogenase enzymes. Our comprehensive DFT study on the proton‐ and electron‐transfer mechanisms for reduction of N_2_ to ammonia considers a large variety of possible processes ranging from consecutive proton transfer to alternating proton/electron transfer steps. These studies give valuable insight into the nitrogen fixation reaction using the mononuclear iron model from Scheme 1. We show that, technically, a triple proton transfer without electron donation can lead to the release of ammonia; however, lower energy pathways are found when one or more electrons are donated into the chemical system. As such, it appears that the catalytic centre will not necessarily need an electron after each proton abstraction, but if it happens the overall nitrogen fixation process will be more efficient.

## Results and Discussion

The study presented in this work started from the [(TPB)FeN_2_]^−^ complex shown in Scheme 1 and Scheme 2. At the UB3LYP/BS2//UB3LYP/BS1 + ZPE (zero‐point energy) level of theory, the doublet spin state is the ground state, whereas the quartet and sextet spin states are higher lying by 4.3 and 23.9 kcal mol^−1^, respectively. Geometrically, the doublet and quartet spin states have a linear Fe‐N‐N group, whereas it is bent (by 137°) in the sextet spin state. Although we calculated the lowest three spin states for each structure, we will focus on the lowest lying one in the main paper only; the results covering all other spin states are deposited in the Supporting Information.

### Consecutive protonation pattern

We first considered the mechanism of three consecutive proton‐transfer reactions starting from the iron‐dinitrogen complex **1** in the absence of a reduction partner and with (Et_2_O)_2_H^+^ as the proton source, see Scheme 2. In particular, we calculated the [(TPB)FeN_p_(H)_*x*_N_d_(H)_*y*_]^−1+*x*+*y*^ complexes with *x=*0–3 and *y=*0–3 and assume that only proton transfer reactions take place. We considered complexes with the distal as well as proximal nitrogen atom protonated. However, the structures with a value of *x*>*y*, that is, more protons bound to the proximal nitrogen than to the distal nitrogen atom, either failed to converge or, during the geometry optimisation, were reoriented in such a way that the nitrogen atom with the most number of protons became the terminal nitrogen atom (N_d_). Therefore, the [(TPB)FeN_p_(H)N_d_]^0^ structure (**2**_p_) is unstable and its geometry optimisation gave [(TBP)FeNNH]^0^ (**2**_d_) instead. Consequently, the first proton transfer can only take place on the distal nitrogen atom. Structure **2**_d_ has close lying doublet and quartet spin state structures (within 1 kcal mol^−1^) and hence will react through two‐state reactivity on competing doublet and quartet spin surfaces, similar to what was seen for mononuclear iron(IV)‐oxo complexes with heme and nonheme ligand environments.^12^, ^13^ In the next step of the catalytic cycle, we attempted to protonate **2**_d_ and located two local minima, namely [(TPB)FeN_p_(H)N_d_(H)]^+^ (**3**_dp_) and [(TPB)FeN_p_N_d_H_2_]^+^ (**3**_dd_), whereby the latter is 2.9 kcal mol^−1^ lower in energy than the former in the quartet spin state.

We then calculated the driving force for proton transfer from (Et_2_O)_2_H^+^ from ^2^ **2**_d_ leading to these structures using isolated species according to reactions 1 and 2 and find values of Δ*E*+ZPE=−14.6 kcal mol^−1^ for Equation (1) and −12.5 kcal mol^−1^ for Equation (2). Based on these driving forces, therefore, terminal protonation of the diazenide group will be preferred over proximal protonation, but only by a few kcal mol^−1^. Consequently, the kinetics for both pathways was considered here.

(1) $${}^{2}\left[\left(\mathrm{TPB}\right)\mathrm{FeNNH}\right]{}^{0}+\left(\mathrm{Et}{}_{2}O\right){}_{2}H{}^{+}\to {}^{2}\left[\mathrm{FeNNH}{}_{2}\right]{}^{+}+2\;\mathrm{Et}{}_{2}O$$

(2) $${}^{2}\left[\left(\mathrm{TPB}\right)\mathrm{FeNNH}\right]{}^{0}+\left(\mathrm{Et}{}_{2}O\right){}_{2}H{}^{+}\to {}^{2}\left[\mathrm{FeN}\left(H\right)\mathrm{NH}\right]{}^{+}+2\;\mathrm{Et}{}_{2}O$$

In the final proton‐transfer step in Scheme 2 (top), we investigated addition of a third proton to the distal nitrogen atom, which forms ammonia and immediately breaks the N−N bond to form **4**_ddd_. The ground state of **4**_ddd_ is the quartet spin state in analogy to iron(IV)‐tosylimido complexes calculated previously.^14^ Higher in energy than the quartet spin ground state are the sextet spin state (by 4.5 kcal mol^−1^) and the doublet spin state by 30.7 kcal mol^−1^. The final dissociation of ammonia from the ^4^ **4**_ddd_ complex only costs 5.4 kcal mol^−1^ at the Δ*E*+ZPE level of theory through the breaking of the hydrogen bond between ammonia and the iron(IV)‐nitrido group. We also tested the proximal protonation of **3**_dd_ (as will be discussed in the next section) and found it to be similar in energy.

Subsequently, we investigated the kinetics of the proton‐transfer reactions displayed in Scheme 2. Accordingly, we created models of structures, **1**, **2**_d_, **3**_dd_, **3**_dp_ and **4**_ddd_, and included a proton source as used in Ref. 11a, that is, (Et_2_O)_2_H^+^, to the complex. The structures with (Et_2_O)_2_H^+^ included in the model are labelled with RC in subscript after the name and have the same spin state ordering and relative energies as the isolated structures. Thus, **1**_RC_ represents [**1**‐(Et_2_O)_2_H^+^], **2**_RC_ is [**2**‐(Et_2_O)_2_H^+^]^+^ etc.

**Figure 1:**
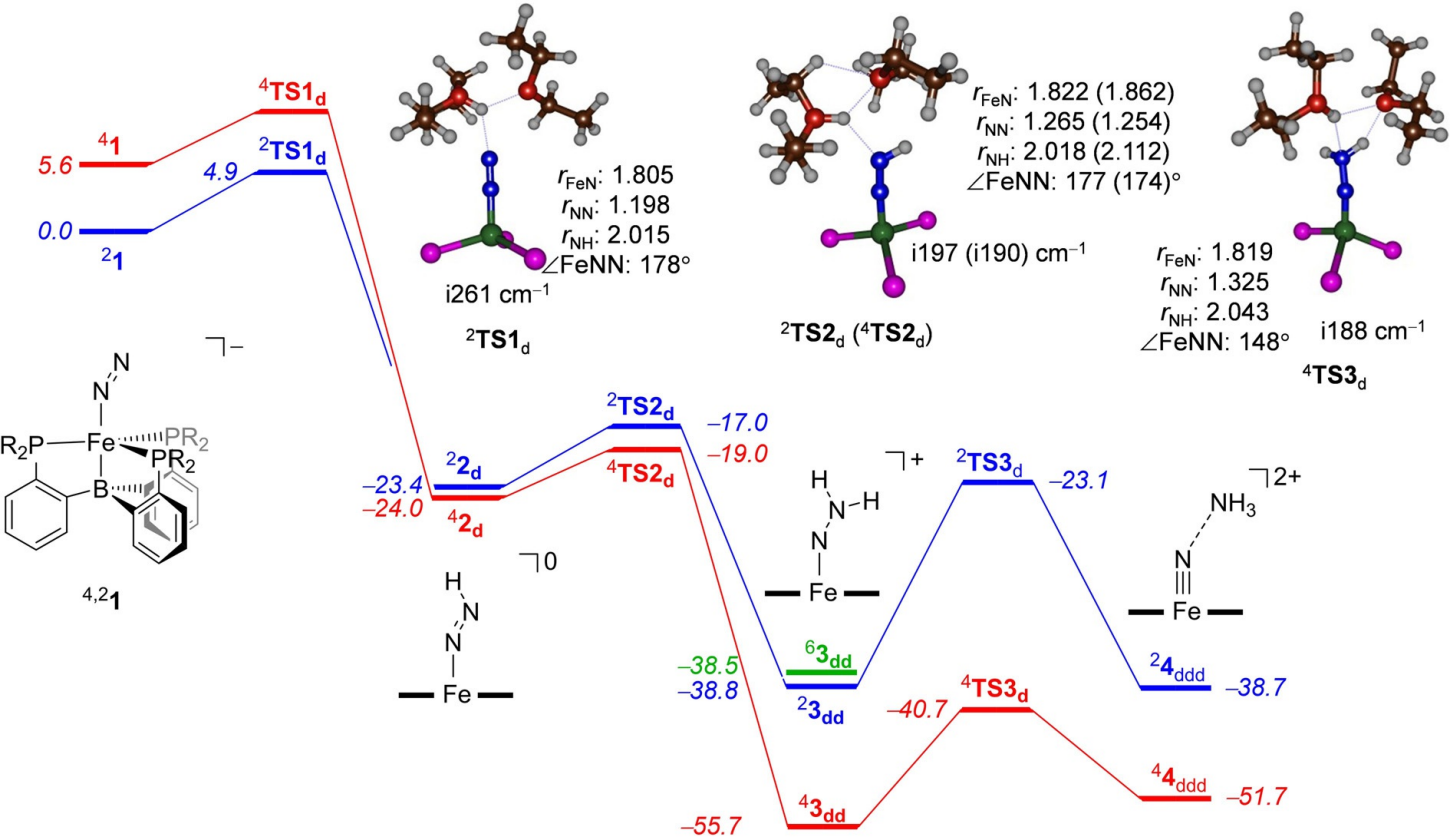
Potential energy landscape (with energies in kcal mol^−1^) for consecutive protonation of the distal nitrogen atom in complex ^4,2^ **1** as calculated at the UB3LYP/BS2//UB3LYP/BS1 level of theory with solvent and zero‐point corrections included. Extracts of the optimised geometries of the transition states give bond lengths in angstroms, angles in degrees and the imaginary frequency in cm^−1^.

Figure 1 gives the energy landscape and structures of low‐energy proton‐transfer transition states for the consecutive proton transfer to **1** in the absence of an electron donor. For each of the individual reactant complex structures, that is, **1**_RC_, **2**_d,RC_, **3**_dd,RC_, **3**_dp,RC_ and **4**_ddd,RC_, on the doublet, quartet and sextet spin states a geometry scan for proton transfer was performed, whereby one degree of freedom (the reaction coordinate) was fixed and all other geometric degrees of freedom minimised. The maxima of these geometry scans were subjected to a transition state search and gave us the proton‐transfer transition states for each of the reaction steps. Thus, ^2,4,6^ **TS1**_d_ are the transition states for the proton transfer from (Et_2_O)_2_H^+^ to ^2,4,6^ **1** at the distal nitrogen atom.

The first pathway investigated relates to the proton transfer from (Et_2_O)_2_H^+^ to [(TPB)FeN_2_]^−^ to give the [(TPB)FeNNH] and two diethyl ether molecules. Optimised geometries starting from the end point of the geometry scans originating from ^2,4,6^ **1**_RC_ resemble the ^2,4,6^ **2**_d_ optimised structures (see Supporting Information, Figure S3) and confirm the pathway from **1** to **2**_d_. Subsequently, we took the ^2,4,6^ **2**_d_ structures and added the proton donor group (Et_2_O)_2_H^+^ and optimised ^2,4,6^ **2**_d,RC_. Thereafter, the proton‐transfer pathway was investigated and led to the transition states for distal protonation. The energy landscape described in Figure 1 is, therefore, based on the relative energies of the reactant complexes and the transition states with respect to ^2^ **1**. The first proton transfer to [(TPB)FeN_2_]^−^ takes place on the doublet spin state via ^2^ **TS1**_d_ and has an energy of 4.9 kcal mol^−1^, which is lower in energy than the nearest quartet and sextet spin states. By contrast, optimised geometries of structures **2** implicate them to be close in energy on the doublet and quartet spin state surfaces. Consequently, during the lifetime of structure **2** it is likely that full equilibration will lead to a mixture of doublet and quartet spin state structures. Geometrically, ^2^ **1** and ^2^ **2**_d_ structures contain very similar Fe‐N and N‐N distances and hence their electronic configuration is the same.

The optimised geometry of ^2^ **TS1**_d_ is given in Figure 1 and is characterised with a small imaginary frequency of i261 cm^−1^. This value is much lower than those typically found for hydrogen atom abstraction barriers of well over i1500 cm^−1^ due to a sharp and narrow peak on the potential energy surface.^15^ As such, the proton transfer potential energy surface will be flatter with a broad barrier. Moreover, transition states with large imaginary frequencies generally give rate constants with a large kinetic isotope effect, which is not expected in our system described here. Thus, replacement of the transferring proton in (Et_2_O)_2_H^+^ by a deuteron may increase the barriers slightly but not by a huge amount.^16^ The donating proton in ^2^ **TS1**_d_ is located at a relatively large distance of 2.02 Å, which appears unusually long. However, as can be seen from the structure, ^2^ **TS1**_d_ reflects the simultaneous breaking of the Et_2_OH^+^⋅⋅⋅OEt_2_ hydrogen bond and the transfer of the proton to N_d_. These two simultaneous steps prevent a closer approach of the proton to N_d_.

The reaction step from ^2,4,6^ **2**_d_ can again lead to distal and proximal protonation to form the isomeric complexes ^2,4,6^ **3**_dd_ and ^2,4,6^ **3**_dp_, respectively. However, despite the fact that the geometries of ^2,4,6^ **3**_dp_ could be optimised, no valid reaction pathways were identified for direct proton transfer from (Et_2_O)_2_H^+^ to the proximal position of ^2,4,6^ **2**_d_. In particular, all geometry scans for proximal protonation of ^2,4,6^ **2**_d_ failed, and led to distal protonation transition states instead. Clearly, distal protonation of **2** is energetically and kinetically favoured over proximal protonation.

Distal protonation of ^2,4^ **2**_d_ leads to ^2,4^ **3**_dd_ via a barrier ^2,4^ **TS2**_d_ with values of 6.4 (doublet) and 5.0 (quartet) kcal mol^−1^ with respect to their precursor local minima. These structures, similar to ^2^ **TS1**_d_, have a small imaginary frequency of i197 (i190) cm^−1^ for ^2^ **TS2**_d_ (^4^ **TS2**_d_), respectively. Also in these structures the transferring proton is still located at a large distance; hence, the structures are early on the potential energy surface. Previously,^17^ early transition states were correlated with barrier heights with low energy, in agreement to what is seen in Figure 1.

Formation of intermediate **3**_dd_ from **2**_d_ leads to significant changes in bond lengths along the Fe−N and N−N interactions. To be specific, the Fe−N bond shortens to 1.718 Å in ^2^ **3**_dd_ as a result of increasing double bond character in the Fe−N interaction. At the same time, the N−N bond elongates to well over 1.32 Å and loses double bond character. We find the quartet spin state to be the ground state of **3**_dd_ and **3**_dp_ with the doublet and sextet spin states well above the quartet spin state by 17.2 and 14.7 kcal mol^−1^, respectively. These results appear to contradict experimental observations that implicated a doublet spin ground state.11a However, the doubly reduced complex (**3**_dd_^−^), which we also calculated has a doublet spin ground state. It could very well be, therefore, that the experimentally characterised EPR spectrum may reflect the doubly reduced species instead. Further work will be necessary to establish this.

The lowest lying transition state for protonation of **3**_dd_ is on the quartet spin state surface with a barrier (^4^ **TS3**_d_) of about 15.0 kcal mol^−1^. Therefore, the energetic barrier for the third consecutive protonation step is considerably larger than the first two proton transfer barriers. Furthermore, the reaction from ^4^ **3**_dd_ leading to ^4^ **4**_ddd_ is endothermic by 4.0 kcal mol^−1^. Structurally, it is akin to ^4^ **3**_dd_ with Fe‐N and N‐N distances of 1.819 and 1.325 Å. Similar to the ^2^ **TS1**_d_ and ^2, 4^ **TS2**_d_ structures discussed above, the donating proton is at a relatively large distance from N_d_ of well over 2 Å and has a small imaginary frequency of i148 cm^−1^. After passing the proton transfer transition states ^2,4,6^ **TS3**_d_, the geometry scans fall to complex **4**_ddd_, which is an iron‐nitrido with a weakly bound ammonia molecule. This complex dissociates ammonia to form the final products.

In summary, the calculations shown in Figure 1 implicate easy and fast triple protonation of the distal nitrogen atom from [(TPB)FeN_2_]^−^ in a highly exothermic reaction pathway. Therefore, triple protonation without electron donation is a feasible process for this mononuclear nonheme iron complex. It may very well be, therefore, that in the multinuclear FeMoco cluster, binding of N_2_ can also be followed by triple protonation without electron transfer. However, reduction steps could speed up the process and hence we investigated that possibility.

### Alternating proton transfer pattern

As noted above, we failed to optimise ^2,4,6^ **2**_p_; however, we did succeed in optimising the structures of ^2,4,6^ **3**_dp_ and ^2,4,6^ **4**_dpd_ (see the Supporting Information, Figures S10 and S17). We therefore decided to investigate the proton transfer pathways leading to these intermediates. Unfortunately, in analogy to the problem associated with locating ^2^ **TS1**_p_, we also failed the geometry optimisations of ^2,4,6^ **TS2**_p_, where the proton during the geometry optimisations moved to the distal nitrogen atom instead. These transition states, therefore, must be considerably higher in energy than those leading to ^2,4,6^ **TS2**_d_.

**Figure 2:**
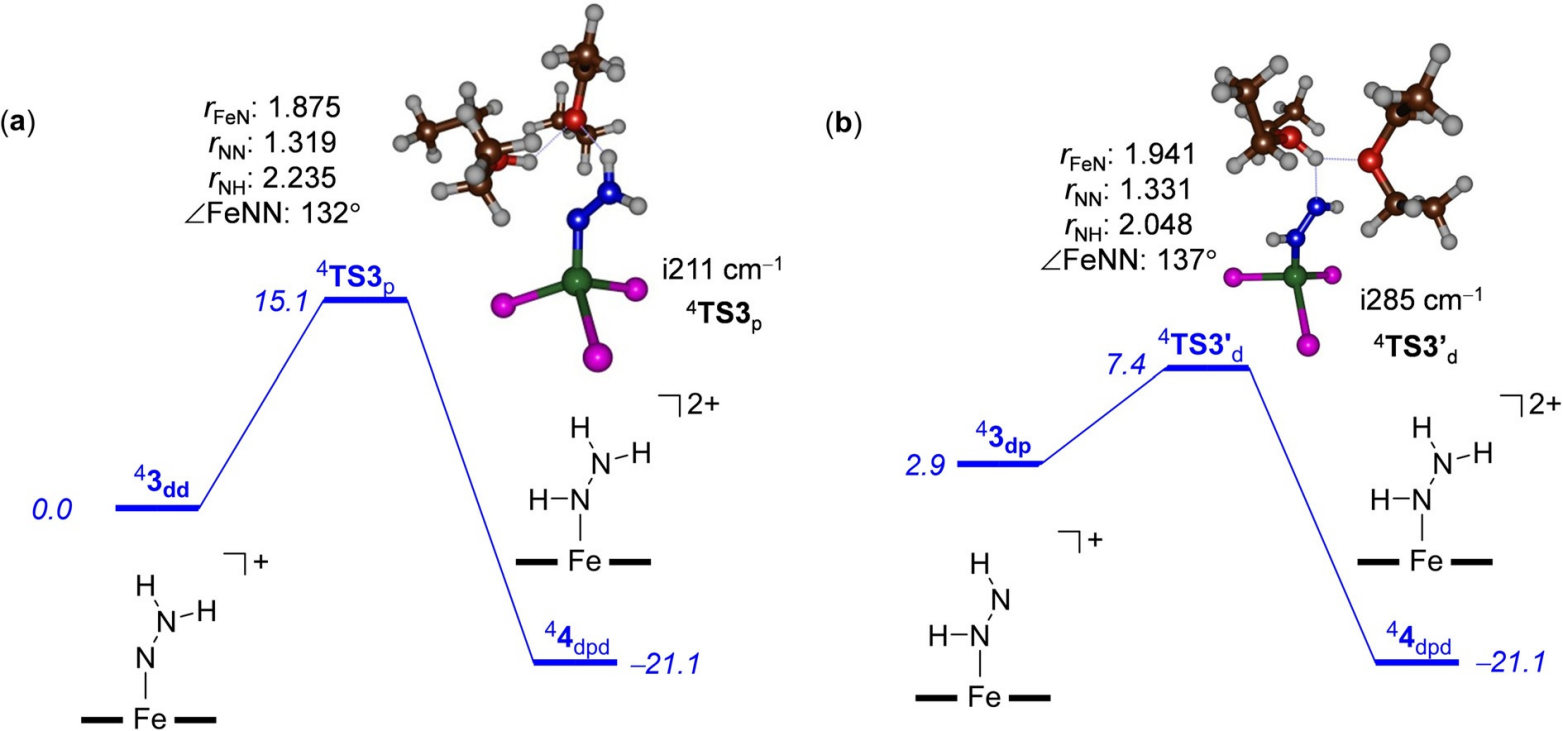
Potential energy landscape (with energies in kcal mol^−1^) for alternating protonation pathways for a) proximal protonation from ^4^ **3**_dd_. b) Distal protonation from ^4^ **3**_dp_. Calculations done at UB3LYP/BS2//UB3LYP/BS1 level of theory with solvent and zero‐point corrections included. Extracts of the optimised geometries of the transition states give bond lengths in angstroms, angles in degrees and the imaginary frequency in cm^−1^.

Subsequently, we located several transition states (^2,4,6^ **TS3′**_d_) for the distal protonation of ^2,4,6^ **3**_dp_ as well as those for proximal protonation from ^2,4,6^ **3**_dd_ (see Figure 2). Thus, starting from ^4^ **3**_dp_, a proton‐transfer transition state ^4^ **TS3′**_d_ was located for distal protonation to give [(TPB)FeN(H)NH_2_]^2+^ (^4^ **4**_dpd_) products. Structure **3**_dp_, in analogy to **3**_dd_, has a quartet spin ground state that is separated from the sextet and doublet spin states by 10.8 and 23.1 kcal mol^−1^, respectively. The proton‐transfer barrier (via ^4^ **TS3′**_d_) is about 4.5 kcal mol^−1^ in energy (Δ*E*+ZPE) above the value of ^4^ **3**_dp_ and its structure has long Fe‐N and N‐N distances of 1.941 and 1.331 Å, respectively. Similar to the transition states reported above, the imaginary frequency is small (i285 cm^−1^). Therefore, once the [(TPB)FeN(H)NH]^+^ structure is formed, it can rapidly pick up a proton to form the [(TPB)FeN(H)NH_2_]^2+^ structure along an alternative pathway.

The final alternative pathway considered was proximal protonation from [(TPB)FeNNH_2_]^+^ (**3**_dd_) (Figure 2 a). A barrier height (via ^4^ **TS3**_p_) of only 15.1 kcal mol^−1^ was found for this process, which is only 0.1 kcal mol^−1^ higher in energy than distal protonation. Consequently, ^4^ **3**_dd_ can react through either distal or proximal nitrogen protonation with almost equal barriers and hence probabilities. Structurally, the transition states for proximal and distal protonation of ^4^ **3**_dd_ are very similar.

### Proton transfer pathways of reduced complexes

In the nitrogenase enzyme as well as in biomimetic model complexes, the nitrogen reduction to ammonia is accomplished with multiple electron‐transfer steps; however, the order of the proton‐ and electron‐transfer steps remains unclear. In particular, it is very well possible that the enzyme is designed in such a way that the proton‐ and electron‐transfer steps are alternated. The above results on the mononuclear iron complex **1** show an energetically feasible pathway for ammonia synthesis without reduction steps. To test whether the proton transfer ability is enhanced by reduction of the metal centre, we calculated the proton transfer driving forces of some of the reduced complexes and, particularly, the protonation of ^2,4,6^ **3**_dd_^+^ and its one‐electron and two‐electron reduced forms, designated ^1,3,5^ **3**_dd_^0^ and ^2,4,6^ **3**_dd_^−^, where we now give the overall charge of the complex in superscript after the label. In addition, we investigated the distal protonation from ^2,4,6^ **3**_dp_^+^ and its one‐ and two‐electron reduced forms; that is, ^1,3,5^ **3**_dp_^0^ and ^2,4,6^ **3**_dp_^−^. Table 1 summarises the obtained results for the proton transfer from **3**_dd_ and **3**_dp_ with overall charge *Q*=1, 0 or −1.

**Table 1:** Proton transfer barriers and reaction energies for systems with different overall charge.^[a,b]^

Compound	*Q*=1^[c]^	*Q*=0^[d]^	*Q*=−1^[c]^
Reaction 1: **3**_dd_+(Et_2_O)_2_H^+^→**4**_ddd_+2 Et_2_O			
**TS3_d_**	15.0	17.9	4.4
**4**_ddd_	4.0	−0.7	−26.6
Reaction 2: **3**_dp_+(Et_2_O)_2_H^+^→**4**_dpd_+2 Et_2_O			
**TS3_d_**	4.5	4.2	2.1
**4**_dpd_	−21.1	−30.7	−61.7

[a] Calculated at UB3LYP/BS2//UB3LYP/BS1+ZPE. [b] In kcal mol^−1^ relative to reactant complex. [c] Quartet spin state. [d] Triplet spin state.

As follows from the data shown in Table 1, upon reduction of the complex, the third proton transfer becomes energetically more exothermic for all cases and especially a high exothermicity of the reaction is found for the system with charge of *Q*=−1. The barrier heights also generally decrease upon changing the charge of the complex from *Q*=1 to *Q*=0 to *Q*=−1. The trend is seen for both examples; that is, distal protonation of **3**_dd_ as well as **3**_dp_. In particular, the barrier height for distal protonation of **3**_dd_ drops from 15.0 kcal mol^−1^ for the non‐reduced system, that is, the one shown in Figure 1, to 4.4 kcal mol^−1^ after double reduction. In addition, the driving force for the reaction increases steeply and is more exothermic for the doubly reduced system. Distal protonation of the [FeNHNH] system shows similar trends with larger exothermicity of the proton transfer reaction and lower barrier heights. These studies implicate that although triple protonation is a feasible process without the addition of external electrons, the overall reaction efficiency can be improved by adding electrons to the system. Most probably, the alternating addition of protons and electrons will be the energetically favourable process for nitrogen fixation. However, in the absence of available electron donors, the reaction can still proceed through sequential protonation, albeit considerably slower.

### Thermochemical cycles

**Figure 3:**
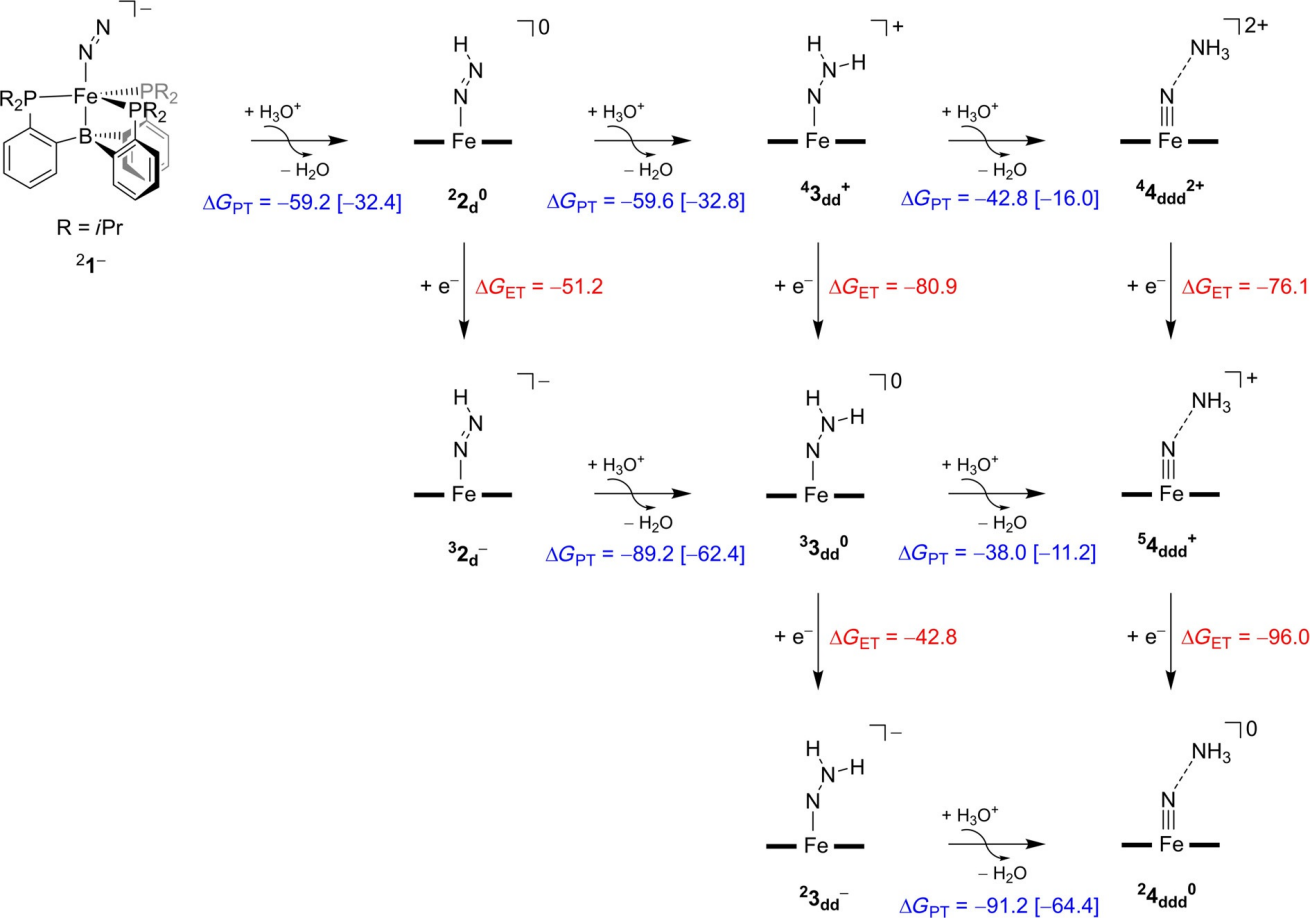
UB3LYP/BS2//UB3LYP/BS1 calculated thermochemical cycles for proton (horizontal) and electron (vertical) transfer from ^2^ **1** with free energies (Δ*G*) in kcal mol^−1^. Values in square brackets refer to data with (Et_2_O)_2_H^+^/2Et_2_O as proton transfer couple, while outside parenthesis is data for the H_3_O^+^/H_2_O couple.

To shed more light on the possible reaction pathways for electron‐ and proton‐transfer, we calculated individual reactions starting from ^2^ **1** for alternating and consecutive proton and electron transfer; the results are shown in Figure 3. To calculate the proton‐transfer energies, we used either the H_3_O^+^/H_2_O couple or the (Et_2_O)_2_H^+^/2Et_2_O couple to balance the reaction. However, the H_3_O^+^/H_2_O couple just shifts the relative free energies of proton transfer and makes all reaction pathways much more exergonic. As such, proton transfer will be a very fast process in a water solution. Using the (Et_2_O)_2_H^+^/2Et_2_O couple the proton transfers are all exergonic but by a mild amount, which means these reaction steps will be kinetically slower and individual intermediates may be trapped accordingly, in agreement with the experimental observations. Nevertheless, the proton transfer trends are the same regardless of the proton donor and the effect is only systematic.

Figure 3 starts from ^2^ **2**_d_^0^ and the proton transfer pathways are given from left to right and the electron transfer from top to bottom. Thus, proton transfer to form ^4^ **3**_dd_^+^ is exergonic by Δ*G*=−32.8 kcal mol^−1^ with (Et_2_O)_2_H^+^ as a proton source, whereas electron transfer to give ^3^ **2**_d_^−^ releases Δ*G*=−51.2 kcal mol^−1^. As such, electron transfer is energetically favoured over proton transfer in a diethyl ether solution. The proton transfer energies between ^2^ **1** ^−^ and ^2^ **2**_d_^0^ using (Et_2_O)_2_H^+^ as proton source matches the value from Figure 1 excellently and so does the proton‐transfer energy to give ^4^ **3**_dd_^+^. Overall, the three consecutive proton‐transfer steps from ^2^ **1** ^−^ are all exergonic and hence possible at room temperature. However, the proton‐transfer steps are energetically the most favourable from complexes with an overall charge of *Q*=−1; that is, ^2^ **1** ^−^, ^3^ **2**_d_^−^ and ^2^ **3**_dd_^−^. Most probably the overall charge of −1 gives these complexes high p*K*_a_ values and make them react fast with external protons. This means that alternating proton and electron transfer should give the most energetically favourable reaction process for conversion of N_2_ into ammonia and an iron‐nitrido complex.

On the other hand, the energetically most favourable reduction steps will be those where a positively charged complex is reduced to a neutral one; that is, ^4^ **3**_dd_^+^ and ^5^ **4**_ddd_^+^, which release Δ*G*_ET_=−80.9 and −96.0 kcal mol^−1^, respectively. Therefore, the nitrogen fixation reaction is possible through many different reaction pathways of proton and electron donations. We find a thermodynamic viable process of triple proton transfer without electron donation as well as ones where triple proton transfer is coupled to one or two electron transfers.

### Electronic changes during the reaction mechanism

**Figure 4:**
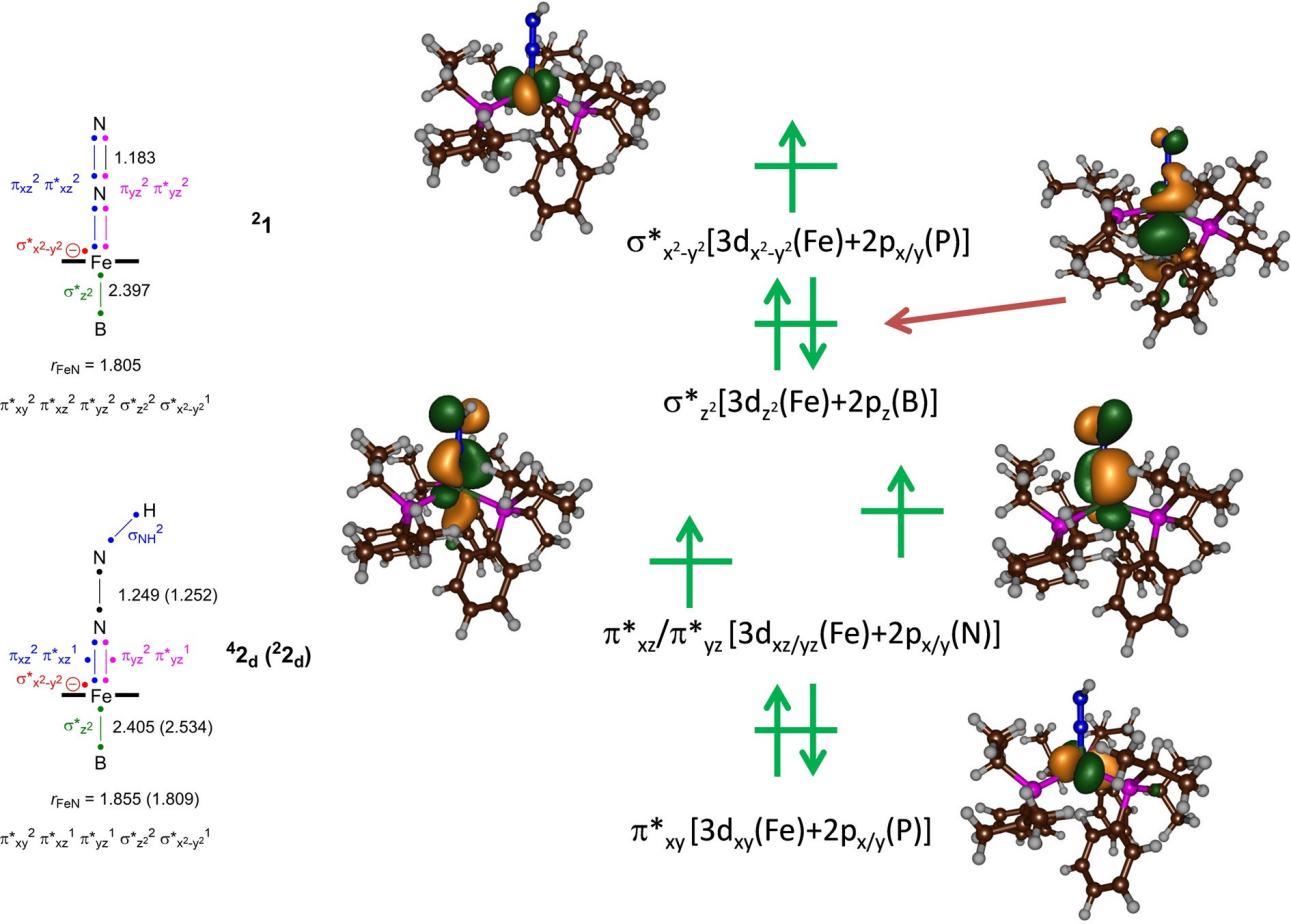
Valence bond description of structures ^2^ **1** and ^4,2^ **2**_d_: electrons are represented with a dot and a chemical bond is a line between two dots. Key valence orbitals of ^4,2^ **2** with orbital occupation in the quartet spin state are given on the right.

To understand the electronic changes during the proton‐transfer reaction, we analysed the electronic configurations and orbital descriptions of complexes **1**, **2**_d_, **3**_dd_ and **4**_ddd_ in detail. Figure 4 displays the high‐lying occupied and low‐lying virtual orbitals of **2**_d_ and a valence bond description of the electronic configurations of ^2^ **1** and ^2,4^ **2**_d_. These valence bond descriptions highlight the molecular orbitals that are formed and broken in each step and the formal charge distributions on the atoms. In particular, in the valence bond drawings, electrons are represented by a dot, and a line between two dots gives a molecular orbital occupied by two electrons. In the past we used these assessments to explain regioselectivities of reaction mechanisms and the origins of bifurcation processes and explained experimental reactivity trends.^18^ The key valence orbitals are determined by the 3d atomic orbitals on the metal that interact with neighbouring atoms. Thus, the low‐lying π*_xy_ orbital is a non‐bonding orbital in the plane of the phosphorus atoms and has a shape seen before for nonheme iron complexes.^13^, ^19^ A degenerate set of orbitals for the interactions of the metal with the two nitrogen atoms leads to a set of bonding (π_*xz*_/π_*yz*_, not shown in Figure 4) and antibonding (π*_xz_ and π*_yz_) orbitals that cover the Fe, N_d_ and N_p_ atoms. In blue, we give the four electrons that occupy the π_*xz*_ and π*_xz_ orbitals and in purple those for the π_*yz*_ and π*_yz_ orbitals. Even in the reactant complex ^2^ **1** there is considerable mixing of the iron and N_2_ π‐type orbitals and, consequently, also charge transfer from nitrogen to metal. In the reactant complex the π*_xz_ and π*_yz_ orbitals are doubly occupied and, as a result, the Fe−N bond length is short and corresponds to a formal single bond with a distance of 1.805 Å. The atomic 3d_*z*2_ orbital of iron is involved in a bonding orbital with the 2p_*z*_ on boron to form the σ*_z2_ orbital, which also gives considerable charge transfer from iron to boron of *Q*=−0.42 and a relatively short Fe−B distance of 2.397 Å. The highest lying 3d orbital on iron is singly occupied (σ*_x2‐*y*2_) and gives ^2^ **1** an overall orbital occupation of π*_xy_^2^ π*_xz_^2^ π*_yz_^2^ σ*_z2_^2^ σ*_x2‐*y*2_^1^ and a formal 3d^9^ configuration. The valence bond structure, therefore, gives eight electrons along the Fe−N bond, representing the occupation of the π_*xz*_^2^ π*_xz_^2^ π_*yz*_^2^ π*_yz_^2^ orbitals. The Fe−B interaction is formally a single bond through occupation of the σ*_z2_ orbital with two electrons (shown in green in the valence bond drawing), whereas the unpaired electron in σ*_x2‐*y*2_ is given in red.

Upon protonation of the terminal nitrogen atom, as in ^2,4^ **2**, the degeneracy of the π*_xz_/π*_yz_ couple is lost. Specifically, the 2p_*y*_ orbital on the terminal nitrogen atom splits off from the molecular π_*yz*_/π*_yz_ interaction and forms a σ_NH_ bond with the 1s orbital of the incoming proton. Electronically, the two electrons that form the σ_NH_ orbital both originate from the transfer of one electron from the π*_xz_ and π*_yz_ orbitals into σ_NH_. As a result of this, the quartet spin state drops in energy and becomes the most favourable configuration, with orbital occupation σ_NH_^2^ π*_xy_^2^ π*_xz_^1^ π*_yz_^1^ σ*_z2_^2^ σ*_x2‐*y*2_^1^. The loss of π*_xz_/π*_yz_ electrons in **2**_d_ leads to elongation of the N−N distance to 1.25 Å in both the quartet and doublet spin states. However, the Fe−B interaction stays intact at a distance of 2.405 (2.534) Å in ^4^ **2**_d_ (^2^ **2**_d_), respectively, due to the still doubly occupied σ*_z2_ orbital.

**Figure 5:**
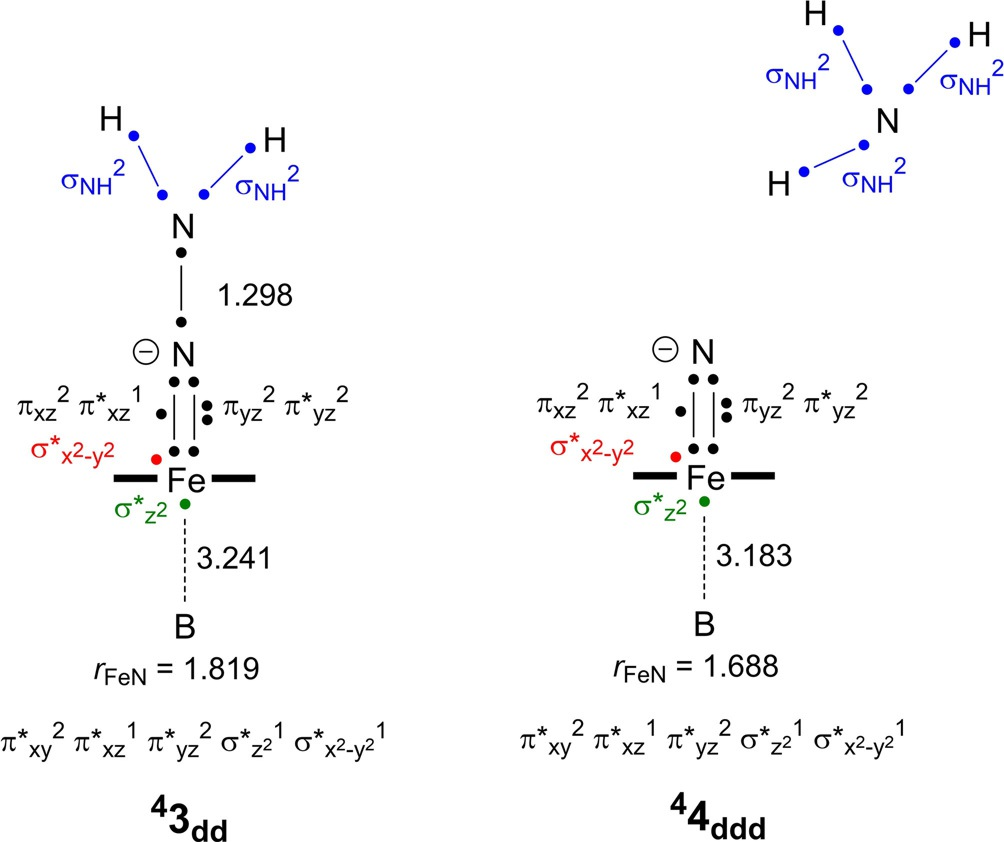
Valence bond description of structures ^4^ **3**_dd_ and ^4^ **4**_ddd_: electrons are represented with a dot and a chemical bond is a line between two dots.

The second proton transfer from ^2,4^ **2**_d_ to form ^4^ **3**_dd_ is electronically depicted in Figure 5. In ^4^ **3**_dd_ the N=N double bond is broken and charge density migrates from nitrogen to iron strengthening the Fe−N_p_ bond. As a consequence of strengthening of the Fe−N bond, the Fe−B bond is weakened or broken and the σ*_z2_ orbital converts into an atomic orbital. It is seen that the Fe−B distance elongates to well over 3 Å in both structures **3** and **4**. At the same time, one electron from σ*_z2_ is promoted into the π*_yz_ orbital to get the overall orbital occupation π*_xy_^2^ π*_xz_^1^ π*_yz_^2^ σ*_z2_^1^ σ*_x2‐*y*2_^1^. In the final proton transfer to the terminal nitrogen atom, an ammonia molecule is formed and the Fe−N distance reduces to 1.688 Å, but the electronic configuration stays the same.

## Conclusions

We present a series of density functional theory calculations on the electron‐ and proton‐transfer processes in a mononuclear iron model of nitrogenase. We calculate barrier heights for proton transfer in the absence of an electron donor and show a feasible process with three consecutive proton transfer barriers in an overall exothermic process. Protonation is favoured on the distal nitrogen atom so that triple proton transfer forms one ammonia molecule and an iron‐nitrido complex. Tests on proximal protonation find barriers that are higher in energy due to stereochemical interactions and shielding of the proximal site. We finally tested proton transfer from complexes with different overall charge and show that the energetically favourable process is consecutive proton and electron transfer. However, alternative pathways are energetically accessible and cannot be ruled out. The calculations point out that proton and electron transfer do not necessarily need to happen consecutively because the energetics are also favourable for multiple proton transfer without electron transfer. As such, multiple pathways for the formation of ammonia from molecular nitrogen are possible, which give the enzyme sufficient flexibility to operate under low acid or low reduction conditions.

## Experimental Section

All computational studies presented here used density functional theory methods as implemented in the Gaussian 09 program package,^20^ and extensively benchmarked and calibrated against experimental data.^21^ These calculations on nonheme iron complexes reproduced experimental rate constants and Hammett trends well. Our model was based on the [(TBP)FeN_2_]^−^ structure as described by Peters et al.,11a and the mechanisms for proton transfer were studied with protonated diethyl ether dimer, (Et_2_O)_2_H^+^. As the overall charge of our chemical system ranges from −1 to +2 we did full geometry optimisations with a solvent model included; namely, the polarised continuum model (CPCM),^22^ with dielectric constant mimicking tetrahydrofuran, which was the experimentally used solvent. Initial geometry optimisations, frequencies and geometry scans were performed using the hybrid density functional method UB3LYP^23^ in combination with basis set BS1: LACVP basis set with electron core potential on iron and 6‐31G on the rest of the atoms.^24^ Energetics were improved by single‐point calculations with basis set BS2: triple‐ζ LACV3P+ basis set with electron core potential on iron and 6‐311+G* on the rest of the atoms. All energies reported in this work are Δ*E*+ZPE data with energies at BS2 and zero point corrections with BS1.

To test the accuracy of our optimisation algorithm, we repeated the geometry optimisations of local minima and some of the transition states using tight convergence criteria in Gaussian. In none of these cases; however, did the energy change by more than 10^−6^ hartrees and neither did the optimised geometries change significantly. The reproducibility of the calculations was investigated by reoptimising the proton‐transfer reaction from ^2^ **1** to ^2^ **2** with the PBE0 density functional method.^25^ As follows (see the Supporting Information, Figure S17), the structure and relative energies are very similar to those found with B3LYP and therefore the project was finished with B3LYP only.

## Conflict of interest

The authors declare no conflict of interest.

## Supporting information

As a service to our authors and readers, this journal provides supporting information supplied by the authors. Such materials are peer reviewed and may be re‐organized for online delivery, but are not copy‐edited or typeset. Technical support issues arising from supporting information (other than missing files) should be addressed to the authors.

Supplementary
