# Supplementary material for: Nitrogen Reduction to Ammonia on a Biomimetic Mononuclear Iron Centre: Insights into the Nitrogenase Enzyme
Source: Chemistry. 2017 Dec 14;24(20):5293–302. doi: 10.1002/chem.201704688 (PMC5915742; doi:10.1002/chem.201704688)
Supplement: Supplementary file 1 — Supplementary [file CHEM-24-5293-s001.pdf]

CHEMISTRY
A European Journal

Supporting Information

Nitrogen Reduction to Ammonia on a Biomimetic Mononuclear Iron Centre: Insights into the Nitrogenase Enzyme

Monika A. Kaczmarek,[a, b] Abheek Malhotra,[a] G. Alex Balan,[a] Amy Timmins,[a] and Sam P. de Visser*[a]

chem_201704688_sm_miscellaneous_information.pdf

Methods

All computational studies presented here used density functional theory methods as implemented in the Gaussian-09 program package.[1] Our model was based on the $[(TBP)FeN_2]^-$ structure as described by Peters et al,[2] and the mechanisms for proton transfer were studied with protonated diethyl ether dimer, $(Et_2O)_2H^+$. As the overall charge of our chemical system ranges from –1 to +2 we did full geometry optimizations with a solvent model included, namely the polarized continuum model (CPCM),[3] with dielectric constant mimicking tetrahydrofuran; the experimentally used solvent. Initial geometry optimizations, frequencies and geometry scans were performed using the hybrid density functional method UB3LYP[4] in combination with basis set BS1: LACVP basis set with electron core potential on iron and 6-31G on the rest of the atoms.[5] Energetics were improved by single points calculations with basis set BS2: triple-ζ LACV3P+ basis set with electron core potential on iron and 6-311+G* on the rest of the atoms. All energies reported in this work are ΔE+ZPE data with energies at BS2 and zero point corrections with BS1.

To test the accuracy of our optimization algorithm we repeated the geometry optimizations of local minima and some of the transition states using tight convergence criteria. In none of these cases; however, the energy changed by more than 10^{-6} hartrees and neither did the optimized geometries change significantly.

To test the effect of the density functional method on the spin-state energies we reoptimized a number of structures with the PBE0 density functional method[6], which only gave minor differences in structures and relative energies.

References:

[1] Gaussian-09, Revision C.01, M. J. Frisch, G. W. Trucks, H. B. Schlegel, G. E. Scuseria, M. A. Robb, J. R. Cheeseman, G. Scalmani, V. Barone, B. Mennucci, G. A. Petersson, H. Nakatsuji, M. Caricato, X. Li, H. P. Hratchian, A. F. Izmaylov, J. Bloino, G. Zheng, J. L. Sonnenberg, M. Hada, M. Ehara, K. Toyota, R. Fukuda, J. Hasegawa, M. Ishida, T. Nakajima, Y. Honda, O. Kitao, H. Nakai, T. Vreven, J. A. Montgomery, Jr., J. E. Peralta, F. Ogliaro, M. Bearpark, J. J. Heyd, E. Brothers, K. N. Kudin, V. N. Staroverov, T. Keith, R. Kobayashi, J. Normand, K. Raghavachari, A. Rendell, J. C. Burant, S. S. Iyengar, J. Tomasi, M. Cossi, N. Rega, J. M. Millam, M. Klene, J. E. Knox, J. B. Cross, V. Bakken, C. Adamo, J. Jaramillo, R. Gomperts, R. E. Stratmann, O. Yazyev, A. J. Austin, R. Cammi, C. Pomelli, J. W. Ochterski, R. L. Martin, K. Morokuma, V. G. Zakrzewski, G. A. Voth, P. Salvador, J. J. Dannenberg, S. Dapprich, A. D. Daniels, O. Farkas, J. B. Foresman, J. V. Ortiz, J. Cioslowski, D. J. Fox, Gaussian, Inc., Wallingford CT, 2010.

[2] J. S. Anderson, G. E. Cutsail III, J. Rittle, B. A. Connor, W. A. Gunderson, L. Zhang, B. M. Hoffman, J. C. Peters, *J. Am. Chem. Soc*. **2015**, *137*, 7803–7809.

[3] J. Tomasi, B. Mennucci, R. Cammi, *Chem. Rev*. **2005**, *105*, 2999–309.

[4] a) A. D. Becke, *J. Chem. Phys*. **1993**, *98*, 5648–5652; b) C. Lee, W. Yang, R. G. Parr, *Phys. Rev. B* **1988**, *37*, 785–789.

[5] a) P. J. Hay, W. R. Wadt, *J. Chem. Phys*. **1985**, *82*, 270–283; b) W. J. Hehre, R. Ditchfield, J. A. Pople, *J. Chem. Phys*. **1972**, *56*, 2257–2261.

[6] C. Adamo, V. Barone, *J. Chem. Phys*. **1999**, *110*, 6158–6169.

WILEY-VCH

 WILEY-VCH

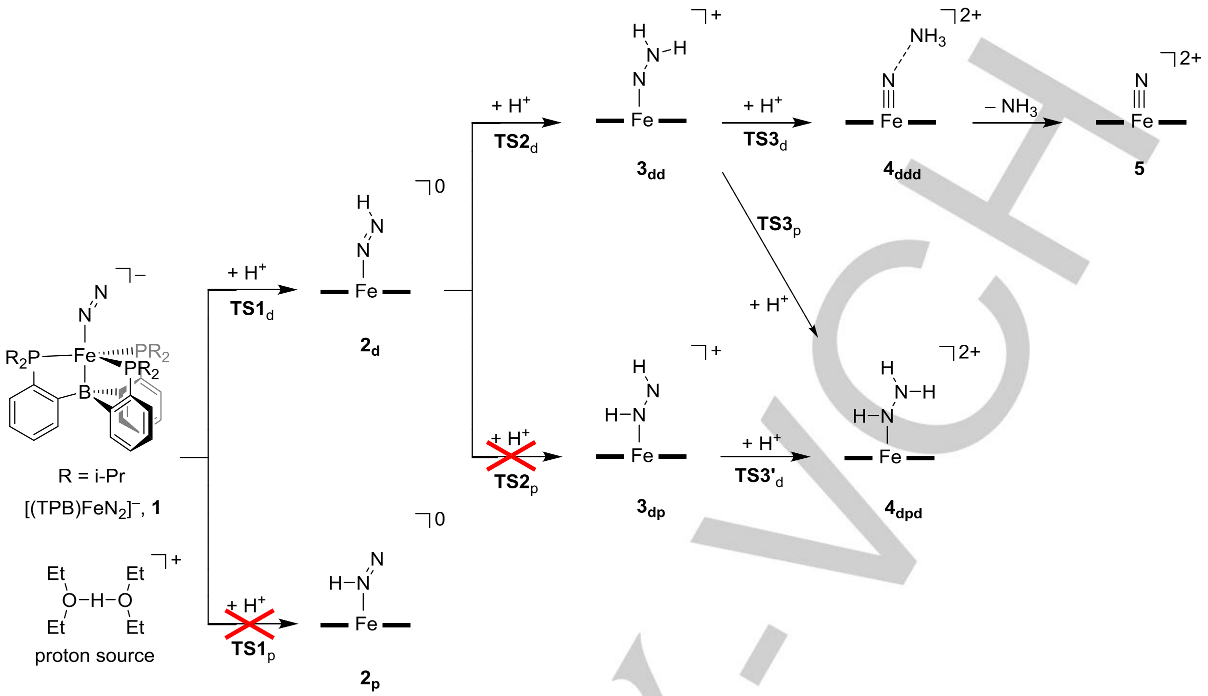

Scheme S1: Calculated reaction scheme with nomenclature of local minima and transition states for proton transfer to $[(TPB)FeN_2]^-$ in the absence of a redox partner.

WILEY-VCH

Table S1: Absolute energies, zero-point energies and free energies (in au) of optimized geometries as described in this work. Data for the non-reduced system.

		E [au]	ZPE [au]	G [au]	E [au]	E [au]	ZPE [au]	G [au]
		THF, BS1	THF, BS1	THF, BS1	THF, BS2	tight, BS1	tight, BS1	tight, BS1
^{2}[(TPB)Fe-N=N]$^{-1}$	$^{2}\mathbf{1}$	-2685.88364	0.83205	-2685.13467	-2686.78155	-2685.88364	0.83207	-2685.13459
^{4}[(TPB)Fe-N=N]$^{-1}$	$^{4}\mathbf{1}$	-2685.87323	0.82878	-2685.13048	-2686.77148	-2685.87323	0.82878	-2685.13048
^{6}[(TBP)Fe-N=N]$^{-1}$	$^{6}\mathbf{1}$	-2685.84771	0.82921	-2685.10567	-2686.74070	-2685.84771	0.82921	-2685.10567
^{2}[(TBP)Fe-N=NH]^{0}	$^{2}\mathbf{2}_{\mathbf{d}}$	-2686.36263	0.84356	-2685.60277	-2687.24520	-2686.36263	0.84356	-2685.60278
^{4}[(TBP)Fe-N=NH]^{0}	$^{4}\mathbf{2}_{\mathbf{d}}$	-2686.36062	0.84299	-2685.60219	-2687.24550	-2686.36062	0.84299	-2685.60218
^{6}[(TBP)Fe-N=NH]^{0}	$^{6}\mathbf{2}_{\mathbf{d}}$	-2686.32711	0.84324	-2685.56848	-2687.20685			
^{2}[(TBP)Fe=N-NH$_2$]$^{+}$	$^{2}\mathbf{3}_{\mathbf{dd}}$	-2686.82090	0.86003	-2686.04357	-2687.68683	-2686.82090	0.86003	-2686.04357
^{4}[(TBP)Fe=N-NH$_2$]$^{+}$	$^{4}\mathbf{3}_{\mathbf{dd}}$	-2686.84994	0.85989	-2686.07453	-2687.71415	-2686.84994	0.85989	-2686.07453
^{6}[(TBP)Fe=N-NH$_2$]$^{+}$	$^{6}\mathbf{3}_{\mathbf{dd}}$	-2686.82597	0.85857	-2686.05288	-2687.68934	-2686.82597	0.85859	-2686.05285
^{2}[(TBP)Fe-N(H)NH]$^{+}$	$^{2}\mathbf{3}_{\mathbf{dp}}$	-2686.81446	0.86071	-2686.03605	-2687.68415	-2686.81446	0.86071	-2686.03606
^{4}[(TBP)Fe-N(H)NH]$^{+}$	$^{4}\mathbf{3}_{\mathbf{dp}}$	-2686.84486	0.85942	-2686.06902	-2687.71686	-2686.84486	0.85943	-2686.06900
^{6}[(TBP)Fe-N(H)NH]$^{+}$	$^{6}\mathbf{3}_{\mathbf{dp}}$	-2686.82586	0.85824	-2686.05243	-2687.69543	-2686.82586	0.85824	-2686.05240
^{2}[(L)Fe=N---NH$_3$]$^{2+}$	$^{2}\mathbf{4}_{\mathbf{ddd}}$	-2687.25045	0.87579	-2686.45523	-2688.11042	-2687.25045	0.87579	-2686.45522
^{4}[(L)Fe=N---NH$_3$]$^{2+}$	$^{4}\mathbf{4}_{\mathbf{ddd}}$	-2687.29642	0.87481	-2686.50349	-2688.15834	-2687.29642	0.87482	-2686.50348
^{6}[(L)Fe=N---NH$_3$]$^{2+}$	$^{6}\mathbf{4}_{\mathbf{ddd}}$	-2687.28775	0.87349	-2686.49869	-2688.14984	-2687.28775	0.87348	-2686.49872
^{2}[(L)Fe=N]$^{2+}$	$^{2}\mathbf{5}$	-2630.66522	0.83549	-2629.90922	-2631.50599			
^{4}[(L)Fe=N]$^{2+}$	$^{4}\mathbf{5}$	-2630.75764	0.83501	-2630.00154	-2631.60713			
^{6}[(L)Fe=N]$^{2+}$	$^{6}\mathbf{5}$	-2630.75721	0.83488	-2630.00197	-2631.61308			

Table S2: Absolute energies, zero-point energies and free energies (in au) of optimized geometries as described in this work. Data for the non-reduced system.

	E [au]	ZPE [au]	G [au]	E [au]	E [au]	ZPE [au]	G [au]
	THF, BS1	THF, BS1	THF, BS1	THF, BS2	tight, BS1	tight, BS1	tight, BS1
$(EtO)_2H^+$	-467.64537	0.28743	-467.40363	-467.86316	-467.64537	0.28744	-467.40359
$(EtO)_2$	-467.20464	0.27620	-466.97480	-467.45006	-467.20464	0.27620	-466.97479
NH_3	-56.53716	0.03430	-56.52193	-56.53716	-56.53716	0.03429	-56.52194
H_2O	-76.39429	0.02060	-76.39202	-76.45174			
H_3O^+	-76.78526	0.03385	-76.77071	-76.82248			

WILEY-VCH

Table S3: Absolute energies, zero-point energies and free energies (in au) of optimized geometries as described in this work. Data for the singly-reduced system.

		E [au]	ZPE [au]	G [au]	E [au]
		THF, BS1	THF, BS1	THF, BS1	THF, BS2
^{1}[(TBP)Fe-N=NH]$^-$	^{1}**2**$_d$$^-$	-2686.41982	0.84084	-2685.66160	-2687.31395
^{3}[(TBP)Fe-N=NH]$^-$	^{3}**2**$_d$$^-$	-2686.43265	0.84041	-2685.67543	-2687.32419
^{5}[(TBP)Fe-N=NH]$^-$	^{5}**2**$_d$$^-$	-2686.40419	0.83950	-2685.65148	-2687.29431
^{1}[(TBP)Fe=N-NH$_2$]^0	^{1}**3**$_{dd}$^{0}	-2686.93526	0.85619	-2686.16254	-2687.46332
^{3}[(TBP)Fe=N-NH$_2$]^0	^{3}**3**$_{dd}$^{0}	-2686.96452	0.85667	-2686.19232	-2687.83981
^{5}[(TBP)Fe=N-NH$_2$]^0	^{5}**3**$_{dd}$^{0}	-2686.94210	0.85548	-2686.17304	-2687.81777
^{1}[(L)Fe=N---NH$_3$]$^+$	^{1}**4**$_{ddd}$$^+$	-2687.40689	0.86877	-2686.62369	-2688.27943
^{3}[(L)Fe=N---NH$_3$]$^+$	^{3}**4**$_{ddd}$$^+$	-2687.39572	0.87262	-2686.60675	-2688.25750
^{5}[(L)Fe=N---NH$_3$]$^+$	^{5}**4**$_{ddd}$$^+$	-2687.41047	0.87321	-2686.62232	-2688.27478
^{1}[(L)Fe=N]$^+$	^{1}**5**$^+$	-2630.86493	0.83288	-2630.11173	-2631.70105
^{3}[(L)Fe=N]$^+$	^{3}**5**$^+$	-2630.86538	0.83293	-2630.11275	-2631.70158
^{5}[(L)Fe=N]$^+$	^{5}**5**$^+$	-2630.84869	0.83289	-2630.09721	-2631.67402

WILEY-VCH

Table S4: Absolute energies, zero-point energies and free energies (in au) of optimized geometries as described in this work. Data for the doubly-reduced system.

		E [au]	ZPE [au]	G [au]	E [au]
		THF, BS1	THF, BS1	THF, BS1	THF, BS2
^{2}[(TBP)Fe=N-NH$_2$]$^-$	$^{2}\mathbf{3_{dd}}^-$	-2687.01624	0.85306	-2686.24690	-2687.90514
^{4}[(TBP)Fe=N-NH$_2$]$^-$	$^{4}\mathbf{3_{dd}}^-$	-2687.02062	0.85277	-2686.25347	-2687.90550
^{6}[(TBP)Fe=N-NH$_2$]$^-$	$^{6}\mathbf{3_{dd}}^-$	-2687.00138	0.85275	-2686.23469	-2687.88365
^{2}[(TBP)Fe=N---NH$_3$]^0	$^{2}\mathbf{4_{ddd}^0}$	-2687.53728	0.86717	-2686.75581	-2688.42103
^{4}[(TBP)Fe=N---NH$_3$]^0	$^{4}\mathbf{4_{ddd}^0}$	-2687.53259	0.86680	-2686.75383	-2688.40571
^{6}[(TBP)Fe=N---NH$_3$]^0	$^{6}\mathbf{4_{ddd}^0}$	-2687.52455	0.86626	-2686.74646	-2688.39885
^{2}[(TBP)Fe=N]^0	$^{2}\mathbf{5^0}$	-2630.99392	0.83108	-2630.24258	-2631.84247
^{4}[(TBP)Fe=N]^0	$^{4}\mathbf{5^0}$	-2630.98623	0.82979	-2630.23983	-2631.82898
^{6}[(TBP)Fe=N]^0	$^{6}\mathbf{5^0}$	-2630.97906	0.82966	-2630.23254	-2631.81886

WILEY-VCH

Table S5: Relative (free) energies (in kcal mol^{-1}) of optimized geometries as described in this work. Spin state ordering and relative energies data for the non-reduced system. Optimizations done using default (def) and tight convergence criteria.

	ΔE	ΔE+ZPE	ΔG	ΔE	ΔE+ZPE	ΔG	ΔE	ΔE+ZPE
	BS1,def	BS1,def	BS1,def	BS2	BS2	BS2	BS1,tight	BS1,tight
$^{2}\mathbf{1}$	0.00	0.00	0.00	0.00	0.00	0.00	0.00	0.00
$^{4}\mathbf{1}$	6.53	4.47	2.62	6.32	4.27	2.42	6.53	4.47
$^{6}\mathbf{1}$	22.54	20.76	18.19	25.63	23.85	21.29	22.54	20.75
$^{2}\mathbf{2_d}$	0.00	0.00	0.00	0.00	0.00	0.00	0.00	0.00
$^{4}\mathbf{2_d}$	1.26	0.90	0.37	-0.19	-0.55	-1.08	1.26	0.90
$^{6}\mathbf{2_d}$	22.28	22.08	21.52	24.07	23.87	23.30		
$^{2}\mathbf{3_{dd}}$	0.00	0.00	0.00	0.00	0.00	0.00	0.00	0.00
$^{4}\mathbf{3_{dd}}$	-18.23	-18.32	-19.43	-17.14	-17.24	-18.34	-18.23	-18.32
$^{6}\mathbf{3_{dd}}$	-3.18	-4.10	-5.84	-1.58	-2.49	-4.24	-3.18	-4.09
$^{2}\mathbf{3_{dp}}$	0.00	0.00	0.00	0.00	0.00	0.00	0.00	0.00
$^{4}\mathbf{3_{dp}}$	-19.08	-19.89	-20.69	-20.53	-21.34	-22.14	-19.08	-19.88
$^{6}\mathbf{3_{dp}}$	-7.15	-8.70	-10.28	-7.08	-8.63	-10.20	-7.15	-8.70
$^{2}\mathbf{4_{ddd}}$	0.00	0.00	0.00	0.00	0.00	0.00	0.00	0.00
$^{4}\mathbf{4_{ddd}}$	-28.85	-29.46	-30.28	-30.07	-30.68	-31.51	-28.85	-29.46
$^{6}\mathbf{4_{ddd}}$	-23.41	-24.85	-27.27	-24.74	-26.18	-28.61	-23.41	-24.85

WILEY-VCH

Table S6: Relative (free) energies (in kcal mol^{-1}) of optimized geometries as described in this work. Spin state ordering and relative energies data for the singly-reduced system. Optimizations done using default (def) and tight convergence criteria.

	ΔE	ΔE+ZPE	ΔG	ΔE	ΔE+ZPE	ΔG
	BS1,def	BS1,def	BS1,def	BS2	BS2	BS2
$^{1}\mathbf{2_d}^{-}$	8.05	8.32	8.68	6.43	6.70	7.06
$^{3}\mathbf{2_d}^{-}$	0.00	0.00	0.00	0.00	0.00	0.00
$^{5}\mathbf{2_d}^{-}$	17.86	17.29	15.03	18.75	18.19	15.92
$^{1}\mathbf{3_{dd}}^{\mathbf{0}}$	18.36	18.06	18.69	236.25	235.95	236.58
$^{3}\mathbf{3_{dd}}^{\mathbf{0}}$	0.00	0.00	0.00	0.00	0.00	0.00
$^{5}\mathbf{3_{dd}}^{\mathbf{0}}$	14.06	13.32	12.10	13.83	13.08	11.87
$^{1}\mathbf{4_{ddd}}^{+}$	0.00	0.00	0.00	0.00	0.00	0.00
$^{3}\mathbf{4_{ddd}}^{+}$	7.01	9.43	10.63	13.76	16.18	17.39
$^{5}\mathbf{4_{ddd}}^{+}$	-2.25	0.54	0.86	2.92	5.70	6.03
$^{1}\mathbf{5}^{+}$	0.00	0.00	0.00	0.00	0.00	0.00
$^{3}\mathbf{5}^{+}$	-0.28	-0.25	-0.64	-0.33	-0.30	-0.69
$^{5}\mathbf{5}^{+}$	10.19	10.19	9.11	16.96	16.96	15.88

WILEY-VCH

Table S7: Relative (free) energies (in kcal mol^{-1}) of optimized geometries as described in this work. Spin state ordering and relative energies data for the doubly-reduced system. Optimizations done using default (def) and tight convergence criteria.

	ΔE	ΔE+ZPE	ΔG	ΔE	ΔE+ZPE	ΔG	ΔE	ΔE+ZPE
	BS1,def	BS1,def	BS1,def	BS2	BS2	BS2	BS1,tight	BS1,tight
$^{2}\mathbf{3}_{\mathbf{dd}}^{-}$	2.75	2.93	4.12	0.23	0.41	1.60		
$^{4}\mathbf{3}_{\mathbf{dd}}^{-}$	0.00	0.00	0.00	0.00	0.00	0.00		
$^{6}\mathbf{3}_{\mathbf{dd}}^{-}$	12.07	12.06	11.78	13.71	13.70	13.42		
$^{2}\mathbf{4}_{\mathbf{ddd}}^{\mathbf{0}}$	0.00	0.00	0.00	0.00	0.00	0.00	0.00	0.00
$^{4}\mathbf{4}_{\mathbf{ddd}}^{\mathbf{0}}$	2.95	2.71	1.24	9.61	9.38	7.91	2.95	2.72
$^{6}\mathbf{4}_{\mathbf{ddd}}^{\mathbf{0}}$	7.99	7.42	5.87	13.92	13.35	11.79	7.99	7.42
$^{2}\mathbf{5}^{\mathbf{0}}$	0.00	0.00	0.00	0.00	0.00	0.00	0.00	0.00
$^{4}\mathbf{5}^{\mathbf{0}}$	4.83	4.02	1.73	8.47	7.66	5.36	4.83	4.01
$^{6}\mathbf{5}^{\mathbf{0}}$	9.33	8.43	6.30	14.82	13.93	11.79	9.33	8.39

WILEY-VCH

Table S8: Absolute energies, zero-point energies and free energies (in au) of optimized geometries as described in this work. Data for the non-reduced system.

		E [au]	ZPE [au]	G [au]	E [au]	E [au]	ZPE [au]	G [au]
		THF, BS1	THF, BS1	THF, BS1	THF, BS2	tight	tight	tight
**Step 1:**								
^{2}[(L)Fe-N=N---$(Et_2O)_2H^+$]^0	$^{2}\mathbf{1}_{RC}$	-3153.545845	1.120677	-3152.534818	-3154.656624	-3153.545845	1.120673	-3152.534819
^{4}[(L)Fe-N=N---$(Et_2O)_2H^+$]^0	$^{4}\mathbf{1}_{RC}$	-3153.530935	1.118146	-3152.524973	-3154.645109	-3153.530935	1.118150	-3152.524912
^{6}[(L)Fe-N=N---$(Et_2O)_2H^+$]^0	$^{6}\mathbf{1}_{RC}$	-3153.508454	1.118661	-3152.501931	-3154.607707	-3153.508454	1.118653	-3152.501948
$^{2}\mathbf{TS1}_{PT}$	$^{2}\mathbf{TS1}_{d}$	-3153.534134	1.122133	-3152.519602	-3154.650210	-3153.534134	1.122136	-3152.519572
^{2}[(L)Fe-N=NH---$(Et_2O)_2$]$^+$	$^{2}\mathbf{1}_{P}$	-3153.575717	1.121863	-3152.566745	-3154.695044	-3153.575717	1.121863	-3152.566749
^{6}[(L)Fe-N=NH---$(Et_2O)_2$]$^+$	$^{6}\mathbf{1}_{P}$	-3153.548059	1.120110	-3152.543555	-3154.669047	-3153.548059	1.120111	-3152.543541
**Step 2 path 1:**								
^{2}[(L)Fe-N=NH---$(Et_2O)_2H^+$]$^+$	$^{2}\mathbf{2}_{RC}$	-3154.011171	1.132144	-3152.990494	-3155.109877	-3154.011171	1.132147	-3152.990462
^{4}[(L)Fe-N=NH---$(Et_2O)_2H^+$]$^+$	$^{4}\mathbf{2}_{RC}$	-3154.009347	1.131435	-3152.991085	-3155.110141	-3154.009347	1.131418	-3152.991260
^{6}[(L)Fe-N=NH---$(Et_2O)_2H^+$]$^+$	$^{6}\mathbf{2}_{RC}$	-3153.987435	1.130480	-3152.971178	-3155.086608	-3153.987435	1.130478	-3152.971198
$^{2}\mathbf{TS2}_{PT}$	$^{2}\mathbf{TS2}_{d}$	-3154.000198	1.134449	-3152.973898	-3155.102000	-3154.000198	1.134450	-3152.973887
$^{4}\mathbf{TS2}_{PT}$	$^{4}\mathbf{TS2}_{d}$	-3154.000808	1.133872	-3152.975638	-3155.104648			
$^{6}\mathbf{TS2}_{PT}$	$^{6}\mathbf{TS2}_{d}$	-3153.977434	1.132563	-3152.955929	-3155.081710	-3153.977434	1.132556	-3152.955980
^{2}[(L)Fe-N-NH_2---$(Et_2O)_2$]$^+$	$^{2}\mathbf{2}_{P}$	-3154.044666	1.138830	-3153.014927	-3155.141126	-3154.044666	1.138831	-3153.014925
^{6}[(L)Fe-N-NH_2---$(Et_2O)_2$]$^+$	$^{6}\mathbf{2}_{P}$	-3154.041873	1.137259	-3153.019294	-3155.138117	-3154.041873	1.137264	-3153.019257

ACCEPTED MANUSCRIPT

Table S9: Absolute energies, zero-point energies and free energies (in au) of optimized geometries as described in this work.

		E [au]	ZPE [au]	G [au]	E [au]
**Step 3 path 1:**		THF, BS1	THF, BS1	THF, BS1	THF, BS2
$^{2}[(L)Fe{=}N\text{-}NH_2\text{---}(Et_2O)_2H^+]^{2+}$	$^{2}\mathbf{3}_{\mathbf{RC}}$	-3154.466172	1.148386	-3153.439196	-3155.547897
$^{4}[(L)Fe{=}N\text{-}NH_2\text{---}(Et_2O)_2H^+]^{2+}$	$^{4}\mathbf{3}_{\mathbf{RC}}$	-3154.493043	1.148317	-3153.464656	-3155.574715
$^{6}[(L)Fe{=}N\text{-}NH_2\text{---}(Et_2O)_2H^+]^{2+}$	$^{6}\mathbf{3}_{\mathbf{RC}}$	-3154.465124	1.146962	-3153.432439	-3155.546056
$^{2}\mathbf{TS3}_{\mathbf{PT}}$	$^{2}\mathbf{TS3}_{\mathbf{d}}$	-3154.441450	1.151909	-3153.395312	-3155.526385
$^{4}\mathbf{TS3}_{\mathbf{PT}}$	$^{4}\mathbf{TS3}_{\mathbf{d}}$	-3154.467832	1.150404	-3153.427620	-3155.553033
$^{2}[(L)Fe{=}N\text{-}NH_3\text{---}(Et_2O)_2]^{2+}$	$^{2}\mathbf{3}_{\mathbf{P}}$	-3154.465597	1.152809	-3153.421738	-3155.552113
$^{4}[(L)Fe{=}N\text{-}NH_3\text{---}(Et_2O)_2]^{2+}$	$^{\mathbf{4}}\mathbf{3}_{\mathbf{P}}$	-3154.482673	1.151992	-3153.440506	-3155.572012
$^{6}[(L)Fe{=}N\text{-}NH_3\text{---}(Et_2O)_2]^{2+}$	$^{\mathbf{6}}\mathbf{3}_{\mathbf{P}}$	-3154.483124	1.153321	-3153.440815	-3155.574329
**Step 3 path 2:**					
$^{2}[(L)Fe{=}N\text{-}NH_2\text{---}(EtO)_2H^+]^{1+}$	$^{2}\mathbf{3}_{\mathbf{RC}}^{+}$	-3154.571222	1.148044	-3153.533703	-3155.674336
$^{4}[(L)Fe{=}N\text{-}NH_2\text{---}(EtO)_2H^+]^{1+}$	$^{4}\mathbf{3}_{\mathbf{RC}}^{+}$	-3154.610487	1.144478	-3153.580712	
$^{6}[(L)Fe{=}N\text{-}NH_2\text{---}(EtO)_2H^+]^{1+}$	$^{6}\mathbf{3}_{\mathbf{RC}}^{+}$	-3154.589889	1.144074	-3153.560527	-3155.682095
$^{2}\mathbf{TS3}_{\mathbf{PT}}$	$^{2}\mathbf{TS3}_{\mathbf{d}}^{+}$	-3154.563104	1.151256	-3153.514596	-3155.666316
$^{4}\mathbf{TS3}_{\mathbf{PT}}$	$^{4}\mathbf{TS3}_{\mathbf{d}}^{+}$	-3154.594479	1.147684	-3153.556456	-3155.692484
$^{6}\mathbf{TS3}_{\mathbf{PT}}$	$^{6}\mathbf{TS3}_{\mathbf{d}}^{+}$	-3154.570888	1.147057	-3153.532884	-3155.669138
$^{2}[(L)Fe{=}N\text{-}NH_3\text{---}(EtO)_2]^{1+}$	$^{2}\mathbf{3}_{\mathbf{P}}^{+}$	-3154.610715	1.153894	-3153.563137	-3155.711071
$^{4}[(L)Fe{=}N\text{-}NH_3\text{---}(EtO)_2]^{1+}$	$^{\mathbf{4}}\mathbf{3}_{\mathbf{P}}^{+}$	-3154.625567	1.149822	-3153.586638	-3155.724279
$^{6}[(L)Fe{=}N\text{-}NH_3\text{---}(EtO)_2]^{1+}$	$^{\mathbf{6}}\mathbf{3}_{\mathbf{P}}^{+}$	-3154.628944	1.150935	-3153.588957	-3155.732060
**Step 3 path 3:**					
$^{2}[(L)Fe{=}N\text{-}NH_2\text{---}(EtO)_2H^+]^{0}$	$^{2}\mathbf{3}_{\mathbf{RC}}^{\mathbf{0}}$	-3154.676123	1.141765	-3153.646139	-3155.778138
$^{4}[(L)Fe{=}N\text{-}NH_2\text{---}(EtO)_2H^+]^{0}$	$^{4}\mathbf{3}_{\mathbf{RC}}^{\mathbf{0}}$	-3154.674925	1.140848	-3153.648036	-3155.775996
$^{6}[(L)Fe{=}N\text{-}NH_2\text{---}(EtO)_2H^+]^{0}$	$^{6}\mathbf{3}_{\mathbf{RC}}^{\mathbf{0}}$	-3154.658957	1.141852	-3153.629222	-3155.756551
$^{2}\mathbf{TS3}_{\mathbf{PT}}$	$^{2}\mathbf{TS3}_{\mathbf{d}}^{\mathbf{0}}$	-3154.668509	1.144440	-3153.631954	-3155.773824
$^{4}\mathbf{TS3}_{\mathbf{PT}}$	$^{4}\mathbf{TS3}_{\mathbf{d}}^{\mathbf{0}}$	-3154.672213	1.143953	-3153.638227	-3155.775820
$^{2}[(L)Fe{=}N\text{-}NH_3\text{---}(EtO)_2]^{0}$	$^{2}\mathbf{3}_{\mathbf{P}}^{\mathbf{0}}$	-3154.720856	1.147617	-3153.682910	-3155.828472

WILEY-VCH

Table S10: Absolute energies, zero-point energies and free energies (in au) of optimized geometries as described in this work.

		E [au]	ZPE [au]	G [au]	E [au]
**Step 5**		THF, BS1	THF, BS1	THF, BS1	THF, BS2
^{2}[(L)Fe-N(H)NH---$(Et_2O)_2H^+$]$^{2+}$	$^{2}\mathbf{3}_{dp,RC}$	-3154.454310	1.149198	-3153.417100	-3155.540246
^{4}[(L)Fe-N(H)NH---$(Et_2O)_2H^+$]$^{2+}$	$^{4}\mathbf{3}_{dp,RC}$	-3154.489360	1.149281	-3153.451061	-3155.577151
^{6}[(L)Fe-N(H)NH---$(Et_2O)_2H^+$]$^{2+}$	$^{6}\mathbf{3}_{dp,RC}$	-3154.470484	1.147571	-3153.435275	-3155.558313
$^{2}\mathbf{TS6}_{PT}$	$^{2}\mathbf{TS3}_{d}$	-3154.424569	1.153273	-3153.375055	
$^{4}\mathbf{TS6}_{PT}$	$^{4}\mathbf{TS3}_{d}$	-3154.477729	1.150156	-3153.437275	-3155.570837
$^{6}\mathbf{TS6}_{PT}$	$^{6}\mathbf{TS3}_{d}$	-3154.460602	1.149522	-3153.420658	-3155.553746
^{2}[(L)Fe-N(H)NH_2---$(Et_2O)_2$]$^{2+}$	$^{2}\mathbf{3}_{dp,P}$	-3154.474156	1.154366	-3153.427737	
^{4}[(L)Fe-N(H)NH_2---$(Et_2O)_2$]$^{2+}$	$^{4}\mathbf{3}_{dp,P}$	-3154.518395	1.152338	-3153.477577	-3155.613798
^{6}[(L)Fe-N(H)NH_2---$(Et_2O)_2$]$^{2+}$	$^{6}\mathbf{3}_{dp,P}$	-3154.507847	1.152246	-3153.467661	-3155.604626
**Step 6**					
^{2}[(L)Fe-N(H)NH---$(EtO)_2H^+$]$^{+}$	$^{2}\mathbf{3}_{dp,RC}^{+}$	-3154.563056	1.148543	-3153.521639	-3155.667147
^{4}[(L)Fe-N(H)NH---$(EtO)_2H^+$]$^{+}$	$^{4}\mathbf{3}_{dp,RC}^{+}$	-3154.621822	1.145868	-3153.586999	-3155.719800
^{6}[(L)Fe-N(H)NH---$(EtO)_2H^+$]$^{+}$	$^{6}\mathbf{3}_{dp,RC}^{+}$	-3154.611644	1.145234	-3153.578810	-3155.707623
$^{2}\mathbf{TS6}_{PT}$	$^{2}\mathbf{TS3}_{d}^{+}$	-3154.550790	1.149549	-3153.506261	-3155.660051
$^{4}\mathbf{TS6}_{PT}$	$^{4}\mathbf{TS3}_{d}^{+}$	-3154.611854	1.147307	-3153.573401	-3155.714554
$^{6}\mathbf{TS6}_{PT}$	$^{6}\mathbf{TS3}_{d}^{+}$	-3154.601395	1.146551	-3153.563418	-3155.703174
^{2}[(L)Fe-N(H)NH_2---$(EtO)_2$]$^{+}$	$^{2}\mathbf{3}_{dp,P}^{+}$	-3154.613172	1.152106	-3153.568782	-3155.720266
^{4}[(L)Fe-N(H)NH_2---$(EtO)_2$]$^{+}$	$^{4}\mathbf{3}_{dp,P}^{+}$	-3154.672449	1.149410	-3153.633129	-3155.772246
^{6}[(L)Fe-N(H)NH_2---$(EtO)_2$]$^{+}$	$^{6}\mathbf{3}_{dp,P}^{+}$	-3154.666880	1.149896	-3153.630192	-3155.768431
**Step 7**					
^{2}[(L)Fe-N(H)NH---$(EtO)_2H^+$]^{0}	$^{2}\mathbf{3}_{dp,RC}^{0}$	-3154.668686	1.144826	-3153.631490	-3155.781405
^{4}[(L)Fe-N(H)NH---$(EtO)_2H^+$]^{0}	$^{4}\mathbf{3}_{dp,RC}^{0}$	-3154.694063	1.142839	-3153.661614	-3155.797380
^{6}[(L)Fe-N(H)NH---$(EtO)_2H^+$]^{0}	$^{6}\mathbf{3}_{dp,RC}^{0}$	-3154.680850	1.142106	-3153.649708	-3155.780795
$^{2}\mathbf{TS6}_{PT}$	$^{2}\mathbf{TS3}_{d}^{0}$	-3154.660126	1.146170	-3153.619614	-3155.801457
$^{4}\mathbf{TS6}_{PT}$	$^{4}\mathbf{TS3}_{d}^{0}$	-3154.685975	1.143623	-3153.650912	-3155.794831
$^{6}\mathbf{TS6}_{PT}$	$^{6}\mathbf{TS3}_{d}^{0}$	-3154.672759	1.142465	-3153.640637	-3155.777744
^{2}[(L)Fe-N(H)NH_2---$(EtO)_2$]^{0}	$^{2}\mathbf{3}_{dp,P}^{0}$	-3154.765939	1.146460	-3153.729942	-3155.876313
^{4}[(L)Fe-N(H)NH_2---$(EtO)_2$]^{0}	$^{4}\mathbf{3}_{dp,P}^{0}$	-3154.782790	1.146896	-3153.748125	-3155.899741
^{6}[(L)Fe-N(H)NH_2---$(EtO)_2$]^{0}	$^{6}\mathbf{3}_{dp,P}^{0}$	-3154.764393	1.146113	-3153.731558	-3155.878767

WILEY-VCH

Table S11: Relative (free) energies (in kcal mol^{-1}) of optimized geometries as described in this work. Spin state ordering and relative energies data for the doubly-reduced system. Optimizations done using default (def) and tight convergence criteria.

	ΔE	ΔE+ZPE	ΔG	ΔE	ΔE+ZPE	ΔG	ΔE	ΔE+ZPE
	BS1,def	BS1,def	BS1,def	BS2	BS2	BS2	BS1,tight	BS1,tight
^{2}**1**$_{RC}$	0.00	0.00	0.00	0.00	0.00	0.00	0.00	0.00
^{4}**1**$_{RC}$	9.36	7.77	6.18	7.23	5.64	4.05	9.36	7.77
^{6}**1**$_{RC}$	23.46	22.20	20.64	30.70	29.43	27.87	23.46	22.20
^{2}**TS1**$_{PT}$	7.35	8.26	9.55	4.02	4.94	6.22	7.35	8.27
^{2}**1**$_{P}$	-18.75	-18.00	-20.03	-24.11	-23.36	-25.40	-18.75	-18.00
^{6}**1**$_{P}$	-1.39	-1.75	-5.48	-7.80	-8.15	-11.89	-1.39	-1.74
^{2}**2**$_{RC}$	0.00	0.00	0.00	0.00	0.00	0.00	0.00	0.00
^{4}**2**$_{RC}$	1.14	0.70	-0.37	-0.17	-0.61	-1.68	1.14	0.69
^{6}**2**$_{RC}$	14.89	13.85	12.12	14.60	13.56	11.83	14.89	13.85
^{2}**TS2**$_{PT}$	6.89	8.33	10.41	4.94	6.39	8.47	6.89	8.33
^{4}**TS2**$_{PT}$	6.50	7.59	9.32	3.28	4.37	6.10		
^{6}**TS2**$_{PT}$	21.17	21.43	21.69	17.67	17.94	18.19	21.17	21.43
^{2}**2**$_{P}$	-21.02	-16.82	-15.33	-19.61	-15.41	-13.92	-21.02	-16.82
^{6}**2**$_{P}$	-19.27	-16.06	-18.07	-17.72	-14.51	-16.53	-19.27	-16.06

WILEY-VCH

Table S12: Relative (free) energies (in kcal mol^{-1}) of optimized geometries as described in this work. Spin state ordering and relative energies data for the doubly-reduced system. Optimizations done using default (def) and tight convergence criteria.

	ΔE	ΔE+ZPE	ΔG	ΔE	ΔE+ZPE	ΔG
	BS1,def	BS1,def	BS1,def	BS2	BS2	BS2
$^{2}\mathbf{3}_{\mathbf{RC}}$	16.86	16.91	15.98	16.83	16.87	15.94
$^{4}\mathbf{3}_{\mathbf{RC}}$	0.00	0.00	0.00	0.00	0.00	0.00
$^{6}\mathbf{3}_{\mathbf{RC}}$	17.52	16.67	20.22	17.98	17.13	20.68
$^{2}\mathbf{TS3}_{\mathbf{PT}}$	32.38	34.63	43.51	30.33	32.58	41.47
$^{4}\mathbf{TS3}_{\mathbf{PT}}$	15.82	17.13	23.24	13.61	14.92	21.03
$^{2}\mathbf{3}_{\mathbf{P}}$	17.22	20.04	26.93	14.18	17.00	23.89
$^{4}\mathbf{3}_{\mathbf{P}}$	6.51	8.81	15.15	1.70	4.00	10.34
$^{6}\mathbf{3}_{\mathbf{P}}$	6.22	9.36	14.96	0.24	3.38	8.98
$^{2}\mathbf{3}_{\mathbf{RC}}^{+}$	0.00	0.00	0.00	0.00	0.00	0.00
$^{4}\mathbf{3}_{\mathbf{RC}}^{+}$	-24.64	-26.88	-29.50			
$^{6}\mathbf{3}_{\mathbf{RC}}^{+}$	-11.71	-14.20	-16.83	-4.87	-7.36	-9.99
$^{2}\mathbf{TS3}_{\mathbf{d}}$	5.09	7.11	11.99	5.03	7.05	11.93
$^{4}\mathbf{TS3}_{\mathbf{d}}$	-14.59	-14.82	-14.28	-11.39	-11.61	-11.07
$^{6}\mathbf{TS3}_{\mathbf{d}}$	0.21	-0.41	0.51	3.26	2.64	3.57
$^{2}\mathbf{3}_{\mathbf{P}}^{+}$	-24.78	-21.11	-18.47	-23.05	-19.38	-16.74
$^{4}\mathbf{3}_{\mathbf{P}}^{+}$	-34.10	-32.99	-33.22	-31.34	-30.22	-30.45
$^{6}\mathbf{3}_{\mathbf{P}}^{+}$	-36.22	-34.41	-34.67	-36.22	-34.41	-34.67
$^{2}\mathbf{3}_{\mathbf{RC}}^{\mathbf{0}}$	0.00	0.00	0.00	0.00	0.00	0.00
$^{4}\mathbf{3}_{\mathbf{RC}}^{\mathbf{0}}$	0.75	0.18	-1.19	1.34	0.77	-0.60
$^{6}\mathbf{3}_{\mathbf{RC}}^{\mathbf{0}}$	10.77	10.83	10.62	13.55	13.60	13.39
$^{2}\mathbf{TS3}_{\mathbf{d}}^{\mathbf{0}}$	4.78	6.46	8.90	2.71	4.39	6.83
$^{4}\mathbf{TS3}_{\mathbf{d}}^{\mathbf{0}}$	2.45	3.83	4.96	1.45	2.83	3.97
$^{2}\mathbf{3}_{\mathbf{P}}^{\mathbf{0}}$	-28.07	-24.40	-23.07	-31.58	-27.91	-26.59

WILEY-VCH

Table S13: Relative (free) energies (in kcal mol^{-1}) of optimized geometries as described in this work. Spin state ordering and relative energies data for the doubly-reduced system. Optimizations done using default (def) and tight convergence criteria.

	ΔE	ΔE+ZPE	ΔG	ΔE	ΔE+ZPE	ΔG
	BS1,def	BS1,def	BS1,def	BS2	BS2	BS2
$^{2}\mathbf{3}_{\mathbf{dp,RC}}$	21.99	21.94	21.31	23.16	23.11	22.48
$^{4}\mathbf{3}_{\mathbf{dp,RC}}$	0.00	0.00	0.00	0.00	0.00	0.00
$^{6}\mathbf{3}_{\mathbf{dp,RC}}$	11.85	10.77	9.91	11.82	10.75	9.88
$^{2}\mathbf{TS3}_{\mathbf{d}}$	40.66	43.16	47.69			
$^{4}\mathbf{TS3}_{\mathbf{d}}$	7.30	7.85	8.65	3.96	4.51	5.31
$^{6}\mathbf{TS3}_{\mathbf{d}}$	18.05	18.20	19.08	14.69	14.84	15.72
$^{2}\mathbf{3}_{\mathbf{dp,P}}$	9.54	12.73	14.64			
$^{4}\mathbf{3}_{\mathbf{dp,P}}$	-18.22	-16.30	-16.64	-23.00	-21.08	-21.42
$^{6}\mathbf{3}_{\mathbf{dp,P}}$	-11.60	-9.74	-10.42	-17.24	-15.38	-16.06
$^{2}\mathbf{3}_{\mathbf{dp,RC}}^{+}$	0.00	0.00	0.00	0.00	0.00	0.00
$^{4}\mathbf{3}_{\mathbf{dp,RC}}^{+}$	-36.88	-38.55	-41.01	-33.04	-34.72	-37.18
$^{6}\mathbf{3}_{\mathbf{dp,RC}}^{+}$	-30.49	-32.57	-35.88	-25.40	-27.48	-30.78
$^{2}\mathbf{TS3}_{\mathbf{d}}^{+}$	7.70	8.33	9.65	4.45	5.08	6.41
$^{4}\mathbf{TS3}_{\mathbf{d}}^{+}$	-30.62	-31.40	-32.48	-29.75	-30.52	-31.61
$^{6}\mathbf{TS3}_{\mathbf{d}}^{+}$	-24.06	-25.31	-26.22	-22.61	-23.86	-24.77
$^{2}\mathbf{3}_{\mathbf{dp,P}}^{+}$	-31.45	-29.21	-29.58	-33.33	-31.10	-31.47
$^{4}\mathbf{3}_{\mathbf{dp,P}}^{+}$	-68.65	-68.10	-69.96	-65.95	-65.41	-67.27
$^{6}\mathbf{3}_{\mathbf{dp,P}}^{+}$	-65.15	-64.30	-68.12	-63.56	-62.71	-66.52
$^{2}\mathbf{3}_{\mathbf{dp,RC}}^{0}$	0.00	0.00	0.00	0.00	0.00	0.00
$^{4}\mathbf{3}_{\mathbf{dp,RC}}^{0}$	-15.92	-17.17	-18.90	-10.02	-11.27	-13.00
$^{6}\mathbf{3}_{\mathbf{dp,RC}}^{0}$	-7.63	-9.34	-11.43	0.38	-1.32	-3.42
$^{2}\mathbf{TS3}_{\mathbf{d}}^{0}$	5.37	6.22	7.45	-12.58	-11.74	-10.50
$^{4}\mathbf{TS3}_{\mathbf{d}}^{0}$	-10.85	-11.60	-12.19	-8.43	-9.18	-9.76
$^{6}\mathbf{TS3}_{\mathbf{d}}^{0}$	-2.56	-4.04	-5.74	2.30	0.82	-0.89
$^{2}\mathbf{3}_{\mathbf{dp,P}}^{0}$	-61.03	-60.00	-61.78	-59.56	-58.53	-60.31
$^{4}\mathbf{3}_{\mathbf{dp,P}}^{0}$	-71.60	-70.30	-73.19	-74.26	-72.96	-75.85
$^{6}\mathbf{3}_{\mathbf{dp,P}}^{0}$	-60.06	-59.25	-62.79	-61.10	-60.29	-63.83

WILEY-VCH

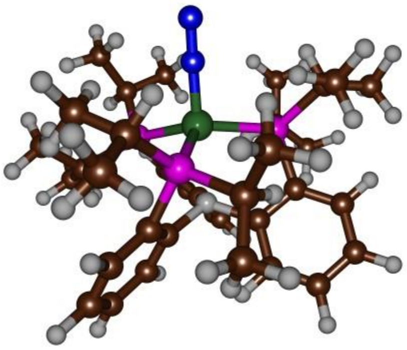

rNN = 1.183 (1.158) [2.205]
rNFe = 1.805 (1.906) [2.172]
aFeNN = 179 (180) [137]

$^{2}\mathbf{1}$ ($^{4}\mathbf{1}$) [$^{6}\mathbf{1}$]

Figure S1: UB3LYP/BS1 optimized geometries of $^{2,4,6}\mathbf{1}$ in Gaussian with bond lengths in angstroms and angles in degrees.

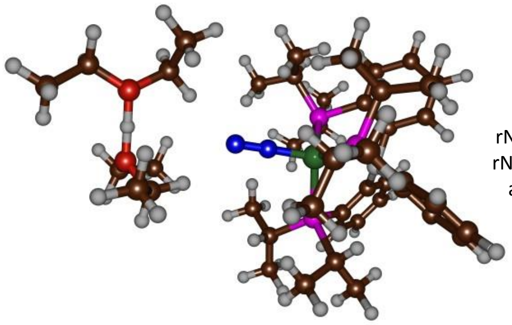

rNN = 1.193 (1.163) [2.208]
rNFe = 1.804 (1.891) [2.011]
aFeNN = 179 (180) [171]

$^{2}\mathbf{1}_{RC}$ ($^{4}\mathbf{1}_{RC}$) [$^{6}\mathbf{1}_{RC}$]

Figure S2: UB3LYP/BS1 optimized geometries of $^{2,4,6}\mathbf{1}_{RC}$ in Gaussian with bond lengths in angstroms and angles in degrees.

WILEY-VCH

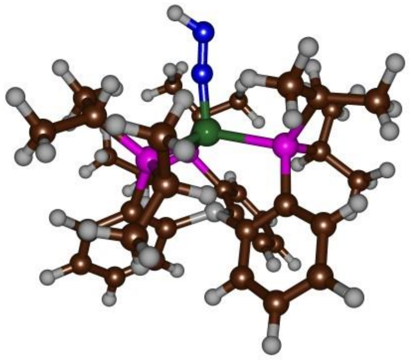

$^{2}\mathbf{2_d}$ ($^{4}\mathbf{2_d}$) [$^{6}\mathbf{2_d}$]

Figure S3: UB3LYP/BS1 optimized geometries of $^{2,4,6}\mathbf{2_d}$ in Gaussian with bond lengths in angstroms and angles in degrees.

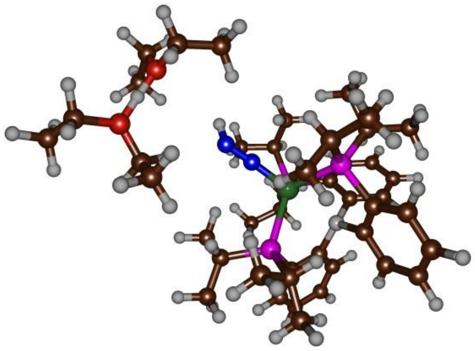

$^{2}\mathbf{2_{d,RC}}$ ($^{4}\mathbf{2_{d,RC}}$) [$^{6}\mathbf{2_{d,RC}}$]

Figure S4: UB3LYP/BS1 optimized geometries of $^{2,4,6}\mathbf{2_{d,RC}}$ in Gaussian with bond lengths in angstroms and angles in degrees.

WILEY-VCH

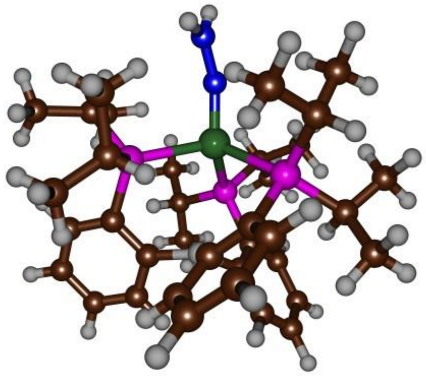

rHN = 1.013 (1.017) [1.013]
rHN = 1.014 (1.020) [1.014]
rNN = 1.323 (1.298) [1.348]
rNFe = 1.718 (1.819) [1.890]
aFeNN = 156 (148) [147]

$^{2}\mathbf{3}_{dd}$ ($^{4}\mathbf{3}_{dd}$) [$^{6}\mathbf{3}_{dd}$]

Figure S5: UB3LYP/BS1 optimized geometries of ^{2,4,6}3$_{dd}$ in Gaussian with bond lengths in angstroms and angles in degrees.

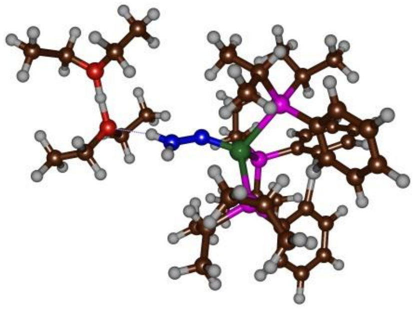

rHN = 3.747 (3.503) [3.518]
rHN = 1.016 (1.019) [1.016]
rHN = 1.014 (1.020) [1.016]
rNN = 1.331 (1.303) [1.352]
rNFe = 1.744 (1.828) [1.907]
aFeNN = 149 (145) [144]

$^{2}\mathbf{3}_{dd,RC}$ ($^{4}\mathbf{3}_{dd,RC}$) [$^{6}\mathbf{3}_{dd,RC}$]

Figure S6: UB3LYP/BS1 optimized geometries of ^{2,4,6}3$_{dd,RC}$ in Gaussian with bond lengths in angstroms and angles in degrees.

WILEY-VCH

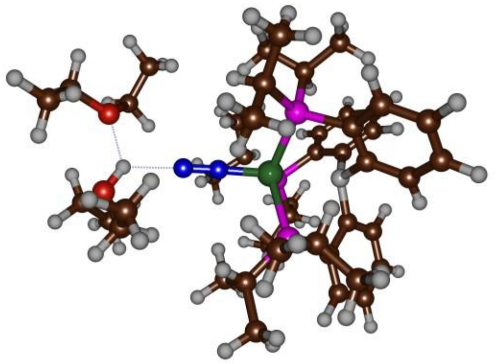

^{2}**TS1$_{d}$** [^{6}**TS1$_{d}$**]

Figure S7: UB3LYP/BS1 optimized geometries of ^{2,6}TS1$_{d}$ in Gaussian with bond lengths in angstroms, angles in degrees and the imaginary frequency in cm^{-1}.

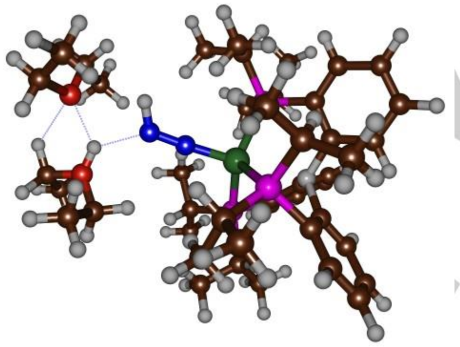

^{2}**TS2$_{d}$** (^{4}**TS2$_{d}$**) [^{6}**TS2$_{d}$**]

Figure S8: UB3LYP/BS1 optimized geometries of ^{2,4,6}TS2$_{d}$ in Gaussian with bond lengths in angstroms, angles in degrees and the imaginary frequency in cm^{-1}.

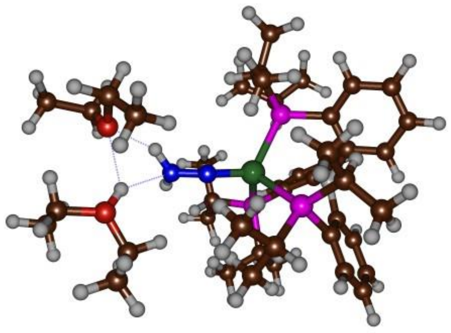

r_{FeN}: 1.770 (1.819)
r_{NN}: 1.353 (1.325)
r_{NH}: 1.019 (1.022)
r_{NH}: 1.030 (1.027)
r_{NH}: 2.106 (2.043)
∠FeNN: 146 (148) °

i137 (i188) cm^{-1}

^{2}**TS3**$_{\mathbf{d}}$ (^{4}**TS3**$_{\mathbf{d}}$)

Figure S9: UB3LYP/BS1 optimized geometries of ^{2,4}TS3$_{d}$ in Gaussian with bond lengths in angstroms, angles in degrees and the imaginary frequency in cm^{-1}.

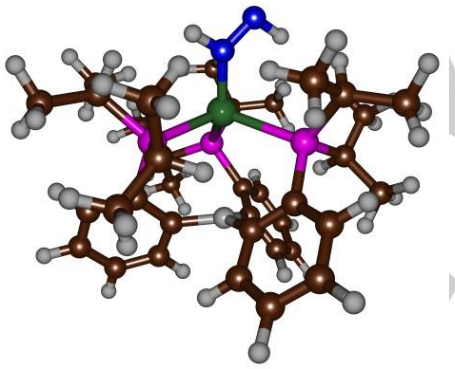

rHN = 1.036 (1.038) [1.035]
rHN = 1.020 (1.032) [1.021]
rNN = 1.348 (1.323) [1.381]
rNFe = 1.848 (1.933) [1.959]
aFeNN = 126 (126) [103]

^{2}**3**$_{\mathbf{dp}}$ (^{4}**3**$_{\mathbf{dp}}$) [^{6}**3**$_{\mathbf{dp}}$]

Figure S10: UB3LYP/BS1 optimized geometries of ^{2,4,6}3$_{dp}$ in Gaussian with bond lengths in angstroms and angles in degrees.

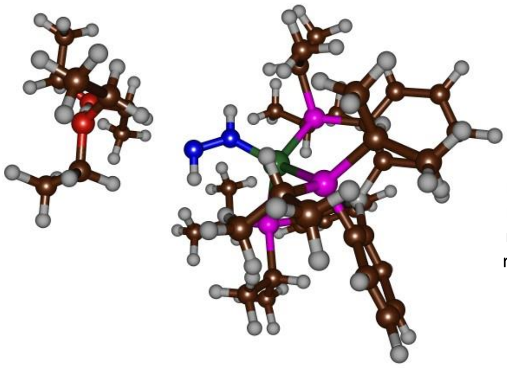

rHN = 1.035 (1.034) [1.036]
rHN = 1.019 (1.026) [1.018]
rNN = 1.351 (1.328) [1.386]
rNFe = 1.858 (1.944) [1.984]
aFeNN = 126 (135) [124]

^{2}**3**$_{dp,RC}$ (^{4}**3**$_{dp,RC}$) [^{6}**3**$_{dp,RC}$]

Figure S11: UB3LYP/BS1 optimized geometries of ^{2,4,6}3$_{dp,RC}$ in Gaussian with bond lengths in angstroms and angles in degrees.

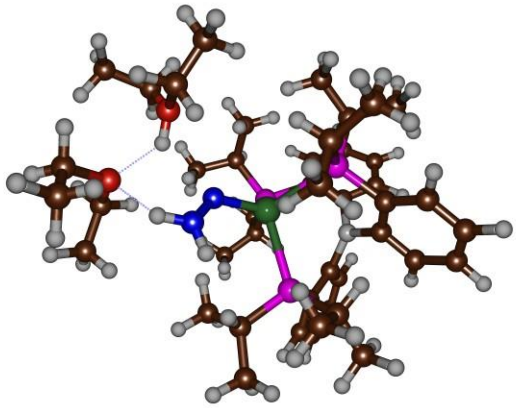

r_{FeN}: (1.875) [1.966]
r_{NN}: (1.319) [1.360]
r_{NH}: (2.235) [2.285]
∠FeNN: (132) [127]°

(i211) [i487] cm^{-1}

(^{4}**TS3**$_{p}$) [^{6}**TS3**$_{p}$]

Figure S12: UB3LYP/BS1 optimized geometries of ^{2,4,6}TS3$_{p}$ in Gaussian with bond lengths in angstroms, angles in degrees and the imaginary frequency in cm^{-1}.

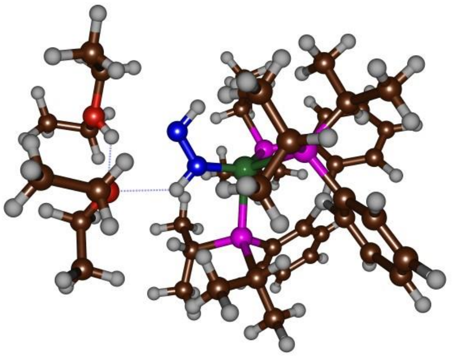

r_{FeN}: 1.849 (1.941) [1.998]
r_{NN}: 1.309 (1.331) [1.383]
r_{NH}: 1.028 (1.026) [1.018]
r_{NH}: 1.035 (1.031) [1.032]
r_{NH}: 2.133 (2.048) [2.074]
∠FeNN: 125 (137) [126]°
i247 (i285) [i320] cm^{-1}

^{2}**TS3'$_{d}$** (^{4}**TS3'$_{d}$**) [^{6}**TS3'$_{d}$**]

Figure S13: UB3LYP/BS1 optimized geometries of ^{2,4,6}TS3'$_{d}$ in Gaussian with bond lengths in angstroms, angles in degrees and the imaginary frequency in cm^{-1}.

WILEY-VCH

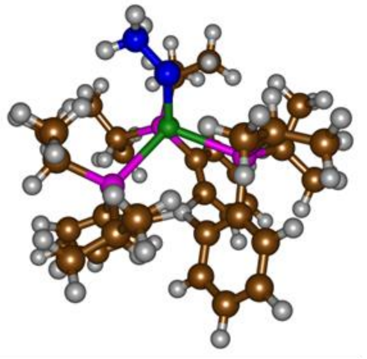

r_{FeN}: 1.548 (1.812) [1.839]
r_{NN}: 3.287 (1.568) [1.505]
r_{NH}: 1.011 (1.023) [1.026]
r_{NH}: 1.011 (1.023) [1.024]
r_{NH}: 1.013 (1.024) [1.027]
∠FeNN = 135 (124) [128]

$^{2}\mathbf{4}_{ddd}$ ($^{4}\mathbf{4}_{ddd}$) [$^{6}\mathbf{4}_{ddd}$]

Figure S14: UB3LYP/BS1 optimized geometries of $^{2,4,6}\mathbf{4}_{ddd}$ in Gaussian with bond lengths in angstroms and angles in degrees.

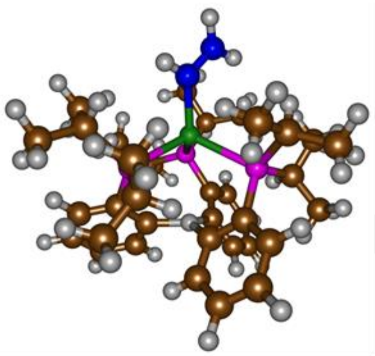

r_{FeN}: 1.937 (2.004) [2.111]
r_{NN}: 1.335 (1.333) [1.344]
r_{NH}: 1.021 (1.023) [1.021]
r_{NH}: 1.013 (1.013) [1.012]
r_{NH}: 1.010 (1.010) [1.019]
∠FeNN = 127 (131) [124]

$^{2}\mathbf{4}_{dpd}$ ($^{4}\mathbf{4}_{dpd}$) [$^{6}\mathbf{4}_{dpd}$]

Figure S15: UB3LYP/BS1 optimized geometries of $^{2,4,6}\mathbf{4}_{dpd}$ in Gaussian with bond lengths in angstroms and angles in degrees.

WILEY-VCH

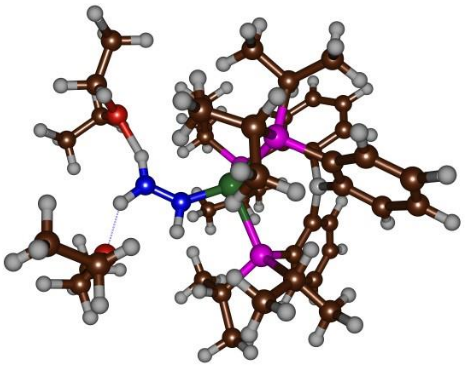

r_{FeN}: 1.840 (1.983 [2.076]
r_{NN}: 1.316 (1.331) [1.345]
r_{NH}: 1.028 (1.024) [1.021]
r_{NH}: 1.031 (1.011) [1.010]
r_{NH}: 1.037 (1.049) [1.043]
∠FeNN: 136 (131) [127]°

$^{2}\mathbf{4}_{dpd,RC}$ ($^{4}\mathbf{4}_{dpd,RC}$) [$^{6}\mathbf{4}_{dpd,RC}$]

Figure S16: UB3LYP/BS1 optimized geometries of $^{2,4,6}\mathbf{4}_{dpd,RC}$ in Gaussian with bond lengths in angstroms and angles in degrees.

WILEY-VCH

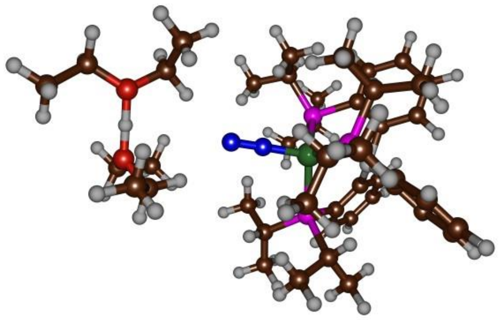

r_{NN} = 1.191
r_{NFe} = 1.794
a_{FeNN} = 178
r_{FeB} = 2.410

$^{2}\mathbf{1}_{RC}$

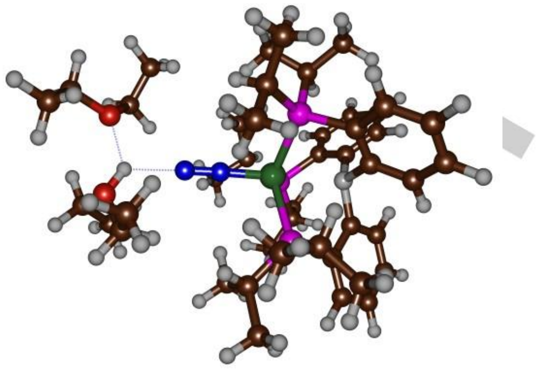

r_{FeN}: 1.793
r_{NN}: 1.200
r_{NH}: 1.956
∠FeNN: 179°

i281 cm^{-1}

ΔE+ZPE$_{BS1}$ = 7.3

$^{2}\mathbf{TS1}_{d}$

Figure S17: UPBE0/BS1 optimized geometries of the first proton transfer step from ^{2}1 with bond lengths in angstroms and angles in degrees.

WILEY-VCH

Cartesian coordinates:

$(Et_2O)_2H^+$:

1	-1.628178000	4.092764000	-0.346024000
8	-1.700304000	0.673593000	-0.487123000
6	-0.869015000	0.076051000	-1.567252000
6	-1.736167000	-0.507892000	-2.668381000
1	-0.200513000	0.861131000	-1.928227000
1	-0.275737000	-0.686495000	-1.059188000
1	-1.092103000	-0.954719000	-3.433890000
1	-2.395747000	-1.288075000	-2.276841000
1	-2.347682000	0.259301000	-3.153570000
6	-2.229701000	2.055695000	-0.640998000
6	-1.196800000	3.091944000	-0.233454000
1	-2.543855000	2.159569000	-1.681917000
1	-3.114695000	2.078451000	-0.002402000
1	-0.901075000	2.962727000	0.812132000
1	-0.301487000	3.041756000	-0.861065000
1	-1.796080000	-3.250078000	1.983144000
8	-2.041570000	-0.520967000	1.546010000
6	-1.140095000	-0.300739000	2.709205000
6	-1.775314000	0.629428000	3.727369000
1	-0.908010000	-1.284782000	3.123178000
1	-0.232985000	0.120934000	2.271965000
1	-2.002328000	1.602601000	3.281939000
1	-2.696677000	0.208940000	4.142423000
1	-1.076168000	0.785800000	4.556304000
6	-3.110716000	-1.553027000	1.624348000
6	-2.572868000	-2.931217000	1.281113000
1	-3.525650000	-1.500943000	2.633407000
1	-3.866862000	-1.213764000	0.913922000
1	-3.390039000	-3.659218000	1.332855000
1	-2.160734000	-2.951112000	0.267614000
1	-1.869214000	0.078907000	0.532858000

NH_3:

7	-0.960147000	0.000017000	-1.040432000
1	-0.960171000	0.969829000	-0.747831000
1	-0.120272000	-0.484925000	-0.747830000
1	-1.800065000	-0.484921000	-0.747945000

^{2}**1**:

26	-0.022621000	-0.187956000	-1.013756000
15	-0.526839000	2.187469000	-0.879974000
15	2.280851000	-0.757025000	-0.413456000
15	-1.962045000	-1.556613000	-0.222992000
7	0.023623000	-0.514190000	-2.788423000
6	-1.179007000	-2.169377000	1.389230000
6	2.523637000	-2.595344000	0.210782000
1	1.547847000	-2.795689000	0.665464000
6	-0.257206000	-1.258978000	1.981859000
6	2.656901000	0.344022000	1.074859000
6	-1.555935000	3.063004000	-2.303726000
1	-0.849738000	2.963438000	-3.139832000
6	-1.174640000	1.272931000	1.658519000
6	1.674447000	1.702712000	2.809696000
1	0.802850000	2.070231000	3.345395000
6	-2.340973000	-3.195495000	-1.205843000
1	-3.061811000	-3.782048000	-0.620654000
5	0.012409000	0.210365000	1.349412000
6	0.386268000	-1.707995000	3.165887000
1	1.099481000	-1.047303000	3.651486000
6	0.138977000	-2.965857000	3.729182000
1	0.658813000	-3.267301000	4.635831000
6	3.934009000	0.762822000	1.480990000
1	4.819702000	0.394923000	0.977080000
6	-0.779071000	-3.835473000	3.126825000
1	-0.976357000	-4.815494000	3.552372000
6	4.084684000	1.672182000	2.538619000
1	5.075835000	2.004208000	2.835205000
6	2.945354000	2.146904000	3.198948000
1	3.046823000	2.854795000	4.018479000
6	3.689233000	-0.534661000	-1.751222000
1	3.323593000	-1.208005000	-2.538968000
6	-1.913964000	1.248856000	2.867804000
1	-1.710896000	0.457670000	3.585796000
6	-1.484607000	2.323393000	0.747664000
6	0.991072000	3.416996000	-0.656678000
1	1.735138000	2.743621000	-0.218172000
6	-3.733555000	-0.996384000	0.430703000
1	-3.450773000	-0.152643000	1.070241000
6	-2.448485000	3.295905000	1.057553000
1	-2.655696000	4.103881000	0.366847000
6	-2.896344000	2.202708000	3.168343000
1	-3.445910000	2.142927000	4.105098000
6	-2.949877000	-2.871177000	-2.584340000
1	-3.107802000	-3.802484000	-3.145998000
1	-2.268089000	-2.239790000	-3.164707000
1	-3.912974000	-2.360827000	-2.513866000
6	-3.164328000	3.236005000	2.262939000
1	-3.919544000	3.984924000	2.485851000
6	-1.054864000	-4.017804000	-1.395852000
1	-1.280436000	-4.941579000	-1.946584000
1	-0.588494000	-4.293770000	-0.446183000
1	-0.326154000	-3.446500000	-1.981148000
6	-1.930835000	4.553563000	-2.183303000
1	-2.235931000	4.930647000	-3.170361000
1	-1.108181000	5.179705000	-1.832362000
1	-2.781878000	4.702542000	-1.511054000
6	3.649075000	0.898876000	-2.303298000
1	4.350460000	1.000854000	-3.142743000
1	3.935427000	1.628193000	-1.536064000
1	2.648013000	1.153230000	-2.659126000
6	-1.439635000	-3.426649000	1.959393000
1	-2.147741000	-4.104855000	1.491813000
6	1.533568000	3.907687000	-2.012020000
1	2.531129000	4.347036000	-1.874291000
1	0.896180000	4.680161000	-2.454373000
1	1.628205000	3.092880000	-2.736745000
6	-2.803422000	2.233150000	-2.644919000
1	-3.276003000	2.617078000	-3.559772000
1	-3.544008000	2.293272000	-1.838715000
1	-2.551689000	1.180727000	-2.802657000
6	2.711782000	-3.562101000	-0.972704000

1	2.620849000	-4.598857000	-0.621440000
1	3.699301000	-3.459280000	-1.435088000
1	1.956742000	-3.407169000	-1.750144000
6	0.777616000	4.581371000	0.324431000
1	1.716923000	5.141971000	0.440023000
1	0.478354000	4.220830000	1.312202000
1	0.014396000	5.287202000	-0.022215000
6	3.593374000	-2.792969000	1.296127000
1	3.558670000	-3.829793000	1.660072000
1	3.421586000	-2.130996000	2.149188000
1	4.606456000	-2.608535000	0.923461000
6	-4.656701000	-0.475038000	-0.684539000
1	-5.462502000	0.128565000	-0.244418000
1	-5.132468000	-1.298920000	-1.227766000
1	-4.125407000	0.148337000	-1.406883000
6	5.135215000	-0.945865000	-1.416085000
1	5.733699000	-0.933432000	-2.338430000
1	5.206960000	-1.949848000	-0.992534000
1	5.608700000	-0.247173000	-0.719443000
6	-4.464393000	-2.038890000	1.290461000
1	-5.399578000	-1.607649000	1.675672000
1	-3.866243000	-2.359274000	2.147347000
1	-4.733870000	-2.928783000	0.707447000
6	1.479743000	0.788186000	1.743992000
7	0.080175000	-0.719135000	-3.952159000

^{4}**1**:

26	-0.094218000	-0.102248000	-0.973156000
15	-0.482333000	2.516159000	-0.860240000
15	2.353002000	-0.871038000	-0.484626000
15	-2.011687000	-1.847848000	-0.226450000
7	-0.178206000	-0.345734000	-2.861325000
6	-1.182158000	-2.234609000	1.430221000
6	2.556679000	-2.679765000	0.204631000
1	1.578102000	-2.846016000	0.667067000
6	-0.255022000	-1.258126000	1.936770000
6	2.614297000	0.321931000	0.951924000
6	-1.525184000	3.505758000	-2.201557000
1	-0.869974000	3.388439000	-3.077046000
6	-1.253430000	1.233733000	1.545592000
6	1.619199000	1.870016000	2.518863000
1	0.742651000	2.289986000	3.005527000
6	-2.384369000	-3.614585000	-0.974284000
1	-2.963304000	-4.189703000	-0.240725000
5	-0.048395000	0.206915000	1.296024000
6	0.464606000	-1.665776000	3.101241000
1	1.187154000	-0.969783000	3.519685000
6	0.269215000	-2.892307000	3.739924000
1	0.840495000	-3.137191000	4.633395000
6	3.887063000	0.793634000	1.327383000
1	4.777965000	0.378094000	0.870573000
6	-0.678595000	-3.801296000	3.242327000
1	-0.849469000	-4.755117000	3.734013000
6	4.032912000	1.809537000	2.279257000
1	5.022030000	2.165931000	2.553891000
6	2.880222000	2.357617000	2.867356000
1	2.972349000	3.148126000	3.609720000
6	3.818468000	-0.661721000	-1.775676000
1	3.467643000	-1.332761000	-2.573204000
6	-2.169090000	1.073972000	2.632011000
1	-2.055981000	0.194246000	3.261048000
6	-1.468127000	2.417400000	0.745421000
6	1.069666000	3.647091000	-0.531809000
1	1.747966000	2.946329000	-0.034931000
6	-3.766127000	-1.188735000	0.325622000
1	-3.485340000	-0.441758000	1.076079000
6	-2.448806000	3.366639000	1.088740000
1	-2.564693000	4.265316000	0.493932000
6	-3.163869000	2.001275000	2.946549000
1	-3.817374000	1.828700000	3.799746000
6	-3.207887000	-3.492524000	-2.268919000
1	-3.302343000	-4.477313000	-2.746410000
1	-2.720875000	-2.819945000	-2.986279000
1	-4.218658000	-3.117547000	-2.085216000
6	-3.302130000	3.172937000	2.182196000
1	-4.057683000	3.914133000	2.427866000
6	-1.055858000	-4.339519000	-1.247551000
1	-1.247462000	-5.336180000	-1.668078000
1	-0.461361000	-4.463376000	-0.337591000
1	-0.452261000	-3.780505000	-1.973240000
6	-1.800448000	5.011919000	-2.033482000
1	-2.248180000	5.401541000	-2.959644000
1	-0.891837000	5.587374000	-1.841745000
1	-2.509389000	5.217295000	-1.225640000
6	3.786300000	0.775792000	-2.323592000
1	4.475516000	0.875107000	-3.173166000
1	4.090640000	1.498067000	-1.557373000
1	2.781873000	1.047351000	-2.665728000
6	-1.394995000	-3.460500000	2.088142000
1	-2.106604000	-4.177594000	1.688423000
6	1.719194000	4.095954000	-1.853494000
1	2.689890000	4.567048000	-1.647294000
1	1.106347000	4.831166000	-2.386476000
1	1.896172000	3.250805000	-2.527590000
6	-2.827953000	2.738153000	-2.485757000
1	-3.350077000	3.180718000	-3.344983000
1	-3.505242000	2.777670000	-1.624900000
1	-2.628355000	1.685961000	-2.714398000
6	2.731128000	-3.685605000	-0.947597000
1	2.646857000	-4.710997000	-0.563178000
1	3.712470000	-3.594917000	-1.426281000
1	1.964706000	-3.555176000	-1.719476000
6	0.826946000	4.821550000	0.429042000
1	1.782320000	5.314024000	0.659656000
1	0.389702000	4.474983000	1.369548000
1	0.159217000	5.577557000	0.002750000
6	3.625103000	-2.848405000	1.295768000
1	3.582363000	-3.870129000	1.699326000
1	3.457087000	-2.152158000	2.122077000
1	4.638556000	-2.685638000	0.914464000
6	-4.507787000	-0.462706000	-0.809767000
1	-5.398185000	0.040813000	-0.407878000
1	-4.846322000	-1.152060000	-1.590624000
1	-3.876469000	0.296511000	-1.279723000

6	5.254916000	-1.085194000	-1.416185000
1	5.884044000	-1.026566000	-2.316627000
1	5.311265000	-2.111536000	-1.044686000
1	5.706781000	-0.427450000	-0.667486000
6	-4.649584000	-2.249741000	0.999656000
1	-5.557241000	-1.777222000	1.400777000
1	-4.132955000	-2.738343000	1.832275000
1	-4.970899000	-3.024335000	0.293003000
6	1.414591000	0.822201000	1.571997000
7	-0.226250000	-0.484199000	-4.009847000

^{6}**1**:

26	0.018732000	-0.218885000	-0.991294000
15	-0.401212000	2.353129000	-0.981840000
15	2.568078000	-0.745529000	-0.456289000
15	-2.310385000	-1.648495000	-0.111131000
7	0.093905000	-0.565997000	-3.133875000
6	-1.292026000	-2.182102000	1.405696000
6	2.720369000	-2.583402000	0.160047000
1	1.737724000	-2.732543000	0.620698000
6	-0.251214000	-1.302873000	1.866521000
6	2.723866000	0.356250000	1.066516000
6	-1.493729000	3.127996000	-2.406050000
1	-0.815327000	3.007450000	-3.262304000
6	-1.161594000	1.227989000	1.487153000
6	1.608009000	1.750299000	2.691818000
1	0.697763000	2.107932000	3.165894000
6	-2.646969000	-3.332699000	-1.024865000
1	-3.166791000	-4.007052000	-0.332919000
5	0.039828000	0.164249000	1.265127000
6	0.526443000	-1.807247000	2.953502000
1	1.321959000	-1.179680000	3.344384000
6	0.307943000	-3.053711000	3.544743000
1	0.932702000	-3.376801000	4.374884000
6	3.960954000	0.841858000	1.529643000
1	4.884598000	0.493939000	1.081992000
6	-0.722193000	-3.881318000	3.077638000
1	-0.905791000	-4.853591000	3.526133000
6	4.027870000	1.789519000	2.558426000
1	4.990895000	2.159601000	2.899117000
6	2.836397000	2.253327000	3.132552000
1	2.867089000	2.993149000	3.929398000
6	4.114820000	-0.458306000	-1.627080000
1	3.800361000	-1.036492000	-2.507663000
6	-1.998574000	1.153713000	2.634637000
1	-1.861241000	0.316453000	3.313940000
6	-1.390559000	2.350523000	0.627892000
6	1.072987000	3.603050000	-0.723435000
1	1.803236000	2.968780000	-0.208524000
6	-4.035859000	-1.190147000	0.704679000
1	-3.717770000	-0.589540000	1.564477000
6	-2.342953000	3.334838000	0.947463000
1	-2.476279000	4.194652000	0.302248000
6	-2.965705000	2.118107000	2.938123000
1	-3.572565000	2.016770000	3.835061000
6	-3.541279000	-3.095359000	-2.256098000
1	-3.694398000	-4.041696000	-2.792999000
1	-3.065807000	-2.389803000	-2.948385000
1	-4.527809000	-2.705184000	-1.986657000
6	-3.136523000	3.225627000	2.096414000
1	-3.872709000	3.990524000	2.326915000
6	-1.311242000	-3.972249000	-1.444514000
1	-1.499085000	-4.914222000	-1.978321000
1	-0.675636000	-4.192970000	-0.581775000
1	-0.760870000	-3.307267000	-2.120156000
6	-1.911148000	4.608874000	-2.343852000
1	-2.337069000	4.902483000	-3.313970000
1	-1.074104000	5.280048000	-2.136859000
1	-2.684831000	4.783807000	-1.590001000
6	4.157448000	1.024798000	-2.035600000
1	4.904514000	1.177499000	-2.825839000
1	4.426665000	1.666044000	-1.188809000
1	3.186961000	1.357313000	-2.417561000
6	-1.512186000	-3.432383000	2.009992000
1	-2.292580000	-4.087773000	1.634418000
6	1.672732000	4.030311000	-2.075718000
1	2.633302000	4.536184000	-1.911517000
1	1.020823000	4.730964000	-2.608519000
1	1.855672000	3.173328000	-2.733063000
6	-2.718414000	2.225850000	-2.641289000
1	-3.247388000	2.539918000	-3.551273000
1	-3.423440000	2.297448000	-1.805033000
1	-2.432043000	1.176127000	-2.758231000
6	2.841703000	-3.539259000	-1.040971000
1	2.766466000	-4.580001000	-0.698518000
1	3.802864000	-3.432739000	-1.556942000
1	2.043708000	-3.369673000	-1.772962000
6	0.773452000	4.807662000	0.182531000
1	1.698538000	5.374769000	0.358027000
1	0.383958000	4.488076000	1.153140000
1	0.046386000	5.493650000	-0.265884000
6	3.783877000	-2.845673000	1.236951000
1	3.669254000	-3.866758000	1.627561000
1	3.675939000	-2.151187000	2.075473000
1	4.802868000	-2.754115000	0.848170000
6	-4.863786000	-0.285532000	-0.224755000
1	-5.763556000	0.066005000	0.298961000
1	-5.193462000	-0.814103000	-1.126680000
1	-4.290669000	0.591660000	-0.539238000
6	5.508863000	-0.968340000	-1.217859000
1	6.215406000	-0.778827000	-2.039064000
1	5.517099000	-2.043554000	-1.022745000
1	5.901071000	-0.459889000	-0.331849000
6	-4.854587000	-2.385995000	1.213166000
1	-5.750321000	-2.025041000	1.738368000
1	-4.284774000	-3.001912000	1.915679000
1	-5.195902000	-3.027088000	0.391476000
6	1.488371000	0.775526000	1.662567000
7	-0.625493000	-1.033088000	-3.980870000

^{2}**1**$_{RC}$:

26	0.252021000	-0.372546000	-1.076131000
15	0.205202000	2.079247000	-0.950743000
15	2.078028000	-1.387000000	0.226102000

15	-2.091842000	-1.336999000	-0.965300000	1	3.466157000	0.291224000	-1.799222000
7	0.763834000	-0.705306000	-2.774053000	6	-2.593153000	-3.262202000	1.169991000
6	-1.959317000	-2.072982000	0.775197000	1	-3.203837000	-3.819912000	0.465929000
6	1.753445000	-3.216634000	0.835662000	6	2.824864000	3.336425000	-1.144759000
1	0.663739000	-3.220235000	0.938100000	1	3.746872000	3.619616000	-0.618691000
6	-1.134531000	-1.334227000	1.672516000	1	2.547809000	4.176777000	-1.790041000
6	2.162674000	-0.333083000	1.792091000	1	3.060384000	2.480876000	-1.785290000
6	-0.054835000	3.102504000	-2.603668000	6	-1.202747000	2.506661000	-3.433949000
1	0.887049000	2.862538000	-3.117350000	1	-1.178797000	2.912257000	-4.455242000
6	-1.422833000	1.319153000	1.168647000	1	-2.174815000	2.767650000	-3.000868000
6	0.962398000	1.235572000	3.179905000	1	-1.132451000	1.416931000	-3.491014000
1	0.052694000	1.776005000	3.427924000	6	2.136069000	-4.241910000	-0.247539000
6	-2.412590000	-2.891730000	-2.085918000	1	1.744533000	-5.230598000	0.027334000
1	-3.295990000	-3.410482000	-1.693383000	1	3.221348000	-4.339828000	-0.356974000
5	-0.415408000	0.052014000	1.239235000	1	1.717838000	-3.979107000	-1.224907000
6	-0.996667000	-1.873775000	2.978315000	6	1.371673000	4.225724000	0.748299000
1	-0.376047000	-1.344358000	3.696124000	1	2.277266000	4.589945000	1.254613000
6	-1.626098000	-3.060367000	3.374879000	1	0.637238000	3.968759000	1.516038000
1	-1.489902000	-3.435714000	4.386355000	1	0.967865000	5.056795000	0.159662000
6	3.305000000	-0.148147000	2.586668000	6	2.351686000	-3.570215000	2.206398000
1	4.217735000	-0.689719000	2.370731000	1	2.017295000	-4.574167000	2.504028000
6	-2.427611000	-3.765120000	2.468716000	1	2.025140000	-2.865317000	2.975841000
1	-2.912555000	-4.691958000	2.761862000	1	3.446746000	-3.576843000	2.196115000
6	3.287142000	0.746769000	3.666834000	6	-4.110037000	0.528324000	-1.956354000
1	4.181037000	0.895100000	4.266137000	1	-5.055310000	1.057452000	-1.771969000
6	2.109384000	1.445121000	3.957069000	1	-4.182904000	0.062503000	-2.945505000
1	2.082368000	2.144765000	4.789065000	1	-3.309130000	1.269171000	-1.987374000
6	3.848126000	-1.487348000	-0.598175000	6	5.018879000	-2.124830000	0.173128000
1	3.627435000	-2.130655000	-1.461615000	1	5.856551000	-2.288857000	-0.519860000
6	-2.518818000	1.447433000	2.058506000	1	4.765348000	-3.090797000	0.615068000
1	-2.703065000	0.650417000	2.774926000	1	5.388643000	-1.471523000	0.969339000
6	-1.225688000	2.389659000	0.248663000	6	-5.033911000	-1.498140000	-0.733382000
6	1.724867000	3.006913000	-0.119430000	1	-5.965099000	-0.950340000	-0.531557000
1	2.101803000	2.224122000	0.546220000	1	-4.889706000	-2.213446000	0.082053000
6	-3.868572000	-0.503583000	-0.844145000	1	-5.183251000	-2.059252000	-1.663321000
1	-3.780545000	0.038146000	0.104003000	6	0.941163000	0.343817000	2.078598000
6	-2.068626000	3.512153000	0.245712000	7	1.122718000	-0.910718000	-3.893152000
1	-1.898804000	4.325856000	-0.448686000	1	5.070747000	0.151044000	-5.478095000
6	-3.373672000	2.558097000	2.044752000	8	2.332764000	-0.405801000	-7.413854000
1	-4.207356000	2.612639000	2.740820000	6	1.232639000	0.348159000	-8.077528000
6	-2.693749000	-2.464009000	-3.537993000	6	0.051083000	0.604346000	-7.157537000
1	-2.803561000	-3.353758000	-4.173275000	1	1.687397000	1.273379000	-8.440300000
1	-1.865240000	-1.866890000	-3.935459000	1	0.963076000	-0.273069000	-8.933827000
1	-3.613924000	-1.879192000	-3.630091000	1	-0.717354000	1.151381000	-7.715493000
6	-3.150123000	3.598058000	1.135255000	1	-0.386934000	-0.331039000	-6.798215000
1	-3.804730000	4.464980000	1.114357000	1	0.333652000	1.207407000	-6.290377000
6	-1.207759000	-3.844083000	-2.025800000	6	3.110900000	0.279608000	-6.328375000
1	-1.394351000	-4.725726000	-2.654645000	6	4.493003000	-0.343557000	-6.266622000
1	-1.011767000	-4.190739000	-1.006635000	1	3.148376000	1.332732000	-6.617152000
1	-0.306283000	-3.343371000	-2.395368000	1	2.546244000	0.150760000	-5.400811000
6	-0.178449000	4.637547000	-2.551757000	1	4.438541000	-1.409278000	-6.024340000
1	-0.074218000	5.042367000	-3.569007000	1	5.026909000	-0.222280000	-7.213824000
1	0.584819000	5.110980000	-1.930951000	1	3.043264000	-4.036149000	-4.990405000
1	-1.159824000	4.953443000	-2.184172000	8	2.200263000	-2.800403000	-7.273947000
6	4.240321000	-0.101901000	-1.135036000	6	2.370450000	-3.595287000	-8.519034000
1	5.181623000	-0.166260000	-1.697937000	6	3.515776000	-3.013047000	-9.328124000
1	4.387489000	0.613018000	-0.316975000	1	2.581479000	-4.619961000	-8.208134000

1	1.418749000	-3.570901000	-9.059935000
1	3.320649000	-1.977403000	-9.621836000
1	4.450551000	-3.047550000	-8.761167000
1	3.645058000	-3.604120000	-10.240906000
6	1.360603000	-3.390439000	-6.185702000
6	2.129403000	-4.454738000	-5.422658000
1	1.115739000	-2.541668000	-5.540838000
1	0.451967000	-3.780531000	-6.655373000
1	1.501832000	-4.822796000	-4.602977000
1	2.394864000	-5.313051000	-6.047892000
1	2.246612000	-1.581571000	-7.347497000

$^{4}\mathbf{1}_{RC}$:

26	0.395235000	-0.216055000	-0.987983000
15	0.405166000	2.414555000	-0.794160000
15	2.315402000	-1.366762000	0.359189000
15	-1.956179000	-1.590309000	-1.209853000
7	1.000129000	-0.433403000	-2.766380000
6	-1.920266000	-2.158359000	0.594909000
6	1.902297000	-3.201166000	0.860135000
1	0.807593000	-3.179253000	0.863431000
6	-1.120766000	-1.383568000	1.505523000
6	2.122054000	-0.295807000	1.897422000
6	0.164618000	3.586595000	-2.350934000
1	1.090572000	3.367556000	-2.902269000
6	-1.461826000	1.255184000	0.984960000
6	0.785059000	1.317222000	3.100236000
1	-0.150208000	1.852927000	3.241069000
6	-2.274318000	-3.244963000	-2.201032000
1	-3.180209000	-3.721849000	-1.805892000
5	-0.434176000	0.026897000	1.126413000
6	-1.002222000	-1.937883000	2.816267000
1	-0.400795000	-1.397816000	3.542682000
6	-1.636614000	-3.115994000	3.216471000
1	-1.516660000	-3.478541000	4.235522000
6	3.177587000	-0.063200000	2.800614000
1	4.115136000	-0.596549000	2.694767000
6	-2.445258000	-3.821959000	2.311712000
1	-2.953991000	-4.733980000	2.611533000
6	3.049781000	0.864378000	3.840888000
1	3.875116000	1.035664000	4.526505000
6	1.841320000	1.566437000	3.979492000
1	1.722236000	2.290507000	4.782993000
6	4.209301000	-1.408159000	-0.164327000
1	4.133708000	-1.957323000	-1.114162000
6	-2.742532000	1.245439000	1.617750000
1	-3.032284000	0.349073000	2.160503000
6	-1.149712000	2.470815000	0.273714000
6	1.852379000	3.241371000	0.214971000
1	2.159112000	2.420517000	0.870868000
6	-3.661617000	-0.642434000	-1.323700000
1	-3.597149000	0.018750000	-0.452727000
6	-2.018999000	3.577213000	0.290890000
1	-1.744909000	4.491952000	-0.221802000
6	-3.617483000	2.333787000	1.604519000
1	-4.573149000	2.268919000	2.121124000
6	-2.487060000	-2.947087000	-3.696026000
1	-2.545402000	-3.887662000	-4.260871000
1	-1.653547000	-2.362099000	-4.105689000
1	-3.411707000	-2.393442000	-3.881473000
6	-3.253790000	3.522115000	0.949441000
1	-3.916279000	4.383179000	0.947169000
6	-1.082139000	-4.196396000	-2.004451000
1	-1.252018000	-5.134026000	-2.551319000
1	-0.924294000	-4.442660000	-0.950441000
1	-0.158310000	-3.746393000	-2.389240000
6	0.080214000	5.115075000	-2.180296000
1	0.121601000	5.590913000	-3.171100000
1	0.903658000	5.521132000	-1.588323000
1	-0.859226000	5.428615000	-1.715408000
6	4.667553000	0.025408000	-0.486219000
1	5.665326000	0.010635000	-0.945270000
1	4.724387000	0.638552000	0.420372000
1	3.980313000	0.517304000	-1.182801000
6	-2.577536000	-3.333223000	1.005948000
1	-3.183213000	-3.895869000	0.300796000
6	3.033335000	3.595762000	-0.707100000
1	3.908033000	3.866263000	-0.100591000
1	2.808456000	4.449893000	-1.355078000
1	3.318803000	2.752571000	-1.345571000
6	-1.016236000	3.063812000	-3.187724000
1	-1.069771000	3.598416000	-4.145906000
1	-1.967057000	3.217640000	-2.664745000
1	-0.913327000	1.993986000	-3.398593000
6	2.366903000	-4.179398000	-0.233811000
1	1.964955000	-5.181456000	-0.032755000
1	3.458368000	-4.265836000	-0.272313000
1	2.016078000	-3.871532000	-1.225492000
6	1.433170000	4.420689000	1.106076000
1	2.289172000	4.745422000	1.714531000
1	0.626806000	4.132395000	1.785975000
1	1.094759000	5.283778000	0.523295000
6	2.367091000	-3.619594000	2.263125000
1	1.975736000	-4.619548000	2.498079000
1	1.995615000	-2.925636000	3.022364000
1	3.457933000	-3.663275000	2.346020000
6	-3.757693000	0.238432000	-2.580989000
1	-4.643624000	0.885109000	-2.514350000
1	-3.854610000	-0.355240000	-3.496735000
1	-2.879060000	0.881060000	-2.686023000
6	5.248665000	-2.139042000	0.706362000
1	6.211329000	-2.162050000	0.174703000
1	4.967105000	-3.173463000	0.916993000
1	5.422888000	-1.634520000	1.661334000
6	-4.893679000	-1.544794000	-1.158385000
1	-5.800079000	-0.926774000	-1.093092000
1	-4.835035000	-2.146684000	-0.245739000
1	-5.023396000	-2.223157000	-2.010179000
6	0.854657000	0.369253000	2.037373000
7	1.366457000	-0.573166000	-3.861323000
1	5.033150000	0.147222000	-6.449866000
8	1.927310000	-0.533829000	-7.651175000
6	0.710363000	0.173724000	-8.141368000
6	-0.215140000	0.604251000	-7.016164000

1	1.076754000	1.020385000	-8.727481000
1	0.237206000	-0.544226000	-8.813628000
1	-1.093125000	1.095318000	-7.450710000
1	-0.558305000	-0.253038000	-6.430158000
1	0.267992000	1.315954000	-6.340821000
6	2.940342000	0.255813000	-6.882098000
6	4.288098000	-0.430221000	-7.007744000
1	2.944108000	1.249723000	-7.335980000
1	2.592898000	0.313640000	-5.848549000
1	4.264136000	-1.440683000	-6.589051000
1	4.607405000	-0.486613000	-8.052462000
1	3.129544000	-3.882144000	-5.069235000
8	1.844719000	-2.904039000	-7.271854000
6	1.761348000	-3.822669000	-8.441002000
6	2.716631000	-3.342097000	-9.518094000
1	2.034602000	-4.813602000	-8.074704000
1	0.719472000	-3.834039000	-8.776468000
1	2.460842000	-2.337903000	-9.868652000
1	3.747176000	-3.336163000	-9.151819000
1	2.659960000	-4.022689000	-10.374059000
6	1.239208000	-3.365926000	-5.985525000
6	2.157139000	-4.341126000	-5.270563000
1	1.103900000	-2.448531000	-5.409262000
1	0.262932000	-3.798877000	-6.223492000
1	1.702814000	-4.615823000	-4.311918000
1	2.311910000	-5.262752000	-5.840085000
1	1.872981000	-1.709054000	-7.466054000

^{6}**1**$_{RC}$:

26	0.454658000	-0.185112000	-1.268987000
15	0.203890000	2.295521000	-0.680802000
15	2.087655000	-1.306916000	0.346257000
15	-2.018566000	-1.371380000	-0.955121000
7	1.176631000	-0.428497000	-3.129560000
6	-1.895335000	-2.106789000	0.790962000
6	1.812049000	-3.157357000	0.911627000
1	0.720463000	-3.209609000	0.971074000
6	-1.150883000	-1.377357000	1.776545000
6	2.060096000	-0.257387000	1.921932000
6	-0.093534000	3.385772000	-2.281573000
1	0.834715000	3.160465000	-2.825475000
6	-1.576453000	1.285712000	1.306585000
6	0.900342000	1.380711000	3.285549000
1	-0.007096000	1.922590000	3.538731000
6	-2.212990000	-2.936034000	-2.095123000
1	-3.059978000	-3.526749000	-1.727171000
5	-0.580214000	0.092600000	1.601182000
6	-0.968507000	-2.032222000	3.028965000
1	-0.398773000	-1.515983000	3.796908000
6	-1.505419000	-3.288292000	3.317519000
1	-1.348306000	-3.735838000	4.295726000
6	3.222744000	-0.015905000	2.677468000
1	4.135809000	-0.551926000	2.453347000
6	-2.253582000	-3.966394000	2.344230000
1	-2.682115000	-4.942445000	2.552789000
6	3.234890000	0.915484000	3.723082000
1	4.144032000	1.081237000	4.293992000
6	2.063895000	1.630916000	4.015922000
1	2.057365000	2.365247000	4.817481000
6	3.903534000	-1.319415000	-0.390487000
1	3.735657000	-1.947382000	-1.276543000
6	-2.825900000	1.313206000	1.991189000
1	-3.079121000	0.465721000	2.623239000
6	-1.295695000	2.419015000	0.466923000
6	1.670240000	3.206958000	0.232102000
1	2.035200000	2.418727000	0.896977000
6	-3.836440000	-0.628129000	-0.905538000
1	-3.814990000	-0.080825000	0.042828000
6	-2.186269000	3.507106000	0.412651000
1	-1.951879000	4.369018000	-0.199220000
6	-3.714017000	2.387845000	1.914952000
1	-4.646429000	2.364916000	2.473437000
6	-2.505656000	-2.509233000	-3.545582000
1	-2.522382000	-3.394558000	-4.195669000
1	-1.729708000	-1.832167000	-3.921797000
1	-3.473405000	-2.009648000	-3.646058000
6	-3.388122000	3.505480000	1.130991000
1	-4.058068000	4.358578000	1.074790000
6	-0.936093000	-3.789329000	-2.032164000
1	-1.040629000	-4.670298000	-2.680683000
1	-0.727180000	-4.140196000	-1.017614000
1	-0.071320000	-3.210933000	-2.378939000
6	-0.210963000	4.919031000	-2.188412000
1	-0.154009000	5.341636000	-3.201882000
1	0.587728000	5.374918000	-1.599546000
1	-1.168934000	5.237144000	-1.766325000
6	4.258871000	0.093045000	-0.884526000
1	5.221153000	0.076433000	-1.414734000
1	4.350073000	0.796286000	-0.048815000
1	3.494872000	0.467516000	-1.572920000
6	-2.435128000	-3.370985000	1.089065000
1	-2.991080000	-3.918348000	0.334439000
6	2.797428000	3.568658000	-0.752104000
1	3.694554000	3.862357000	-0.190880000
1	2.527048000	4.410677000	-1.397772000
1	3.068645000	2.723259000	-1.392439000
6	-1.263384000	2.802091000	-3.091446000
1	-1.299813000	3.263737000	-4.087628000
1	-2.220373000	3.002835000	-2.597325000
1	-1.162312000	1.719423000	-3.220017000
6	2.285133000	-4.144435000	-0.171592000
1	1.923043000	-5.153395000	0.067424000
1	3.377425000	-4.195762000	-0.231962000
1	1.902127000	-3.881259000	-1.163210000
6	1.254629000	4.401055000	1.104959000
1	2.127498000	4.769478000	1.662009000
1	0.488960000	4.113250000	1.830447000
1	0.864027000	5.235699000	0.513424000
6	2.374698000	-3.506864000	2.297952000
1	2.064520000	-4.524521000	2.574143000
1	1.998663000	-2.821464000	3.062137000
1	3.469047000	-3.477007000	2.323775000
6	-4.087982000	0.389014000	-2.030379000
1	-5.059743000	0.877810000	-1.875241000

1	-4.113241000	-0.082186000	-3.019043000
1	-3.320042000	1.165230000	-2.044966000
6	5.076589000	-1.937967000	0.393771000
1	5.924853000	-2.079305000	-0.291248000
1	4.838557000	-2.912620000	0.825031000
1	5.426903000	-1.285324000	1.199047000
6	-4.950668000	-1.684080000	-0.836002000
1	-5.918522000	-1.186325000	-0.683612000
1	-4.804076000	-2.381622000	-0.005477000
1	-5.026562000	-2.263584000	-1.763763000
6	0.832681000	0.416973000	2.240281000
7	1.472206000	-0.697641000	-4.269056000
1	5.472307000	-0.629588000	-6.100407000
8	2.532882000	-0.649238000	-7.808099000
6	1.563412000	0.298851000	-8.424930000
6	0.579838000	0.877447000	-7.421734000
1	2.170812000	1.069858000	-8.905914000
1	1.066851000	-0.293255000	-9.196209000
1	-0.120398000	1.532119000	-7.952800000
1	0.006899000	0.090356000	-6.923711000
1	1.084698000	1.471884000	-6.655306000
6	3.529046000	-0.103266000	-6.825007000
6	4.733574000	-1.025766000	-6.805853000
1	3.782664000	0.894003000	-7.193152000
1	3.022842000	-0.046719000	-5.855023000
1	4.459481000	-2.031893000	-6.473945000
1	5.200581000	-1.093117000	-7.792996000
1	2.540035000	-4.230164000	-5.205658000
8	1.891710000	-2.947816000	-7.527274000
6	1.818416000	-3.808663000	-8.737150000
6	3.036689000	-3.550955000	-9.606070000
1	1.790632000	-4.841031000	-8.383796000
1	0.879497000	-3.578084000	-9.251262000
1	3.085404000	-2.509273000	-9.936788000
1	3.957483000	-3.792286000	-9.067200000
1	2.982570000	-4.186124000	-10.496518000
6	0.989803000	-3.289027000	-6.383205000
6	1.542232000	-4.461078000	-5.591089000
1	0.961253000	-2.378079000	-5.774173000
1	-0.000715000	-3.492221000	-6.803156000
1	0.882572000	-4.651902000	-4.737019000
1	1.595989000	-5.381317000	-6.181529000
1	2.199153000	-1.771241000	-7.666112000

^{2}**2**$_{d}$:

26	-1.234759000	0.384903000	-2.034532000
15	-1.707912000	2.758862000	-2.765511000
15	0.656719000	0.418475000	-0.383500000
15	-3.367786000	-0.965599000	-1.508131000
7	-0.515149000	-0.400624000	-3.496753000
6	-3.231548000	-0.944924000	0.374079000
6	0.684882000	-1.085698000	0.857132000
1	-0.384083000	-1.288962000	0.981118000
6	-2.649819000	0.229436000	0.927759000
6	0.349453000	1.957335000	0.654661000
6	-1.991267000	2.948258000	-4.685680000
1	-0.964850000	2.791087000	-5.043908000
6	-3.410966000	2.374631000	-0.584539000
6	-1.296937000	3.571974000	1.368758000
1	-2.323357000	3.926526000	1.398860000
6	-3.231821000	-2.835318000	-1.987603000
1	-4.024484000	-3.367883000	-1.448607000
5	-2.204383000	1.493891000	0.027302000
6	-2.513776000	0.258783000	2.338434000
1	-2.069912000	1.134736000	2.802254000
6	-2.928852000	-0.802180000	3.152923000
1	-2.805292000	-0.738692000	4.230609000
6	1.345387000	2.688383000	1.320951000
1	2.373835000	2.349457000	1.313802000
6	-3.496006000	-1.945626000	2.579183000
1	-3.811279000	-2.777961000	3.201085000
6	1.024406000	3.871866000	2.000048000
1	1.802890000	4.437562000	2.502966000
6	-0.302543000	4.314654000	2.018597000
1	-0.562999000	5.232853000	2.537959000
6	2.437535000	0.460431000	-1.165164000
1	2.423305000	-0.468198000	-1.751401000
6	-4.607516000	2.552363000	0.152746000
1	-4.722183000	2.024287000	1.095492000
6	-3.306850000	3.085657000	-1.812632000
6	-0.524834000	4.253227000	-2.315293000
1	-0.034682000	3.879105000	-1.410898000
6	-5.258492000	-0.531331000	-1.757907000
1	-5.385080000	0.301534000	-1.058274000
6	-4.337320000	3.931719000	-2.251544000
1	-4.238833000	4.481861000	-3.178469000
6	-5.643642000	3.383801000	-0.291596000
1	-6.547581000	3.490093000	0.301941000
6	-3.444261000	-3.022906000	-3.501009000
1	-3.313084000	-4.081217000	-3.761543000
1	-2.712007000	-2.443453000	-4.074414000
1	-4.446506000	-2.726405000	-3.823622000
6	-5.510482000	4.078460000	-1.498487000
1	-6.305304000	4.727877000	-1.852843000
6	-1.866254000	-3.392422000	-1.554389000
1	-1.795092000	-4.453937000	-1.825641000
1	-1.712777000	-3.309343000	-0.474275000
1	-1.054257000	-2.858310000	-2.059880000
6	-2.483647000	4.300278000	-5.237026000
1	-2.319326000	4.323268000	-6.322984000
1	-1.963796000	5.159007000	-4.807454000
1	-3.557606000	4.433867000	-5.074768000
6	2.540728000	1.638812000	-2.145877000
1	3.494736000	1.591632000	-2.686837000
1	2.501428000	2.599373000	-1.619413000
1	1.729617000	1.613257000	-2.878203000
6	-3.647313000	-2.010908000	1.187221000
1	-4.073325000	-2.906765000	0.745669000
6	0.553230000	4.473084000	-3.392634000
1	1.325616000	5.146342000	-2.998760000
1	0.143721000	4.938866000	-4.294314000
1	1.045981000	3.541626000	-3.685426000
6	-2.861316000	1.802911000	-5.229602000
1	-2.806398000	1.786214000	-6.326253000

WILEY-VCH

1	-3.912217000	1.944667000	-4.955936000
1	-2.535253000	0.829366000	-4.852769000
6	1.338342000	-2.318071000	0.205264000
1	1.160627000	-3.200617000	0.833170000
1	2.422243000	-2.203313000	0.101615000
1	0.922012000	-2.526258000	-0.785695000
6	-1.242933000	5.563867000	-1.955252000
1	-0.499790000	6.302083000	-1.623812000
1	-1.960126000	5.420914000	-1.143048000
1	-1.776817000	5.995014000	-2.809168000
6	1.269666000	-0.776556000	2.244203000
1	1.149056000	-1.655416000	2.892126000
1	0.753888000	0.062825000	2.718945000
1	2.337688000	-0.539340000	2.204108000
6	-5.552464000	-0.010497000	-3.172840000
1	-6.600516000	0.311190000	-3.235371000
1	-5.396815000	-0.777109000	-3.939658000
1	-4.921497000	0.847776000	-3.412664000
6	3.660984000	0.413817000	-0.231505000
1	4.562899000	0.263204000	-0.840509000
1	3.616106000	-0.401005000	0.494054000
1	3.797085000	1.351893000	0.314846000
6	-6.211235000	-1.664858000	-1.348769000
1	-7.246451000	-1.300876000	-1.392071000
1	-6.025970000	-2.008883000	-0.326374000
1	-6.139783000	-2.526164000	-2.022584000
6	-1.010019000	2.373080000	0.672859000
7	0.098820000	-0.980521000	-4.420542000
1	-0.154687000	-0.797530000	-5.403426000

^{4}**2**$_{d}$:

26	-1.311754000	0.523556000	-2.019305000
15	-1.738450000	2.908933000	-2.812447000
15	0.617528000	0.294251000	-0.321613000
15	-3.286388000	-1.054390000	-1.514594000
7	-0.631439000	-0.215401000	-3.578901000
6	-3.182977000	-0.960715000	0.366213000
6	0.656602000	-1.164097000	0.968502000
1	-0.409192000	-1.384328000	1.089381000
6	-2.666772000	0.256756000	0.893208000
6	0.331512000	1.887715000	0.646007000
6	-2.059137000	3.138194000	-4.717746000
1	-1.062858000	2.897816000	-5.113236000
6	-3.433782000	2.404918000	-0.628863000
6	-1.251575000	3.608857000	1.251789000
1	-2.260353000	4.011877000	1.241520000
6	-3.104900000	-2.929835000	-1.948693000
1	-3.878660000	-3.471463000	-1.391384000
5	-2.224454000	1.515861000	-0.027385000
6	-2.572946000	0.334212000	2.306280000
1	-2.180342000	1.243310000	2.752622000
6	-2.962287000	-0.717199000	3.145085000
1	-2.871923000	-0.612837000	4.222930000
6	1.342730000	2.596053000	1.315504000
1	2.352359000	2.206498000	1.354048000
6	-3.460626000	-1.903919000	2.595498000
1	-3.755306000	-2.729540000	3.236132000
6	1.063020000	3.818578000	1.940693000
1	1.853977000	4.363024000	2.447494000
6	-0.240395000	4.326649000	1.902057000
1	-0.469383000	5.274985000	2.380619000
6	2.380047000	0.326590000	-1.140739000
1	2.362996000	-0.619045000	-1.699049000
6	-4.653069000	2.523017000	0.086203000
1	-4.773405000	1.953824000	1.003648000
6	-3.329025000	3.169879000	-1.828957000
6	-0.526397000	4.351694000	-2.313311000
1	-0.086639000	3.962941000	-1.389528000
6	-5.170526000	-0.642224000	-1.812785000
1	-5.309322000	0.224728000	-1.157714000
6	-4.371619000	4.012951000	-2.247968000
1	-4.270026000	4.603026000	-3.150219000
6	-5.699064000	3.349686000	-0.340497000
1	-6.615638000	3.409190000	0.240073000
6	-3.320741000	-3.162135000	-3.455260000
1	-3.138652000	-4.218190000	-3.692439000
1	-2.625690000	-2.560472000	-4.051783000
1	-4.339882000	-2.924979000	-3.773472000
6	-5.559729000	4.103223000	-1.511276000
1	-6.361353000	4.752270000	-1.850352000
6	-1.720657000	-3.430917000	-1.508164000
1	-1.611931000	-4.494218000	-1.757602000
1	-1.570168000	-3.319778000	-0.430367000
1	-0.927520000	-2.878508000	-2.024645000
6	-2.457464000	4.527996000	-5.247634000
1	-2.389957000	4.520525000	-6.344036000
1	-1.809569000	5.328073000	-4.884264000
1	-3.490775000	4.782374000	-4.993133000
6	2.438131000	1.479804000	-2.155816000
1	3.368756000	1.421865000	-2.735043000
1	2.418657000	2.451567000	-1.649272000
1	1.595784000	1.433813000	-2.851973000
6	-3.571131000	-2.019348000	1.203533000
1	-3.946075000	-2.946673000	0.780872000
6	0.597172000	4.519987000	-3.352715000
1	1.372783000	5.179446000	-2.942785000
1	0.235822000	4.977532000	-4.279129000
1	1.071847000	3.567313000	-3.606054000
6	-3.038301000	2.063488000	-5.218638000
1	-3.053988000	2.058900000	-6.316305000
1	-4.057767000	2.270579000	-4.874335000
1	-2.749309000	1.066613000	-4.872094000
6	1.345734000	-2.405352000	0.371939000
1	1.167546000	-3.269043000	1.025182000
1	2.429473000	-2.273827000	0.292087000
1	0.959834000	-2.654741000	-0.621307000
6	-1.218440000	5.684071000	-1.984780000
1	-0.469691000	6.398247000	-1.616239000
1	-1.977149000	5.561271000	-1.207154000
1	-1.698849000	6.131293000	-2.861365000
6	1.223470000	-0.790996000	2.347368000
1	1.108382000	-1.645155000	3.028312000
1	0.692888000	0.059670000	2.783666000
1	2.289264000	-0.542665000	2.305943000

6	-5.454084000	-0.198315000	-3.256058000
1	-6.497417000	0.133855000	-3.338110000
1	-5.310034000	-1.010349000	-3.976269000
1	-4.811417000	0.634569000	-3.548414000
6	3.624661000	0.326514000	-0.234675000
1	4.512559000	0.140473000	-0.854191000
1	3.594869000	-0.446384000	0.536665000
1	3.773160000	1.294072000	0.253987000
6	-6.119014000	-1.760904000	-1.355040000
1	-7.156828000	-1.410843000	-1.432927000
1	-5.944342000	-2.047007000	-0.313151000
1	-6.028680000	-2.656795000	-1.979825000
6	-1.008469000	2.369247000	0.609799000
7	-0.045182000	-0.712964000	-4.563575000
1	-0.653908000	-0.776020000	-5.425112000

$^{6}\mathbf{2}_{d}$:

26	-1.193361000	0.410056000	-2.022654000
15	-1.704206000	2.773389000	-2.795649000
15	0.663767000	0.388970000	-0.286129000
15	-3.398559000	-0.941927000	-1.522363000
7	-0.341426000	-0.804916000	-3.495211000
6	-3.242979000	-0.933666000	0.360051000
6	0.715763000	-1.112373000	0.951673000
1	-0.350464000	-1.317697000	1.096065000
6	-2.645780000	0.227029000	0.924920000
6	0.341842000	1.938105000	0.730167000
6	-2.002289000	2.907063000	-4.715037000
1	-0.973112000	2.780006000	-5.075716000
6	-3.397954000	2.372937000	-0.596458000
6	-1.301692000	3.593729000	1.358491000
1	-2.321622000	3.966427000	1.346798000
6	-3.235567000	-2.804070000	-2.018604000
1	-4.030959000	-3.350934000	-1.498182000
5	-2.204811000	1.506767000	0.053394000
6	-2.498385000	0.243481000	2.334057000
1	-2.043184000	1.110314000	2.803319000
6	-2.920689000	-0.820471000	3.140848000
1	-2.790620000	-0.768379000	4.218033000
6	1.324520000	2.660504000	1.423756000
1	2.346535000	2.303906000	1.458128000
6	-3.500618000	-1.952290000	2.558300000
1	-3.819551000	-2.788021000	3.173500000
6	0.999133000	3.858637000	2.074791000
1	1.768086000	4.417083000	2.599541000
6	-0.318141000	4.326573000	2.035782000
1	-0.580748000	5.256953000	2.531122000
6	2.422914000	0.473600000	-1.104202000
1	2.407908000	-0.434839000	-1.720434000
6	-4.594082000	2.547278000	0.141957000
1	-4.709684000	2.015204000	1.081907000
6	-3.292312000	3.084975000	-1.823004000
6	-0.520707000	4.272437000	-2.374475000
1	-0.021102000	3.911232000	-1.469228000
6	-5.297673000	-0.531039000	-1.751814000
1	-5.437269000	0.280943000	-1.030387000
6	-4.322650000	3.933892000	-2.257409000
1	-4.224399000	4.486340000	-3.182662000
6	-5.627373000	3.384423000	-0.297160000
1	-6.529858000	3.490642000	0.297902000
6	-3.425842000	-2.965690000	-3.538308000
1	-3.286096000	-4.018633000	-3.815537000
1	-2.684833000	-2.372032000	-4.085283000
1	-4.424756000	-2.667344000	-3.870527000
6	-5.492883000	4.082826000	-1.501086000
1	-6.285112000	4.737102000	-1.851516000
6	-1.868211000	-3.352964000	-1.578414000
1	-1.789434000	-4.411846000	-1.859055000
1	-1.725589000	-3.281565000	-0.495731000
1	-1.061584000	-2.804864000	-2.077788000
6	-2.549258000	4.229043000	-5.284642000
1	-2.392848000	4.239022000	-6.371584000
1	-2.057431000	5.113203000	-4.872990000
1	-3.626284000	4.324412000	-5.115853000
6	2.482861000	1.686118000	-2.046828000
1	3.424420000	1.673191000	-2.610523000
1	2.438647000	2.628331000	-1.488662000
1	1.658070000	1.669966000	-2.763864000
6	-3.660290000	-2.003569000	1.166654000
1	-4.094494000	-2.892254000	0.719287000
6	0.544344000	4.479517000	-3.466963000
1	1.311583000	5.170355000	-3.094914000
1	0.119616000	4.920734000	-4.374202000
1	1.046310000	3.548127000	-3.744773000
6	-2.828511000	1.713769000	-5.223160000
1	-2.792529000	1.685154000	-6.320119000
1	-3.879189000	1.807757000	-4.930862000
1	-2.444916000	0.761776000	-4.845342000
6	1.358360000	-2.341245000	0.282485000
1	1.208278000	-3.221898000	0.919882000
1	2.437258000	-2.217052000	0.144043000
1	0.912262000	-2.556574000	-0.693548000
6	-1.241136000	5.585298000	-2.028159000
1	-0.498027000	6.328926000	-1.710021000
1	-1.955155000	5.453266000	-1.211278000
1	-1.778045000	6.003408000	-2.886602000
6	1.329570000	-0.802041000	2.325795000
1	1.215125000	-1.678056000	2.978330000
1	0.830073000	0.042603000	2.809050000
1	2.398549000	-0.574100000	2.263064000
6	-5.602754000	0.020183000	-3.153242000
1	-6.658759000	0.315631000	-3.209333000
1	-5.425665000	-0.721826000	-3.939440000
1	-4.994595000	0.901165000	-3.370051000
6	3.660943000	0.419717000	-0.191806000
1	4.556550000	0.322341000	-0.820235000
1	3.649775000	-0.429206000	0.495262000
1	3.778868000	1.335346000	0.395433000
6	-6.231580000	-1.690323000	-1.372223000
1	-7.272586000	-1.342865000	-1.410373000
1	-6.044054000	-2.055064000	-0.357510000
1	-6.143342000	-2.533952000	-2.066016000
6	-1.008585000	2.379362000	0.693692000
7	0.119921000	-0.570652000	-4.693990000

1	-0.416887000	-0.947662000	-5.495200000

^{2}**2**$_{d,RC}$:

26	0.140824000	-0.545791000	-0.945135000
15	0.182441000	1.953624000	-0.911423000
15	2.071136000	-1.619288000	0.287888000
15	-2.263323000	-1.343310000	-0.444069000
7	0.437925000	-1.002492000	-2.675174000
6	-1.900568000	-2.023860000	1.278287000
6	1.763082000	-3.400412000	1.012418000
1	0.702542000	-3.349103000	1.278395000
6	-0.934644000	-1.298031000	2.028654000
6	2.373539000	-0.469022000	1.747926000
6	-0.260279000	2.831246000	-2.595468000
1	0.622534000	2.555358000	-3.187797000
6	-1.179538000	1.354882000	1.439222000
6	1.428604000	1.260087000	3.143714000
1	0.588102000	1.877292000	3.447215000
6	-2.754617000	-2.894702000	-1.493833000
1	-3.615247000	-3.363585000	-1.001973000
5	-0.227344000	0.051121000	1.486956000
6	-0.642598000	-1.792619000	3.324663000
1	0.091957000	-1.270457000	3.930363000
6	-1.265794000	-2.931505000	3.849603000
1	-1.013313000	-3.275517000	4.848761000
6	3.602703000	-0.321615000	2.409432000
1	4.450904000	-0.938272000	2.139464000
6	-2.206610000	-3.629887000	3.084777000
1	-2.686926000	-4.520869000	3.477434000
6	3.752561000	0.630509000	3.427408000
1	4.710189000	0.746442000	3.925636000
6	2.660982000	1.427035000	3.788730000
1	2.766334000	2.171898000	4.572627000
6	3.681405000	-1.765371000	-0.790838000
1	3.324503000	-2.411691000	-1.603936000
6	-2.123232000	1.584063000	2.469605000
1	-2.241142000	0.834818000	3.247802000
6	-1.065078000	2.362256000	0.443109000
6	1.841916000	2.830458000	-0.358479000
1	2.294820000	2.057741000	0.271635000
6	-3.956459000	-0.414334000	-0.119122000
1	-3.736728000	0.112981000	0.815716000
6	-1.839069000	3.531982000	0.497681000
1	-1.728939000	4.301864000	-0.255353000
6	-2.909267000	2.742817000	2.518017000
1	-3.625769000	2.879804000	3.323138000
6	-3.165539000	-2.467978000	-2.915010000
1	-3.389149000	-3.357861000	-3.517486000
1	-2.355251000	-1.920100000	-3.410389000
1	-4.056767000	-1.833781000	-2.917890000
6	-2.767968000	3.722753000	1.529712000
1	-3.369648000	4.626100000	1.557926000
6	-1.593986000	-3.901844000	-1.535441000
1	-1.882491000	-4.775463000	-2.134840000
1	-1.319890000	-4.253734000	-0.536591000
1	-0.705749000	-3.453137000	-1.994537000
6	-0.383909000	4.366655000	-2.616096000
1	-0.362092000	4.711977000	-3.658774000
1	0.424567000	4.872972000	-2.084555000
1	-1.335409000	4.699484000	-2.190473000
6	4.016057000	-0.389340000	-1.388137000
1	4.844513000	-0.483027000	-2.102273000
1	4.323827000	0.318701000	-0.610031000
1	3.154672000	0.030205000	-1.914045000
6	-2.522059000	-3.170007000	1.799013000
1	-3.243226000	-3.723385000	1.205621000
6	2.777075000	3.090491000	-1.553454000
1	3.776646000	3.350299000	-1.181775000
1	2.431121000	3.926169000	-2.170417000
1	2.883075000	2.212672000	-2.198238000
6	-1.487588000	2.184864000	-3.258589000
1	-1.562089000	2.522636000	-4.300955000
1	-2.411720000	2.481529000	-2.752268000
1	-1.424099000	1.093238000	-3.253432000
6	1.936739000	-4.473403000	-0.077874000
1	1.577176000	-5.438312000	0.301809000
1	2.985803000	-4.604430000	-0.362699000
1	1.364324000	-4.238539000	-0.981108000
6	1.651972000	4.091218000	0.500102000
1	2.634297000	4.454886000	0.831056000
1	1.052778000	3.886018000	1.390879000
1	1.168755000	4.903075000	-0.054153000
6	2.556480000	-3.729572000	2.286613000
1	2.227551000	-4.702497000	2.675858000
1	2.390614000	-2.982734000	3.068024000
1	3.633457000	-3.794611000	2.101059000
6	-4.262420000	0.635225000	-1.197496000
1	-5.167511000	1.192353000	-0.921563000
1	-4.440679000	0.183582000	-2.179605000
1	-3.442544000	1.349559000	-1.291449000
6	4.933778000	-2.418128000	-0.178142000
1	5.679820000	-2.563354000	-0.971470000
1	4.735461000	-3.396294000	0.265859000
1	5.396110000	-1.782730000	0.582740000
6	-5.143602000	-1.360548000	0.115784000
1	-6.017986000	-0.773145000	0.426059000
1	-4.940464000	-2.089533000	0.906195000
1	-5.422725000	-1.903532000	-0.794555000
6	1.239681000	0.308332000	2.113080000
7	0.737502000	-1.243124000	-3.871285000
1	3.003481000	1.945159000	-5.059084000
8	2.255857000	-0.233366000	-7.598387000
6	2.792704000	0.246213000	-8.901739000
6	1.727824000	0.956490000	-9.718902000
1	3.650725000	0.885779000	-8.684444000
1	3.146025000	-0.664084000	-9.389986000
1	2.160405000	1.258329000	-10.679235000
1	0.877875000	0.297487000	-9.918805000
1	1.364363000	1.860447000	-9.221016000
6	1.980380000	0.726305000	-6.487211000
6	3.261332000	1.300798000	-5.907248000
1	1.319294000	1.495051000	-6.892653000
1	1.438162000	0.125087000	-5.752543000
1	3.918797000	0.506049000	-5.541585000

1	3.811248000	1.912262000	-6.629140000
1	-0.106948000	-1.801626000	-8.513649000
8	2.125305000	-2.576433000	-7.147538000
6	3.372306000	-3.211531000	-6.635870000
6	3.141708000	-3.827622000	-5.267457000
1	3.696400000	-3.941558000	-7.383350000
1	4.095594000	-2.394399000	-6.600062000
1	2.774765000	-3.072912000	-4.565283000
1	2.422561000	-4.651940000	-5.307836000
1	4.087964000	-4.228998000	-4.888023000
6	1.134094000	-3.451280000	-7.830350000
6	-0.191321000	-2.718338000	-7.922914000
1	1.542660000	-3.706593000	-8.813108000
1	1.052127000	-4.356673000	-7.225876000
1	-0.925273000	-3.367256000	-8.411882000
1	-0.568896000	-2.463629000	-6.928185000
1	2.171586000	-1.383458000	-7.393516000
1	0.309635000	-2.051310000	-4.349136000

^{4}**2**$_{d,RC}$:

26	-0.016423000	-0.349240000	-0.935661000
15	0.216271000	2.197583000	-0.766635000
15	1.934668000	-1.659726000	0.038165000
15	-2.378861000	-1.330314000	-0.532296000
7	0.251478000	-0.765905000	-2.722462000
6	-1.989411000	-2.032374000	1.173001000
6	1.632055000	-3.454097000	0.728471000
1	0.581990000	-3.402539000	1.033336000
6	-1.015651000	-1.318594000	1.926346000
6	2.303872000	-0.548190000	1.516359000
6	-0.259347000	3.184254000	-2.374812000
1	0.566385000	2.889710000	-3.036666000
6	-1.216561000	1.378855000	1.489819000
6	1.462523000	1.128263000	3.037748000
1	0.647998000	1.740281000	3.414095000
6	-2.904183000	-2.854258000	-1.603468000
1	-3.750470000	-3.332173000	-1.095172000
5	-0.307086000	0.045405000	1.415217000
6	-0.700559000	-1.849537000	3.203404000
1	0.042233000	-1.340562000	3.810448000
6	-1.309338000	-3.004505000	3.708362000
1	-1.038462000	-3.373620000	4.693685000
6	3.569754000	-0.441092000	2.115810000
1	4.393281000	-1.054643000	1.772610000
6	-2.260994000	-3.686666000	2.941589000
1	-2.732148000	-4.588999000	3.319058000
6	3.788497000	0.460044000	3.166000000
1	4.773009000	0.540800000	3.616423000
6	2.728103000	1.249837000	3.623876000
1	2.884386000	1.953274000	4.437041000
6	3.492280000	-1.788640000	-1.116268000
1	3.093101000	-2.414549000	-1.925641000
6	-2.194462000	1.530606000	2.504220000
1	-2.366446000	0.705066000	3.189239000
6	-1.032776000	2.490058000	0.616850000
6	1.900221000	2.983095000	-0.173912000
1	2.318263000	2.178672000	0.439851000
6	-4.038462000	-0.361370000	-0.180674000
1	-3.772996000	0.194406000	0.725760000
6	-1.763938000	3.677512000	0.781321000
1	-1.596392000	4.521755000	0.124082000
6	-2.938670000	2.706873000	2.658198000
1	-3.679961000	2.780423000	3.449135000
6	-3.355785000	-2.399175000	-3.003159000
1	-3.584687000	-3.278601000	-3.618602000
1	-2.564368000	-1.832388000	-3.507445000
1	-4.253961000	-1.776818000	-2.969168000
6	-2.722328000	3.788670000	1.797390000
1	-3.289372000	4.707461000	1.911065000
6	-1.745014000	-3.858620000	-1.703864000
1	-2.057816000	-4.726084000	-2.299614000
1	-1.427713000	-4.221262000	-0.721829000
1	-0.879709000	-3.400838000	-2.196611000
6	-0.296162000	4.722379000	-2.313625000
1	-0.369714000	5.116070000	-3.336426000
1	0.596605000	5.155248000	-1.857561000
1	-1.171605000	5.085912000	-1.767239000
6	3.802846000	-0.399361000	-1.697119000
1	4.592323000	-0.479617000	-2.455839000
1	4.157329000	0.285255000	-0.917986000
1	2.916874000	0.037631000	-2.166766000
6	-2.598014000	-3.195249000	1.673982000
1	-3.326605000	-3.735573000	1.077661000
6	2.847351000	3.231139000	-1.361649000
1	3.847173000	3.476776000	-0.981544000
1	2.518371000	4.070914000	-1.982180000
1	2.943990000	2.350367000	-2.003750000
6	-1.564593000	2.634172000	-2.972222000
1	-1.708550000	3.038368000	-3.982695000
1	-2.428138000	2.937480000	-2.370628000
1	-1.551231000	1.542023000	-3.037998000
6	1.762138000	-4.498406000	-0.394872000
1	1.397984000	-5.468391000	-0.033323000
1	2.802226000	-4.635335000	-0.708203000
1	1.171905000	-4.229648000	-1.276555000
6	1.747459000	4.226899000	0.716175000
1	2.736045000	4.525889000	1.089611000
1	1.108484000	4.027952000	1.580682000
1	1.327629000	5.079923000	0.172832000
6	2.467645000	-3.821316000	1.964870000
1	2.141445000	-4.799616000	2.342604000
1	2.339688000	-3.091857000	2.769337000
1	3.536299000	-3.894449000	1.738051000
6	-4.377503000	0.653923000	-1.282902000
1	-5.228647000	1.269146000	-0.962777000
1	-4.660540000	0.167769000	-2.222607000
1	-3.537058000	1.320604000	-1.484510000
6	4.769218000	-2.461330000	-0.580805000
1	5.462419000	-2.619677000	-1.418175000
1	4.582481000	-3.435028000	-0.122311000
1	5.288291000	-1.832310000	0.148166000
6	-5.226717000	-1.283046000	0.134176000
1	-6.082171000	-0.674033000	0.454927000
1	-5.000983000	-1.985727000	0.942048000

1	-5.545188000	-1.854919000	-0.745032000
6	1.200444000	0.228399000	1.974564000
7	0.487555000	-1.016169000	-3.924862000
1	3.049232000	1.995902000	-5.034171000
8	2.316263000	-0.219978000	-7.539202000
6	2.588261000	0.306868000	-8.903804000
6	1.298237000	0.615966000	-9.643328000
1	3.223399000	1.186699000	-8.780701000
1	3.167041000	-0.480275000	-9.390966000
1	1.539412000	0.987949000	-10.645303000
1	0.680175000	-0.280435000	-9.750836000
1	0.713610000	1.387268000	-9.132518000
6	2.023176000	0.723527000	-6.416671000
6	3.303165000	1.324572000	-5.862615000
1	1.343280000	1.478565000	-6.818903000
1	1.495635000	0.114918000	-5.673467000
1	3.967822000	0.545515000	-5.476479000
1	3.845767000	1.910760000	-6.611285000
1	0.339209000	-3.800117000	-5.794607000
8	2.385668000	-2.577257000	-7.149287000
6	3.701859000	-3.196177000	-6.827483000
6	3.933362000	-3.212453000	-5.327023000
1	3.694466000	-4.198813000	-7.261725000
1	4.447082000	-2.591995000	-7.350911000
1	3.898399000	-2.199419000	-4.914355000
1	3.185997000	-3.824203000	-4.814113000
1	4.924210000	-3.631350000	-5.118180000
6	1.297638000	-3.426980000	-7.701048000
6	0.723060000	-4.362024000	-6.651807000
1	0.556051000	-2.706950000	-8.052406000
1	1.707981000	-3.966526000	-8.560028000
1	-0.106650000	-4.926040000	-7.092108000
1	1.465231000	-5.084014000	-6.297553000
1	2.345346000	-1.362808000	-7.341975000
1	0.144350000	-1.971558000	-4.228722000

^{6}**2**$_{d,RC}$:

26	-0.004446000	-0.381457000	-1.044712000
15	0.028032000	2.213416000	-0.751426000
15	1.986571000	-1.486937000	0.249679000
15	-2.288604000	-1.526407000	-0.375054000
7	0.297294000	-0.743074000	-2.883701000
6	-1.898243000	-2.086311000	1.388404000
6	1.749395000	-3.268362000	0.997432000
1	0.684624000	-3.264739000	1.249523000
6	-0.991014000	-1.290584000	2.159425000
6	2.233806000	-0.291727000	1.688380000
6	-0.495934000	3.218483000	-2.344949000
1	0.318850000	2.926894000	-3.022565000
6	-1.419103000	1.347583000	1.550326000
6	1.340958000	1.487590000	3.070886000
1	0.499029000	2.081141000	3.415736000
6	-2.713416000	-3.163099000	-1.325550000
1	-3.509885000	-3.676813000	-0.774351000
5	-0.417551000	0.134219000	1.740528000
6	-0.627343000	-1.819460000	3.430371000
1	0.069291000	-1.252443000	4.041223000
6	-1.142851000	-3.017253000	3.930438000
1	-0.843543000	-3.369113000	4.914015000
6	3.507303000	-0.036236000	2.229688000
1	4.360303000	-0.622431000	1.913505000
6	-2.052056000	-3.759603000	3.164342000
1	-2.465443000	-4.690135000	3.541205000
6	3.703911000	0.973475000	3.178678000
1	4.694487000	1.151353000	3.586099000
6	2.610392000	1.748878000	3.590061000
1	2.746949000	2.541201000	4.321071000
6	3.626415000	-1.591068000	-0.806358000
1	3.288309000	-2.252838000	-1.616050000
6	-2.533257000	1.452624000	2.432539000
1	-2.698149000	0.649086000	3.145109000
6	-1.256864000	2.427945000	0.617120000
6	1.672641000	3.074167000	-0.155998000
1	2.116283000	2.299899000	0.477159000
6	-4.008632000	-0.633914000	-0.139583000
1	-3.807338000	-0.000279000	0.730404000
6	-2.117554000	3.540177000	0.646925000
1	-1.969437000	4.359349000	-0.044809000
6	-3.394263000	2.551581000	2.444354000
1	-4.218906000	2.588563000	3.151055000
6	-3.220193000	-2.847196000	-2.744841000
1	-3.361273000	-3.783242000	-3.300673000
1	-2.497852000	-2.235700000	-3.298801000
1	-4.178941000	-2.321524000	-2.739350000
6	-3.180390000	3.614282000	1.554465000
1	-3.830462000	4.483818000	1.561840000
6	-1.472439000	-4.068276000	-1.379663000
1	-1.707963000	-4.994495000	-1.919880000
1	-1.118459000	-4.340367000	-0.381429000
1	-0.651978000	-3.569442000	-1.909253000
6	-0.536473000	4.758017000	-2.305701000
1	-0.635442000	5.134260000	-3.333300000
1	0.367439000	5.198130000	-1.880483000
1	-1.395794000	5.135976000	-1.744235000
6	3.921913000	-0.209305000	-1.414324000
1	4.745901000	-0.285235000	-2.135870000
1	4.217725000	0.509966000	-0.642618000
1	3.048651000	0.193249000	-1.937201000
6	-2.417276000	-3.290752000	1.896623000
1	-3.101760000	-3.886706000	1.301476000
6	2.622206000	3.337751000	-1.338769000
1	3.610079000	3.621953000	-0.953943000
1	2.272238000	4.156494000	-1.975559000
1	2.754077000	2.451512000	-1.967444000
6	-1.807683000	2.655542000	-2.917625000
1	-1.995043000	3.085875000	-3.910105000
1	-2.659260000	2.912615000	-2.278648000
1	-1.772158000	1.566176000	-3.021659000
6	1.994892000	-4.350645000	-0.069797000
1	1.655393000	-5.321966000	0.312127000
1	3.056493000	-4.450146000	-0.317522000
1	1.447036000	-4.148903000	-0.995887000
6	1.458829000	4.326508000	0.708703000
1	2.427258000	4.666427000	1.099932000

1	0.807883000	4.116288000	1.561482000
1	1.021480000	5.155280000	0.142578000
6	2.543527000	-3.530244000	2.286284000
1	2.272233000	-4.516833000	2.685565000
1	2.317416000	-2.783802000	3.052394000
1	3.624957000	-3.527514000	2.115522000
6	-4.367014000	0.281558000	-1.320145000
1	-5.273444000	0.852193000	-1.078217000
1	-4.568376000	-0.280768000	-2.237800000
1	-3.566505000	0.994899000	-1.526257000
6	4.902312000	-2.209486000	-0.204182000
1	5.633146000	-2.364254000	-1.010109000
1	4.728027000	-3.178715000	0.268384000
1	5.374468000	-1.551502000	0.530932000
6	-5.151257000	-1.599596000	0.211083000
1	-6.051153000	-1.021305000	0.459050000
1	-4.909862000	-2.222953000	1.077745000
1	-5.407347000	-2.257439000	-0.627544000
6	1.088965000	0.451784000	2.127685000
7	0.505299000	-0.944251000	-3.992523000
1	4.045993000	1.055865000	-4.983923000
8	2.895254000	-0.510185000	-7.800869000
6	3.326551000	0.133774000	-9.072378000
6	2.178804000	0.862308000	-9.750359000
1	4.155130000	0.798585000	-8.819223000
1	3.708886000	-0.690393000	-9.677114000
1	2.540939000	1.309740000	-10.682643000
1	1.363857000	0.175219000	-9.997156000
1	1.783044000	1.669015000	-9.125699000
6	2.799335000	0.307765000	-6.557877000
6	4.159449000	0.468933000	-5.902376000
1	2.351039000	1.264185000	-6.837812000
1	2.098438000	-0.240871000	-5.926319000
1	4.584995000	-0.503274000	-5.637297000
1	4.865651000	0.997969000	-6.549993000
1	-0.144073000	-2.744907000	-6.037086000
8	1.978061000	-2.715548000	-7.752630000
6	2.834803000	-3.925076000	-7.894178000
6	4.162177000	-3.676724000	-7.202114000
1	2.285905000	-4.746874000	-7.430354000
1	2.948802000	-4.120959000	-8.964709000
1	4.709908000	-2.847960000	-7.660182000
1	4.015593000	-3.462301000	-6.139386000
1	4.780844000	-4.576083000	-7.287421000
6	0.531659000	-2.845670000	-8.089190000
6	-0.251650000	-3.394628000	-6.910362000
1	0.221537000	-1.831663000	-8.349196000
1	0.463314000	-3.478452000	-8.978716000
1	-1.313182000	-3.443673000	-7.177624000
1	0.070797000	-4.405591000	-6.642207000
1	2.461581000	-1.619863000	-7.796528000

^{2}**3**$_{dd}$:

26	-0.070470000	-0.172725000	-1.238473000
15	-0.543539000	2.184964000	-0.578557000
15	2.162989000	-0.610918000	-0.281168000
15	-2.027878000	-1.399266000	-0.118924000
7	0.128284000	-0.754649000	-2.842515000
6	-1.286140000	-2.053348000	1.504721000
6	2.360476000	-2.456351000	0.333829000
1	1.369550000	-2.667618000	0.746066000
6	-0.353727000	-1.238053000	2.199606000
6	2.529044000	0.466243000	1.227048000
6	-1.577285000	2.911313000	-2.062503000
1	-0.840466000	2.827144000	-2.871292000
6	-1.255825000	1.312243000	1.990633000
6	1.662870000	1.713506000	3.118087000
1	0.830256000	2.032478000	3.737459000
6	-2.447665000	-3.006018000	-1.134517000
1	-3.253326000	-3.520336000	-0.594948000
5	-0.057613000	0.281990000	1.885762000
6	0.251214000	-1.772052000	3.363717000
1	0.981187000	-1.174409000	3.900656000
6	-0.079645000	-3.041565000	3.850339000
1	0.394796000	-3.418648000	4.750463000
6	3.814172000	0.940878000	1.528104000
1	4.660069000	0.658035000	0.917483000
6	-1.017742000	-3.820059000	3.167570000
1	-1.285963000	-4.805718000	3.533503000
6	4.028273000	1.789403000	2.623241000
1	5.029795000	2.145854000	2.840274000
6	2.949232000	2.176446000	3.420368000
1	3.101202000	2.841493000	4.264265000
6	3.521943000	-0.387327000	-1.653287000
1	3.152494000	-1.102337000	-2.399396000
6	-2.023693000	1.304956000	3.177220000
1	-1.839967000	0.539585000	3.925395000
6	-1.538069000	2.307416000	1.021669000
6	0.948403000	3.435251000	-0.365828000
1	1.731641000	2.787326000	0.039133000
6	-3.766381000	-0.690998000	0.473034000
1	-3.497472000	0.316430000	0.803755000
6	-2.532387000	3.266603000	1.260108000
1	-2.744059000	4.033480000	0.528247000
6	-3.015457000	2.265765000	3.412684000
1	-3.588651000	2.238666000	4.333779000
6	-2.933148000	-2.648408000	-2.554403000
1	-3.101421000	-3.576471000	-3.115495000
1	-2.174253000	-2.065780000	-3.086197000
1	-3.866759000	-2.085558000	-2.562827000
6	-3.268549000	3.249618000	2.453722000
1	-4.035717000	3.997998000	2.623324000
6	-1.220077000	-3.928420000	-1.238948000
1	-1.485829000	-4.820444000	-1.819838000
1	-0.841372000	-4.258496000	-0.269859000
1	-0.408146000	-3.416871000	-1.764610000
6	-2.038256000	4.380552000	-1.979429000
1	-2.256111000	4.737317000	-2.994290000
1	-1.293360000	5.051672000	-1.548030000
1	-2.963157000	4.478448000	-1.403372000
6	3.433131000	1.017327000	-2.268096000
1	4.125277000	1.090924000	-3.116308000
1	3.708686000	1.793538000	-1.545623000
1	2.423822000	1.221730000	-2.634186000

6	-1.610539000	-3.326989000	1.997294000
1	-2.330434000	-3.948841000	1.477569000
6	1.434039000	3.991189000	-1.717895000
1	2.410993000	4.469262000	-1.573865000
1	0.755875000	4.750537000	-2.117221000
1	1.557831000	3.212936000	-2.475619000
6	-2.764266000	1.995280000	-2.397226000
1	-3.245706000	2.342065000	-3.320367000
1	-3.515134000	2.020940000	-1.600495000
1	-2.448322000	0.958846000	-2.546335000
6	2.608522000	-3.414253000	-0.844125000
1	2.525947000	-4.448361000	-0.488062000
1	3.606584000	-3.294134000	-1.275653000
1	1.872302000	-3.284793000	-1.643004000
6	0.688989000	4.560913000	0.649065000
1	1.602046000	5.161106000	0.754129000
1	0.430634000	4.168873000	1.636026000
1	-0.113868000	5.234036000	0.329613000
6	3.394415000	-2.636807000	1.456279000
1	3.373477000	-3.680960000	1.794014000
1	3.171747000	-2.002188000	2.317731000
1	4.414139000	-2.416129000	1.125736000
6	-4.798906000	-0.580208000	-0.661257000
1	-5.644906000	0.022617000	-0.307442000
1	-5.194977000	-1.562497000	-0.938706000
1	-4.401399000	-0.099209000	-1.557181000
6	4.979951000	-0.759450000	-1.323444000
1	5.532309000	-0.858387000	-2.266906000
1	5.077115000	-1.705298000	-0.787346000
1	5.483278000	0.018203000	-0.742346000
6	-4.362203000	-1.461845000	1.661189000
1	-5.311019000	-0.990291000	1.948146000
1	-3.706137000	-1.454987000	2.533997000
1	-4.580070000	-2.504630000	1.400956000
6	1.419666000	0.839563000	2.032643000
7	0.592724000	-1.545119000	-3.796328000
1	0.545554000	-1.244542000	-4.762528000
1	0.948033000	-2.475001000	-3.601798000

^{4}**3**$_{dd}$:

26	-0.144641000	-0.201687000	-1.292580000
15	-0.495162000	2.332647000	-0.607094000
15	2.133729000	-0.822337000	-0.239510000
15	-1.983887000	-1.573729000	-0.068845000
7	-0.030381000	-0.828428000	-2.996700000
6	-1.259735000	-2.092099000	1.609281000
6	2.419210000	-2.620370000	0.463330000
1	1.442195000	-2.845463000	0.903214000
6	-0.361151000	-1.213981000	2.275137000
6	2.494216000	0.359745000	1.199242000
6	-1.514149000	3.102843000	-2.075903000
1	-0.769301000	3.026227000	-2.879312000
6	-1.291824000	1.322345000	1.931137000
6	1.651063000	1.814500000	2.956813000
1	0.822673000	2.212833000	3.534494000
6	-2.324531000	-3.254523000	-0.984377000
1	-3.071232000	-3.796096000	-0.389837000
5	-0.089512000	0.297373000	1.908749000
6	0.258630000	-1.678080000	3.462015000
1	0.967718000	-1.034642000	3.973435000
6	-0.030818000	-2.935197000	4.003792000
1	0.452544000	-3.255423000	4.920926000
6	3.788075000	0.827649000	1.474034000
1	4.631501000	0.461961000	0.905915000
6	-0.935519000	-3.775296000	3.350682000
1	-1.169721000	-4.753937000	3.756570000
6	4.019715000	1.768387000	2.487893000
1	5.030965000	2.109162000	2.684381000
6	2.947518000	2.265738000	3.230496000
1	3.112648000	3.003105000	4.009181000
6	3.478447000	-0.590823000	-1.630301000
1	3.111968000	-1.323682000	-2.360673000
6	-2.133662000	1.264891000	3.068206000
1	-1.992600000	0.466300000	3.789798000
6	-1.521719000	2.362810000	0.986760000
6	0.990875000	3.565888000	-0.310060000
1	1.733355000	2.917488000	0.165666000
6	-3.744311000	-0.881481000	0.441241000
1	-3.474826000	0.066245000	0.918627000
6	-2.534038000	3.305762000	1.217079000
1	-2.709540000	4.103182000	0.508782000
6	-3.135555000	2.216133000	3.294305000
1	-3.757501000	2.149064000	4.180847000
6	-2.884091000	-2.999611000	-2.397536000
1	-3.010792000	-3.962541000	-2.908514000
1	-2.180969000	-2.396337000	-2.981835000
1	-3.852904000	-2.499106000	-2.392652000
6	-3.334176000	3.240819000	2.367005000
1	-4.108037000	3.984574000	2.526124000
6	-1.036429000	-4.088891000	-1.093152000
1	-1.266699000	-5.046625000	-1.576950000
1	-0.579583000	-4.305161000	-0.124464000
1	-0.304722000	-3.564125000	-1.715375000
6	-1.971827000	4.571343000	-1.993628000
1	-2.240756000	4.910683000	-3.002491000
1	-1.201041000	5.245689000	-1.615168000
1	-2.864965000	4.686614000	-1.372619000
6	3.350967000	0.808016000	-2.256098000
1	4.029869000	0.889786000	-3.114530000
1	3.623069000	1.592480000	-1.541293000
1	2.334227000	0.998367000	-2.612408000
6	-1.535483000	-3.355664000	2.155476000
1	-2.220753000	-4.029825000	1.654651000
6	1.571386000	4.067842000	-1.644886000
1	2.523780000	4.576691000	-1.450633000
1	0.910436000	4.789061000	-2.134988000
1	1.769977000	3.252818000	-2.347219000
6	-2.692479000	2.180513000	-2.428927000
1	-3.185436000	2.542495000	-3.339942000
1	-3.438516000	2.169297000	-1.627393000
1	-2.360536000	1.152318000	-2.609266000
6	2.677457000	-3.617830000	-0.680714000
1	2.613320000	-4.639919000	-0.287513000
1	3.674538000	-3.495848000	-1.114898000

1	1.941643000	-3.526178000	-1.485420000
6	0.677752000	4.720730000	0.654055000
1	1.596361000	5.294091000	0.834110000
1	0.315658000	4.356727000	1.619193000
1	-0.068649000	5.411858000	0.248774000
6	3.477281000	-2.719322000	1.573296000
1	3.502035000	-3.749149000	1.952856000
1	3.246200000	-2.058685000	2.413220000
1	4.482527000	-2.474871000	1.215509000
6	-4.651760000	-0.578764000	-0.764727000
1	-5.451589000	0.101587000	-0.446248000
1	-5.131984000	-1.487809000	-1.140473000
1	-4.118795000	-0.103471000	-1.591831000
6	4.951874000	-0.922572000	-1.326299000
1	5.486435000	-1.032611000	-2.278977000
1	5.081614000	-1.852361000	-0.769503000
1	5.450313000	-0.119535000	-0.775814000
6	-4.477973000	-1.760293000	1.466062000
1	-5.430757000	-1.283538000	1.730818000
1	-3.904108000	-1.893522000	2.386436000
1	-4.711514000	-2.750812000	1.057203000
6	1.391532000	0.843210000	1.959603000
7	0.021478000	-0.555008000	-4.264002000
1	-0.077994000	0.392385000	-4.627713000
1	0.159404000	-1.289819000	-4.953575000

$^{6}\mathbf{3}_{dd}$:

26	-0.181006000	-0.186035000	-1.276634000
15	-0.534282000	2.341978000	-0.697670000
15	2.121849000	-0.877438000	-0.181599000
15	-1.983277000	-1.649555000	0.039033000
7	-0.150914000	-0.618170000	-3.116397000
6	-1.194755000	-2.130205000	1.692967000
6	2.420557000	-2.657367000	0.564303000
1	1.444835000	-2.885511000	1.004634000
6	-0.345442000	-1.189917000	2.341393000
6	2.483340000	0.349610000	1.216003000
6	-1.496444000	3.211936000	-2.155453000
1	-0.747041000	3.096602000	-2.950089000
6	-1.315420000	1.329430000	1.870257000
6	1.631082000	1.865659000	2.916444000
1	0.799803000	2.286655000	3.473458000
6	-2.273175000	-3.319173000	-0.893525000
1	-2.878504000	-3.955684000	-0.237922000
5	-0.104135000	0.314010000	1.910477000
6	0.288409000	-1.597370000	3.541228000
1	0.959772000	-0.908527000	4.044196000
6	0.065760000	-2.858913000	4.104548000
1	0.557155000	-3.132574000	5.032361000
6	3.777933000	0.816140000	1.488328000
1	4.624771000	0.424947000	0.942509000
6	-0.774802000	-3.766320000	3.456125000
1	-0.946456000	-4.752919000	3.873675000
6	4.004659000	1.788548000	2.473090000
1	5.015987000	2.128917000	2.669520000
6	2.928207000	2.317032000	3.187645000
1	3.090859000	3.078169000	3.943570000
6	3.442249000	-0.691014000	-1.600657000
1	3.087762000	-1.472768000	-2.284759000
6	-2.161803000	1.292740000	3.006710000
1	-2.009281000	0.518055000	3.751829000
6	-1.559799000	2.344970000	0.899191000
6	0.996854000	3.504947000	-0.351304000
1	1.694013000	2.821497000	0.142192000
6	-3.758631000	-1.082211000	0.628768000
1	-3.513903000	-0.449209000	1.488002000
6	-2.596356000	3.268383000	1.099257000
1	-2.789076000	4.036941000	0.364292000
6	-3.179743000	2.232291000	3.206438000
1	-3.799207000	2.180323000	4.095607000
6	-3.049282000	-3.061691000	-2.198911000
1	-3.141559000	-4.003310000	-2.754438000
1	-2.516450000	-2.345220000	-2.834019000
1	-4.060021000	-2.686043000	-2.019629000
6	-3.398759000	3.222721000	2.247792000
1	-4.188297000	3.954708000	2.381750000
6	-0.932951000	-4.010958000	-1.188109000
1	-1.119030000	-4.964214000	-1.698862000
1	-0.363960000	-4.224638000	-0.278820000
1	-0.318862000	-3.388809000	-1.848499000
6	-1.829763000	4.712269000	-2.043693000
1	-2.108606000	5.078930000	-3.040207000
1	-0.991386000	5.317368000	-1.695324000
1	-2.684049000	4.899356000	-1.386893000
6	3.274365000	0.665569000	-2.304171000
1	3.954876000	0.718462000	-3.163687000
1	3.520779000	1.496372000	-1.634320000
1	2.253927000	0.799919000	-2.675870000
6	-1.390420000	-3.403402000	2.250117000
1	-2.017635000	-4.131501000	1.748370000
6	1.640431000	3.979591000	-1.667290000
1	2.609405000	4.442336000	-1.442361000
1	1.031370000	4.731027000	-2.178227000
1	1.821989000	3.155470000	-2.363478000
6	-2.743035000	2.408261000	-2.561194000
1	-3.141828000	2.810099000	-3.501248000
1	-3.533130000	2.491967000	-1.808007000
1	-2.518623000	1.349069000	-2.718042000
6	2.700769000	-3.680361000	-0.551893000
1	2.657024000	-4.691255000	-0.128185000
1	3.696227000	-3.550954000	-0.987323000
1	1.964707000	-3.628659000	-1.359631000
6	0.709648000	4.668691000	0.610584000
1	1.649684000	5.192987000	0.825679000
1	0.298329000	4.317073000	1.560509000
1	0.012815000	5.399417000	0.188360000
6	3.474303000	-2.713859000	1.681513000
1	3.504026000	-3.731789000	2.091340000
1	3.235351000	-2.030772000	2.500855000
1	4.479353000	-2.474104000	1.319944000
6	-4.467142000	-0.213254000	-0.420394000
1	-5.394230000	0.188502000	0.007935000
1	-4.737260000	-0.779216000	-1.317520000
1	-3.843389000	0.629274000	-0.723596000

6	4.924727000	-0.967545000	-1.287247000
1	5.455913000	-1.126718000	-2.234681000
1	5.081203000	-1.855280000	-0.671464000
1	5.404473000	-0.116799000	-0.794765000
6	-4.637969000	-2.245474000	1.110624000
1	-5.558942000	-1.839499000	1.548424000
1	-4.145261000	-2.845989000	1.881556000
1	-4.931939000	-2.906987000	0.288292000
6	1.376018000	0.864479000	1.948511000
7	-0.357438000	-0.203688000	-4.382797000
1	-0.321004000	0.780688000	-4.624724000
1	-0.264363000	-0.866329000	-5.143120000

$^2\mathbf{3}_{dd,RC}$:

26	-2.094539000	-1.202013000	-11.069622000
15	-2.944469000	1.156169000	-10.891189000
15	-1.509127000	-1.904260000	-8.770832000
15	-4.226239000	-2.550653000	-11.876096000
7	-0.811927000	-1.819114000	-12.077150000
6	-5.047608000	-3.226661000	-10.298169000
6	-1.864675000	-3.757941000	-8.260739000
1	-2.814984000	-3.958789000	-8.763462000
6	-5.013835000	-2.434796000	-9.118634000
6	-2.416305000	-0.836095000	-7.496758000
6	-2.467693000	2.005333000	-12.579528000
1	-1.375190000	1.904205000	-12.539620000
6	-5.398079000	0.136228000	-9.900229000
6	-4.376666000	0.480248000	-6.926080000
1	-5.373480000	0.838682000	-7.163544000
6	-3.713760000	-4.152044000	-12.857727000
1	-4.642258000	-4.676413000	-13.117020000
5	-4.594212000	-0.916638000	-9.041479000
6	-5.535286000	-2.995655000	-7.926775000
1	-5.497590000	-2.416678000	-7.009221000
6	-6.115225000	-4.268809000	-7.904117000
1	-6.516994000	-4.666466000	-6.977979000
6	-1.809642000	-0.402108000	-6.309009000
1	-0.811063000	-0.725806000	-6.053796000
6	-6.164391000	-5.024434000	-9.078069000
1	-6.610648000	-6.013476000	-9.077123000
6	-2.482168000	0.455761000	-5.426917000
1	-1.994831000	0.775045000	-4.511687000
6	-3.769078000	0.899267000	-5.736554000
1	-4.294160000	1.572365000	-5.066903000
6	0.416073000	-1.687885000	-8.564882000
1	0.768300000	-2.368895000	-9.349683000
6	-6.808605000	0.085225000	-9.799221000
1	-7.271027000	-0.720173000	-9.236939000
6	-4.822593000	1.184864000	-10.666515000
6	-2.225198000	2.340010000	-9.512228000
1	-2.104345000	1.658064000	-8.665280000
6	-5.772503000	-1.830720000	-12.854080000
1	-5.929936000	-0.875630000	-12.343428000
6	-5.647404000	2.142370000	-11.275097000
1	-5.218845000	2.946192000	-11.856508000
6	-7.624487000	1.051319000	-10.399484000
1	-8.702964000	0.990542000	-10.297265000

6	-2.960758000	-3.787940000	-14.153273000
1	-2.611124000	-4.712434000	-14.630957000
1	-2.085695000	-3.168875000	-13.928858000
1	-3.581542000	-3.258785000	-14.876414000
6	-7.041721000	2.082940000	-11.138674000
1	-7.660648000	2.836893000	-11.613735000
6	-2.823593000	-5.068399000	-11.999778000
1	-2.601956000	-5.985282000	-12.560694000
1	-3.285784000	-5.358740000	-11.053705000
1	-1.873144000	-4.571908000	-11.781309000
6	-2.798792000	3.496845000	-12.784514000
1	-2.182902000	3.878920000	-13.608999000
1	-2.594674000	4.115093000	-11.908868000
1	-3.842683000	3.647793000	-13.073969000
6	0.839508000	-0.262437000	-8.954730000
1	1.933169000	-0.182476000	-8.917227000
1	0.428256000	0.482418000	-8.265231000
1	0.518436000	-0.011226000	-9.969701000
6	-5.624052000	-4.506094000	-10.263277000
1	-5.660074000	-5.115211000	-11.159096000
6	-0.835599000	2.875866000	-9.903834000
1	-0.367353000	3.330878000	-9.022345000
1	-0.894874000	3.647480000	-10.676811000
1	-0.168617000	2.086205000	-10.261192000
6	-2.984255000	1.170447000	-13.762162000
1	-2.605180000	1.591797000	-14.701907000
1	-4.078204000	1.185512000	-13.808444000
1	-2.653862000	0.129611000	-13.700213000
6	-0.802458000	-4.707978000	-8.841085000
1	-1.131360000	-5.743476000	-8.690360000
1	0.168156000	-4.598319000	-8.348679000
1	-0.663385000	-4.565308000	-9.916956000
6	-3.178650000	3.466537000	-9.083431000
1	-2.720319000	4.025226000	-8.257256000
1	-4.136781000	3.075108000	-8.731827000
1	-3.377349000	4.176366000	-9.892767000
6	-2.070946000	-3.966776000	-6.752287000
1	-2.352028000	-5.012822000	-6.575168000
1	-2.872990000	-3.335111000	-6.362274000
1	-1.165472000	-3.764887000	-6.172072000
6	-5.478960000	-1.547593000	-14.338026000
1	-6.254986000	-0.878354000	-14.730732000
1	-5.514061000	-2.465752000	-14.932990000
1	-4.511869000	-1.065930000	-14.499240000
6	1.086843000	-2.119037000	-7.245553000
1	2.164684000	-2.222621000	-7.425995000
1	0.726303000	-3.075548000	-6.863588000
1	0.969892000	-1.369753000	-6.458100000
6	-7.045556000	-2.679333000	-12.712193000
1	-7.863308000	-2.190156000	-13.257504000
1	-7.356652000	-2.790556000	-11.671203000
1	-6.918773000	-3.679972000	-13.143225000
6	-3.731694000	-0.405267000	-7.821403000
7	0.389423000	-2.380616000	-12.186216000
1	1.021485000	-2.089269000	-12.926711000
1	0.686184000	-3.125767000	-11.565499000
1	4.667001000	-3.450664000	-18.404590000

8	2.159443000	-2.553022000	-16.243874000
6	1.047923000	-3.554332000	-16.195272000
6	-0.137280000	-3.103646000	-17.027711000
1	1.466229000	-4.505036000	-16.530860000
1	0.807027000	-3.624964000	-15.133533000
1	-0.933091000	-3.851403000	-16.938628000
1	-0.527134000	-2.144775000	-16.674737000
1	0.115194000	-3.015767000	-18.088550000
6	3.034418000	-2.451091000	-17.454574000
6	4.032814000	-3.591735000	-17.522451000
1	2.362316000	-2.422179000	-18.313324000
1	3.516029000	-1.477967000	-17.347883000
1	4.677346000	-3.610401000	-16.638777000
1	3.540559000	-4.563679000	-17.621861000
1	5.243256000	-0.267941000	-13.723783000
8	2.651962000	-1.353116000	-14.200649000
6	2.428765000	0.128271000	-14.178162000
6	1.063339000	0.427690000	-14.767859000
1	2.498761000	0.428105000	-13.129153000
1	3.235181000	0.598277000	-14.745899000
1	0.994192000	0.103739000	-15.810521000
1	0.265380000	-0.049863000	-14.191120000
1	0.898632000	1.510283000	-14.743841000
6	3.798614000	-1.867991000	-13.383256000
6	5.149549000	-1.350496000	-13.848008000
1	3.581686000	-1.584143000	-12.349669000
1	3.717825000	-2.952008000	-13.482539000
1	5.929760000	-1.820826000	-13.239325000
1	5.342656000	-1.604881000	-14.894396000
1	2.413814000	-1.979042000	-15.313370000

$^{4}\mathbf{3}_{dd,RC}$:

26	0.957533000	0.853340000	2.367188000
15	0.002915000	3.257708000	2.435436000
15	2.756245000	0.746972000	4.267795000
15	-1.080144000	-0.719126000	3.204632000
7	1.841275000	0.645940000	0.780029000
6	-0.821243000	-0.969219000	5.064570000
6	2.989074000	-0.908807000	5.269639000
1	1.953190000	-1.234244000	5.407526000
6	-0.395229000	0.147290000	5.837035000
6	2.385959000	2.091267000	5.555533000
6	-0.491673000	3.765419000	0.617816000
1	0.505879000	3.740863000	0.159761000
6	-1.486665000	2.410722000	4.731856000
6	0.766592000	3.542226000	6.646597000
1	-0.261504000	3.843841000	6.821559000
6	-0.812889000	-2.467374000	2.416228000
1	-1.496114000	-3.160235000	2.919854000
5	-0.241514000	1.634537000	5.319328000
6	-0.151204000	-0.063621000	7.216386000
1	0.195126000	0.765672000	7.824949000
6	-0.353617000	-1.309457000	7.820881000
1	-0.170773000	-1.432685000	8.883198000
6	3.410489000	2.802511000	6.198746000
1	4.445481000	2.539670000	6.031409000
6	-0.770962000	-2.393872000	7.045900000
1	-0.916671000	-3.369406000	7.497920000
6	3.122436000	3.863189000	7.069408000
1	3.933386000	4.391814000	7.559419000
6	1.796186000	4.237316000	7.291494000
1	1.561373000	5.065382000	7.952049000
6	4.489701000	1.128941000	3.457708000
1	4.497673000	0.370335000	2.664203000
6	-2.654729000	2.355183000	5.532108000
1	-2.666156000	1.707426000	6.403568000
6	-1.511673000	3.251113000	3.581478000
6	1.119200000	4.736929000	3.063122000
1	1.716105000	4.253776000	3.842526000
6	-3.023002000	-0.464485000	3.159489000
1	-3.171561000	0.229201000	3.992998000
6	-2.662951000	3.997610000	3.287976000
1	-2.690650000	4.636097000	2.416267000
6	-3.790556000	3.119634000	5.241120000
1	-4.663006000	3.061659000	5.883458000
6	-1.173295000	-2.422559000	0.920016000
1	-0.933999000	-3.387313000	0.456591000
1	-0.601243000	-1.646242000	0.397120000
1	-2.235557000	-2.227922000	0.751689000
6	-3.794806000	3.942126000	4.113259000
1	-4.669010000	4.536106000	3.867969000
6	0.632748000	-2.944346000	2.620872000
1	0.753227000	-3.947620000	2.192947000
1	0.905990000	-2.997092000	3.678157000
1	1.341097000	-2.277889000	2.115277000
6	-1.095400000	5.159510000	0.358646000
1	-1.001552000	5.386476000	-0.711522000
1	-0.596450000	5.959456000	0.908031000
1	-2.162586000	5.195032000	0.596039000
6	4.456197000	2.513181000	2.790268000
1	5.381454000	2.667549000	2.220898000
1	4.384108000	3.316526000	3.531248000
1	3.614112000	2.597044000	2.100114000
6	-0.991724000	-2.222630000	5.671783000
1	-1.290516000	-3.082764000	5.083261000
6	2.072058000	5.222158000	1.955018000
1	2.837730000	5.869103000	2.400775000
1	1.551164000	5.809267000	1.193036000
1	2.587661000	4.396583000	1.455845000
6	-1.342465000	2.676438000	-0.056083000
1	-1.406458000	2.881780000	-1.132506000
1	-2.364401000	2.669947000	0.335601000
1	-0.912389000	1.678692000	0.073161000
6	3.713652000	-1.961777000	4.409737000
1	3.615355000	-2.945565000	4.884969000
1	4.782701000	-1.751092000	4.313760000
1	3.290425000	-2.034944000	3.403209000
6	0.341652000	5.896955000	3.703982000
1	1.055957000	6.632124000	4.096875000
1	-0.280148000	5.557534000	4.536367000
1	-0.303626000	6.413821000	2.986444000
6	3.624323000	-0.743789000	6.658805000
1	3.627847000	-1.716038000	7.168666000
1	3.059933000	-0.043506000	7.280680000

1	4.659740000	-0.393787000	6.606817000
6	-3.492908000	0.229194000	1.873318000
1	-4.567163000	0.441072000	1.947732000
1	-3.340969000	-0.390175000	0.983422000
1	-2.973796000	1.177478000	1.725500000
6	5.771586000	0.950082000	4.292967000
1	6.632633000	1.000967000	3.613258000
1	5.820722000	-0.009318000	4.809817000
1	5.906226000	1.743457000	5.033252000
6	-3.811149000	-1.750752000	3.448188000
1	-4.874477000	-1.500222000	3.553410000
1	-3.493800000	-2.231320000	4.378529000
1	-3.727547000	-2.478102000	2.633445000
6	1.027644000	2.446539000	5.788495000
7	1.895448000	-0.032442000	-0.331584000
1	2.609551000	0.158096000	-1.033065000
1	1.256222000	-0.802901000	-0.528299000
1	-0.406642000	2.404084000	-3.805871000
8	2.350776000	0.465999000	-4.435780000
6	2.818488000	0.559136000	-5.856716000
6	2.500129000	-0.712092000	-6.620426000
1	2.339842000	1.442503000	-6.283745000
1	3.891426000	0.740844000	-5.778429000
1	2.861568000	-0.605281000	-7.649236000
1	2.995215000	-1.578672000	-6.173203000
1	1.423621000	-0.902731000	-6.665322000
6	0.918037000	0.754344000	-4.116444000
6	0.651780000	2.246498000	-4.041430000
1	0.330436000	0.255834000	-4.889083000
1	0.749341000	0.251825000	-3.163242000
1	1.249432000	2.718863000	-3.256182000
1	0.857264000	2.747621000	-4.992051000
1	5.697866000	0.992029000	-0.567943000
8	3.984761000	0.261457000	-2.666329000
6	4.789535000	-1.007381000	-2.651660000
6	3.845261000	-2.194049000	-2.636873000
1	5.430235000	-0.990426000	-3.538341000
1	5.408984000	-0.968889000	-1.754343000
1	3.244928000	-2.214323000	-1.722802000
1	3.176367000	-2.192054000	-3.502494000
1	4.436094000	-3.115359000	-2.672004000
6	4.768299000	1.529696000	-2.463513000
6	5.056727000	1.769720000	-0.993955000
1	5.677425000	1.446177000	-3.066248000
1	4.127579000	2.306234000	-2.885042000
1	5.584245000	2.724739000	-0.891640000
1	4.133278000	1.826475000	-0.409094000
1	3.112404000	0.351040000	-3.594537000

$^{6}\mathbf{3}_{dd,RC}$:

26	0.160672000	-0.552405000	-1.461011000
15	-0.638235000	1.950595000	-1.055889000
15	2.419730000	-0.791653000	-0.096581000
15	-1.582940000	-2.155499000	-0.228211000
7	0.344215000	-0.934305000	-3.320833000
6	-0.905653000	-2.433889000	1.519983000
6	2.864520000	-2.460762000	0.812998000
1	1.884251000	-2.797457000	1.164482000
6	-0.265662000	-1.351666000	2.186323000
6	2.413553000	0.553960000	1.241175000
6	-1.566117000	2.572024000	-2.652200000
1	-0.745106000	2.493089000	-3.377508000
6	-1.537292000	0.967528000	1.478932000
6	1.164136000	2.001294000	2.744438000
1	0.223849000	2.311237000	3.189490000
6	-1.500049000	-3.894808000	-1.075539000
1	-2.088308000	-4.581952000	-0.457109000
5	-0.202473000	0.149607000	1.694121000
6	0.299389000	-1.606630000	3.460765000
1	0.813483000	-0.804505000	3.980877000
6	0.202110000	-2.859695000	4.075791000
1	0.632484000	-3.015868000	5.059392000
6	3.582251000	1.244369000	1.596844000
1	4.531716000	0.973532000	1.157769000
6	-0.433491000	-3.908855000	3.408085000
1	-0.504403000	-4.889868000	3.865986000
6	3.551566000	2.291274000	2.529133000
1	4.471196000	2.803037000	2.792576000
6	2.338408000	2.672359000	3.104350000
1	2.299976000	3.488735000	3.817815000
6	3.888164000	-0.471368000	-1.340572000
1	3.726360000	-1.305388000	-2.036981000
6	-2.485907000	0.856661000	2.525953000
1	-2.305066000	0.148701000	3.328813000
6	-1.818287000	1.889523000	0.427724000
6	0.672853000	3.341631000	-0.660659000
1	1.398530000	2.801539000	-0.046718000
6	-3.478779000	-1.858884000	0.147327000
1	-3.428354000	-1.113831000	0.948317000
6	-2.990713000	2.657994000	0.467414000
1	-3.211934000	3.354476000	-0.328966000
6	-3.642793000	1.643615000	2.565844000
1	-4.340428000	1.541112000	3.390343000
6	-2.125673000	-3.829325000	-2.481622000
1	-1.998475000	-4.800186000	-2.976949000
1	-1.631570000	-3.066953000	-3.094929000
1	-3.196894000	-3.614059000	-2.454035000
6	-3.896129000	2.546757000	1.532259000
1	-4.791620000	3.159151000	1.542955000
6	-0.048749000	-4.395740000	-1.150836000
1	-0.031859000	-5.394771000	-1.604652000
1	0.421003000	-4.468487000	-0.166330000
1	0.559258000	-3.733939000	-1.777226000
6	-2.097760000	4.017231000	-2.697922000
1	-2.344975000	4.261466000	-3.739349000
1	-1.373827000	4.756117000	-2.351122000
1	-3.016253000	4.140440000	-2.117100000
6	3.662118000	0.846538000	-2.100828000
1	4.439485000	0.966058000	-2.865916000
1	3.722955000	1.709218000	-1.428925000
1	2.690470000	0.868854000	-2.603141000
6	-0.972314000	-3.695366000	2.131666000
1	-1.437567000	-4.529592000	1.619385000
6	1.380468000	3.804662000	-1.947389000

1	2.263630000	4.396006000	-1.675981000
1	0.740418000	4.440559000	-2.565753000
1	1.720234000	2.964245000	-2.559719000
6	-2.658770000	1.574187000	-3.068639000
1	-3.025969000	1.833232000	-4.069788000
1	-3.513486000	1.612946000	-2.385301000
1	-2.279870000	0.548530000	-3.102957000
6	3.404774000	-3.500533000	-0.185829000
1	3.444661000	-4.480683000	0.304937000
1	4.418236000	-3.261124000	-0.521061000
1	2.766714000	-3.599330000	-1.069298000
6	0.131492000	4.520257000	0.163813000
1	0.961301000	5.198985000	0.400287000
1	-0.306131000	4.188773000	1.109156000
1	-0.624140000	5.098464000	-0.376972000
6	3.778908000	-2.295368000	2.036939000
1	3.891099000	-3.268734000	2.531705000
1	3.357997000	-1.596859000	2.764860000
1	4.779934000	-1.944475000	1.767568000
6	-4.225638000	-1.236612000	-1.041469000
1	-5.232722000	-0.943373000	-0.719083000
1	-4.340040000	-1.938692000	-1.873173000
1	-3.718859000	-0.344971000	-1.413141000
6	5.341216000	-0.566438000	-0.835658000
1	6.003106000	-0.666233000	-1.705672000
1	5.521317000	-1.425347000	-0.187320000
1	5.652708000	0.336923000	-0.304141000
6	-4.193408000	-3.109233000	0.682178000
1	-5.211976000	-2.834976000	0.985083000
1	-3.693251000	-3.531735000	1.558537000
1	-4.280029000	-3.890540000	-0.081116000
6	1.169125000	0.920331000	1.829525000
7	1.219573000	-1.346947000	-4.265195000
1	0.931249000	-1.340069000	-5.238987000
1	2.218707000	-1.343135000	-4.082915000
1	0.742076000	-5.945713000	-4.768830000
8	1.326216000	-3.882847000	-7.443800000
6	1.467021000	-4.633017000	-8.730564000
6	2.910422000	-4.657477000	-9.197452000
1	1.061523000	-5.631242000	-8.555459000
1	0.813154000	-4.102766000	-9.425088000
1	2.967829000	-5.214513000	-10.139207000
1	3.287374000	-3.646416000	-9.376178000
1	3.564056000	-5.156803000	-8.475916000
6	1.620706000	-4.566137000	-6.146216000
6	0.483026000	-5.478940000	-5.725806000
1	2.563009000	-5.098678000	-6.287124000
1	1.770356000	-3.745088000	-5.442814000
1	-0.446252000	-4.917718000	-5.589643000
1	0.309882000	-6.281466000	-6.448971000
1	-1.459978000	0.370949000	-6.837330000
8	0.370131000	-1.668182000	-7.411677000
6	1.061966000	-0.607682000	-8.215090000
6	2.509665000	-0.516173000	-7.773226000
1	0.958599000	-0.882586000	-9.269206000
1	0.521930000	0.321666000	-8.027630000
1	2.584937000	-0.211128000	-6.725364000
1	3.035601000	-1.466332000	-7.905246000
1	3.019808000	0.237085000	-8.382718000
6	-1.129429000	-1.677724000	-7.500015000
6	-1.739975000	-0.651247000	-6.564244000
1	-1.391526000	-1.506191000	-8.548200000
1	-1.405385000	-2.697142000	-7.224335000
1	-2.831746000	-0.722430000	-6.626638000
1	-1.443143000	-0.833648000	-5.526540000
1	0.889619000	-2.825467000	-7.440687000

$^{2}\mathbf{3}_{dp}$:

26	-0.169344000	-0.183537000	-1.321443000
15	-0.654403000	2.198752000	-0.749809000
15	2.169471000	-0.920980000	-0.357024000
15	-1.969040000	-1.544439000	-0.125792000
7	0.337523000	0.165324000	-3.064450000
6	-1.248072000	-2.158473000	1.511706000
6	2.394937000	-2.730101000	0.337440000
1	1.415403000	-2.915987000	0.789347000
6	-0.338619000	-1.305783000	2.191506000
6	2.507298000	0.246036000	1.104680000
6	-1.752935000	3.002342000	-2.141688000
1	-1.078960000	2.902070000	-3.003047000
6	-1.275742000	1.228776000	1.867967000
6	1.667597000	1.733344000	2.839327000
1	0.838274000	2.148120000	3.403494000
6	-2.311376000	-3.132069000	-1.196316000
1	-3.158704000	-3.648412000	-0.727396000
5	-0.071408000	0.206951000	1.829136000
6	0.283824000	-1.802514000	3.362274000
1	1.000771000	-1.178478000	3.886531000
6	-0.016205000	-3.071526000	3.870627000
1	0.467988000	-3.421022000	4.776455000
6	3.804026000	0.699773000	1.391079000
1	4.649243000	0.324107000	0.832778000
6	-0.933133000	-3.886817000	3.201690000
1	-1.173827000	-4.872677000	3.585349000
6	4.038966000	1.643901000	2.400531000
1	5.053550000	1.968687000	2.606075000
6	2.966611000	2.167896000	3.124015000
1	3.133982000	2.911605000	3.895960000
6	3.594152000	-0.651907000	-1.669550000
1	3.197352000	-1.226733000	-2.516240000
6	-2.058497000	1.193979000	3.047860000
1	-1.871286000	0.416863000	3.782287000
6	-1.568479000	2.239837000	0.908651000
6	0.892475000	3.373646000	-0.565184000
1	1.642016000	2.695741000	-0.145716000
6	-3.741646000	-0.880991000	0.394210000
1	-3.513115000	0.127133000	0.753029000
6	-2.580530000	3.176309000	1.167078000
1	-2.806671000	3.948389000	0.445998000
6	-3.056830000	2.141326000	3.302449000
1	-3.629490000	2.092169000	4.222605000
6	-2.685555000	-2.733437000	-2.640177000
1	-2.895080000	-3.643977000	-3.215261000
1	-1.851024000	-2.217096000	-3.128768000

1	-3.565575000	-2.093164000	-2.701375000
6	-3.316001000	3.137756000	2.360019000
1	-4.088183000	3.878452000	2.539153000
6	-1.089798000	-4.064720000	-1.241317000
1	-1.340859000	-4.956997000	-1.828200000
1	-0.753423000	-4.394120000	-0.256538000
1	-0.253756000	-3.565548000	-1.741375000
6	-2.129286000	4.493502000	-2.029460000
1	-2.429725000	4.850018000	-3.022873000
1	-1.307702000	5.125242000	-1.688024000
1	-2.983362000	4.651491000	-1.365164000
6	3.653612000	0.824755000	-2.096144000
1	4.337058000	0.927682000	-2.948226000
1	4.027459000	1.463614000	-1.289367000
1	2.672124000	1.189123000	-2.403337000
6	-1.538220000	-3.432435000	2.021586000
1	-2.237120000	-4.083022000	1.508023000
6	1.388464000	3.849096000	-1.943459000
1	2.380428000	4.302172000	-1.828133000
1	0.731255000	4.607513000	-2.378967000
1	1.484435000	3.026822000	-2.659113000
6	-2.998705000	2.140364000	-2.405397000
1	-3.551623000	2.550157000	-3.259481000
1	-3.671936000	2.139392000	-1.541505000
1	-2.736021000	1.104574000	-2.641444000
6	2.595068000	-3.737038000	-0.810868000
1	2.452650000	-4.754360000	-0.426324000
1	3.601486000	-3.685533000	-1.235075000
1	1.879041000	-3.590775000	-1.625487000
6	0.693167000	4.540579000	0.414775000
1	1.638201000	5.090865000	0.508813000
1	0.409965000	4.191467000	1.411236000
1	-0.068355000	5.248477000	0.072033000
6	3.461086000	-2.873251000	1.434570000
1	3.450111000	-3.904163000	1.811653000
1	3.264262000	-2.206567000	2.278519000
1	4.469968000	-2.666270000	1.065453000
6	-4.721100000	-0.774398000	-0.786343000
1	-5.609230000	-0.222174000	-0.454460000
1	-5.058735000	-1.761251000	-1.119333000
1	-4.301989000	-0.241016000	-1.642125000
6	5.004608000	-1.193376000	-1.364533000
1	5.596888000	-1.137821000	-2.287239000
1	5.007696000	-2.233752000	-1.037533000
1	5.531915000	-0.599653000	-0.613066000
6	-4.370372000	-1.688000000	1.541065000
1	-5.338680000	-1.240222000	1.798377000
1	-3.750821000	-1.687116000	2.440552000
1	-4.557862000	-2.728907000	1.250994000
6	1.405872000	0.750082000	1.853025000
7	0.828317000	-0.741283000	-3.933693000
1	0.061016000	1.013600000	-3.558888000
1	1.118151000	-1.574584000	-3.390249000

$^{4}\mathbf{3}_{dp}$:

26	-0.157604000	-0.247473000	-1.353466000
15	-0.526559000	2.262575000	-0.771542000
15	2.158201000	-0.908597000	-0.281599000
15	-1.951508000	-1.716036000	-0.068654000
7	-0.116350000	-0.364021000	-3.282547000
6	-1.168800000	-2.204389000	1.587539000
6	2.429634000	-2.700624000	0.441010000
1	1.449903000	-2.916186000	0.878779000
6	-0.329993000	-1.264068000	2.248569000
6	2.496302000	0.294683000	1.145337000
6	-1.485589000	3.139776000	-2.227154000
1	-0.733222000	3.028648000	-3.019595000
6	-1.307744000	1.256896000	1.796495000
6	1.630389000	1.785226000	2.861511000
1	0.794366000	2.196008000	3.419052000
6	-2.247653000	-3.389662000	-0.994229000
1	-2.828266000	-4.029057000	-0.319546000
5	-0.094872000	0.243941000	1.831216000
6	0.300306000	-1.675726000	3.448920000
1	0.964082000	-0.985876000	3.960468000
6	0.083487000	-2.942784000	4.001737000
1	0.571462000	-3.219988000	4.930304000
6	3.787141000	0.764220000	1.430941000
1	4.638650000	0.386914000	0.882735000
6	-0.746952000	-3.850752000	3.341386000
1	-0.914291000	-4.841724000	3.750264000
6	4.005875000	1.723219000	2.430443000
1	5.015030000	2.064535000	2.636120000
6	2.924025000	2.237564000	3.146745000
1	3.079458000	2.988952000	3.913840000
6	3.535206000	-0.689397000	-1.648932000
1	3.161493000	-1.393975000	-2.403715000
6	-2.152850000	1.217917000	2.933565000
1	-1.997989000	0.443783000	3.678879000
6	-1.554458000	2.270735000	0.824697000
6	0.998321000	3.433957000	-0.417772000
1	1.702829000	2.750693000	0.065376000
6	-3.726310000	-1.149077000	0.524185000
1	-3.479684000	-0.505488000	1.374924000
6	-2.593393000	3.191331000	1.024628000
1	-2.788540000	3.958615000	0.288987000
6	-3.173670000	2.154634000	3.132716000
1	-3.793250000	2.101236000	4.021750000
6	-3.060402000	-3.150469000	-2.280614000
1	-3.136447000	-4.095082000	-2.834585000
1	-2.572820000	-2.412419000	-2.928349000
1	-4.079359000	-2.813629000	-2.070806000
6	-3.395685000	3.143715000	2.173314000
1	-4.187961000	3.872914000	2.306640000
6	-0.907617000	-4.070891000	-1.312757000
1	-1.094948000	-5.027938000	-1.815660000
1	-0.318810000	-4.274972000	-0.414015000
1	-0.307066000	-3.451072000	-1.988096000
6	-1.826074000	4.638097000	-2.109818000
1	-2.091315000	5.010862000	-3.107897000
1	-0.997023000	5.245613000	-1.743608000
1	-2.691882000	4.816077000	-1.465492000
6	3.482401000	0.727740000	-2.244301000
1	4.186325000	0.798418000	-3.082815000

1	3.767398000	1.484022000	-1.505213000
1	2.488739000	0.982552000	-2.623597000
6	-1.358440000	-3.482950000	2.134731000
1	-1.977814000	-4.212374000	1.625564000
6	1.634360000	3.931817000	-1.728972000
1	2.605004000	4.390242000	-1.502327000
1	1.021933000	4.691895000	-2.222477000
1	1.811082000	3.121841000	-2.442769000
6	-2.726613000	2.332594000	-2.642076000
1	-3.125939000	2.742412000	-3.578969000
1	-3.518824000	2.407504000	-1.889955000
1	-2.495004000	1.277459000	-2.811739000
6	2.686310000	-3.712398000	-0.691351000
1	2.605426000	-4.729326000	-0.288156000
1	3.688153000	-3.608681000	-1.117948000
1	1.959111000	-3.620040000	-1.503636000
6	0.706892000	4.585183000	0.557980000
1	1.642859000	5.117757000	0.770896000
1	0.306756000	4.219872000	1.507399000
1	-0.001317000	5.312318000	0.148432000
6	3.480048000	-2.790919000	1.558992000
1	3.497764000	-3.816718000	1.949384000
1	3.244224000	-2.121089000	2.390211000
1	4.488484000	-2.553507000	1.206523000
6	-4.446948000	-0.296094000	-0.529451000
1	-5.373480000	0.104318000	-0.098531000
1	-4.719467000	-0.873258000	-1.418399000
1	-3.831042000	0.547041000	-0.846003000
6	4.989831000	-1.080368000	-1.322438000
1	5.544560000	-1.161259000	-2.266368000
1	5.078222000	-2.039203000	-0.809618000
1	5.497976000	-0.321200000	-0.721378000
6	-4.596115000	-2.311727000	1.024895000
1	-5.516364000	-1.905422000	1.463915000
1	-4.095530000	-2.900982000	1.799336000
1	-4.891759000	-2.983874000	0.211931000
6	1.383791000	0.796235000	1.878772000
7	-1.128697000	-0.705329000	-4.062419000
1	0.730676000	-0.030262000	-3.768538000
1	-0.892823000	-0.554810000	-5.062135000

$^{6}\mathbf{3}_{dp}$:

26	-0.211362000	-0.269353000	-1.428201000
15	-0.527210000	2.287036000	-0.761020000
15	2.130463000	-0.879283000	-0.284519000
15	-1.969137000	-1.729740000	-0.064926000
7	0.097518000	-0.461566000	-3.353321000
6	-1.179235000	-2.203060000	1.589643000
6	2.420635000	-2.670956000	0.434387000
1	1.444033000	-2.899258000	0.872624000
6	-0.336777000	-1.263729000	2.248282000
6	2.477559000	0.321964000	1.139699000
6	-1.489398000	3.192750000	-2.199011000
1	-0.755067000	3.070291000	-3.006436000
6	-1.322333000	1.250534000	1.795925000
6	1.619370000	1.786274000	2.881901000
1	0.786820000	2.184064000	3.453950000
6	-2.250119000	-3.403917000	-0.996413000
1	-2.833118000	-4.042075000	-0.322728000
5	-0.104874000	0.246834000	1.842508000
6	0.298283000	-1.679114000	3.445129000
1	0.965337000	-0.991087000	3.954612000
6	0.080489000	-2.945588000	3.998276000
1	0.571192000	-3.224130000	4.924888000
6	3.767859000	0.800424000	1.412456000
1	4.615324000	0.436874000	0.848982000
6	-0.755126000	-3.851002000	3.341145000
1	-0.924354000	-4.841275000	3.750620000
6	3.989939000	1.750633000	2.419499000
1	4.998248000	2.099638000	2.615938000
6	2.912662000	2.246410000	3.155838000
1	3.071323000	2.990375000	3.929414000
6	3.478100000	-0.660370000	-1.677762000
1	3.109967000	-1.391842000	-2.409106000
6	-2.180792000	1.197345000	2.922956000
1	-2.032260000	0.415923000	3.661773000
6	-1.561214000	2.273008000	0.830963000
6	1.011929000	3.434044000	-0.395783000
1	1.710726000	2.737617000	0.075613000
6	-3.743553000	-1.145124000	0.502874000
1	-3.501395000	-0.482226000	1.339787000
6	-2.605662000	3.187854000	1.028609000
1	-2.796291000	3.960383000	0.297704000
6	-3.205149000	2.130127000	3.120934000
1	-3.833775000	2.066905000	4.002839000
6	-3.055695000	-3.171770000	-2.288484000
1	-3.110590000	-4.115104000	-2.846447000
1	-2.574464000	-2.421450000	-2.926900000
1	-4.081619000	-2.852394000	-2.086754000
6	-3.418907000	3.128021000	2.168820000
1	-4.213615000	3.854693000	2.300887000
6	-0.905462000	-4.081146000	-1.304439000
1	-1.089015000	-5.049383000	-1.786516000
1	-0.312265000	-4.263587000	-0.404090000
1	-0.312165000	-3.471752000	-1.995385000
6	-1.789474000	4.698564000	-2.061591000
1	-2.064174000	5.084102000	-3.052094000
1	-0.939471000	5.283569000	-1.707629000
1	-2.638298000	4.893964000	-1.400098000
6	3.377234000	0.739883000	-2.304816000
1	4.062456000	0.807181000	-3.159202000
1	3.661677000	1.518749000	-1.589256000
1	2.367707000	0.953027000	-2.666855000
6	-1.369372000	-3.481833000	2.136541000
1	-1.991825000	-4.210091000	1.629669000
6	1.648267000	3.943432000	-1.702661000
1	2.622584000	4.391538000	-1.471294000
1	1.041101000	4.714109000	-2.185469000
1	1.817534000	3.141251000	-2.426346000
6	-2.756900000	2.417942000	-2.594730000
1	-3.184888000	2.871682000	-3.498404000
1	-3.518870000	2.474006000	-1.810014000
1	-2.538775000	1.369563000	-2.820878000
6	2.690503000	-3.678612000	-0.698426000

1	2.623473000	-4.696303000	-0.294668000
1	3.691144000	-3.561329000	-1.124423000
1	1.962342000	-3.597082000	-1.510978000
6	0.729394000	4.573759000	0.595997000
1	1.669113000	5.097843000	0.812772000
1	0.330184000	4.198177000	1.541749000
1	0.024059000	5.309744000	0.197918000
6	3.473461000	-2.749895000	1.551154000
1	3.502790000	-3.775709000	1.940650000
1	3.232171000	-2.083372000	2.383392000
1	4.478818000	-2.501704000	1.197021000
6	-4.459820000	-0.319330000	-0.575341000
1	-5.380902000	0.101261000	-0.152395000
1	-4.741662000	-0.923086000	-1.443029000
1	-3.840449000	0.508822000	-0.923568000
6	4.945514000	-1.008770000	-1.361244000
1	5.485564000	-1.119011000	-2.310582000
1	5.063450000	-1.942007000	-0.808463000
1	5.446482000	-0.212615000	-0.803499000
6	-4.611503000	-2.300226000	1.024526000
1	-5.535313000	-1.887001000	1.448883000
1	-4.113404000	-2.870287000	1.814748000
1	-4.900168000	-2.990915000	0.224697000
6	1.370000000	0.806241000	1.891278000
7	-1.180724000	-0.497908000	-3.873977000
1	0.892701000	-0.539618000	-3.988328000
1	-1.158153000	-0.619053000	-4.901958000

^{2}**3**$_{dp,RC}$:

26	-0.175470000	0.238698000	-0.814372000
15	-0.982415000	2.595085000	-0.118970000
15	2.166456000	0.031483000	0.053927000
15	-1.751440000	-1.566137000	0.408525000
7	0.220442000	-0.612567000	-2.417914000
6	-0.798753000	-1.939868000	2.019521000
6	2.715506000	-1.738931000	0.659572000
1	1.800248000	-2.113216000	1.125883000
6	-0.078415000	-0.898973000	2.671986000
6	2.371652000	1.200837000	1.529171000
6	-2.110370000	3.303279000	-1.547859000
1	-1.363763000	3.363288000	-2.350559000
6	-1.489670000	1.426814000	2.423426000
6	1.366266000	2.414984000	3.379917000
1	0.508821000	2.635715000	4.007846000
6	-1.873883000	-3.312269000	-0.439719000
1	-2.296670000	-3.979910000	0.318874000
5	-0.121770000	0.643433000	2.346555000
6	0.687513000	-1.240795000	3.815632000
1	1.265352000	-0.468260000	4.312598000
6	0.707526000	-2.539223000	4.334425000
1	1.291128000	-2.761134000	5.221551000
6	3.571178000	1.888671000	1.765407000
1	4.437439000	1.708919000	1.145202000
6	-0.013623000	-3.545634000	3.690208000
1	-0.002552000	-4.561554000	4.070725000
6	3.678269000	2.818416000	2.809578000
1	4.619565000	3.330804000	2.977516000
6	2.572181000	3.084444000	3.618113000
1	2.639759000	3.811341000	4.420544000
6	3.417436000	0.504665000	-1.363786000
1	3.163871000	-0.259077000	-2.111693000
6	-2.256349000	1.214093000	3.594986000
1	-1.932454000	0.464137000	4.309856000
6	-1.954184000	2.399149000	1.493550000
6	0.319568000	4.020099000	0.204851000
1	1.159592000	3.467318000	0.633311000
6	-3.547286000	-1.260784000	1.128408000
1	-3.343539000	-0.544168000	1.929698000
6	-3.128653000	3.118155000	1.758530000
1	-3.491881000	3.854870000	1.055792000
6	-3.415756000	1.951369000	3.861588000
1	-3.974337000	1.772673000	4.774254000
6	-2.833209000	-3.302907000	-1.643570000
1	-2.877611000	-4.314062000	-2.067176000
1	-2.483099000	-2.632630000	-2.434178000
1	-3.851485000	-3.017582000	-1.370457000
6	-3.854149000	2.903478000	2.939226000
1	-4.755921000	3.476618000	3.127063000
6	-0.486518000	-3.830773000	-0.849396000
1	-0.584890000	-4.843379000	-1.260862000
1	0.205279000	-3.882104000	-0.003917000
1	-0.045481000	-3.191833000	-1.619723000
6	-2.724247000	4.705835000	-1.366185000
1	-3.043146000	5.073413000	-2.350063000
1	-2.029658000	5.437290000	-0.952425000
1	-3.615169000	4.682461000	-0.732425000
6	3.068931000	1.883431000	-1.947226000
1	3.716188000	2.090066000	-2.808251000
1	3.227205000	2.680090000	-1.213009000
1	2.030511000	1.929009000	-2.289266000
6	-0.747633000	-3.245314000	2.534304000
1	-1.272993000	-4.052999000	2.040594000
6	0.788130000	4.663015000	-1.113638000
1	1.676553000	5.274182000	-0.912849000
1	0.030157000	5.322391000	-1.545698000
1	1.063630000	3.920029000	-1.867305000
6	-3.196590000	2.306082000	-1.979589000
1	-3.670876000	2.668395000	-2.900332000
1	-3.979975000	2.220437000	-1.220299000
1	-2.798151000	1.307958000	-2.181375000
6	3.049556000	-2.641770000	-0.541518000
1	3.162391000	-3.674671000	-0.190675000
1	3.988558000	-2.356874000	-1.025554000
1	2.254552000	-2.634424000	-1.293647000
6	-0.132799000	5.070671000	1.232009000
1	0.695400000	5.767895000	1.412839000
1	-0.400749000	4.616978000	2.189522000
1	-0.987814000	5.657482000	0.882872000
6	3.826104000	-1.730549000	1.721341000
1	3.988120000	-2.757847000	2.071758000
1	3.554484000	-1.121849000	2.587867000
1	4.778647000	-1.361436000	1.329458000
6	-4.479200000	-0.586563000	0.110983000
1	-5.410780000	-0.295425000	0.612544000

1	-4.746487000	-1.251886000	-0.715671000
1	-4.028064000	0.313921000	-0.307751000
6	4.930466000	0.382169000	-1.096254000
1	5.451585000	0.399336000	-2.061942000
1	5.207387000	-0.544395000	-0.590828000
1	5.317093000	1.221938000	-0.513014000
6	-4.179374000	-2.517412000	1.745717000
1	-5.110276000	-2.234872000	2.253748000
1	-3.528746000	-2.986800000	2.489692000
1	-4.437182000	-3.264675000	0.987167000
6	1.237876000	1.446670000	2.355462000
7	-0.657208000	-0.893036000	-3.406230000
1	1.175687000	-0.713418000	-2.758780000
1	-1.611495000	-0.808501000	-3.014802000
1	1.732122000	-2.650116000	-9.973244000
8	-0.276408000	-2.512899000	-7.199233000
6	0.352407000	-3.086106000	-5.971956000
6	-0.187480000	-4.475196000	-5.679467000
1	1.433847000	-3.081097000	-6.131631000
1	0.093252000	-2.370307000	-5.185476000
1	0.277470000	-4.850867000	-4.760774000
1	-1.270991000	-4.450866000	-5.529025000
1	0.040985000	-5.186011000	-6.479764000
6	0.165163000	-2.968072000	-8.546128000
6	1.459376000	-2.296236000	-8.972792000
1	0.253136000	-4.054990000	-8.495620000
1	-0.669737000	-2.712243000	-9.200861000
1	1.345321000	-1.208817000	-9.016470000
1	2.286440000	-2.537732000	-8.297970000
1	-2.378222000	2.113244000	-7.404853000
8	-1.620276000	-0.542770000	-7.011841000
6	-2.998721000	-0.696803000	-6.465750000
6	-3.687788000	-1.834242000	-7.196378000
1	-2.904221000	-0.878157000	-5.391149000
1	-3.504982000	0.255400000	-6.632421000
1	-3.745320000	-1.629639000	-8.269335000
1	-3.167887000	-2.784676000	-7.043812000
1	-4.707565000	-1.941624000	-6.812303000
6	-0.881837000	0.709341000	-6.669090000
6	-1.324830000	1.858317000	-7.555898000
1	-1.037397000	0.893744000	-5.602657000
1	0.166209000	0.450887000	-6.833098000
1	-0.730282000	2.746354000	-7.313883000
1	-1.168916000	1.617813000	-8.611407000
1	-0.950964000	-1.539161000	-7.104118000

$^{4}\mathbf{3}_{dp,RC}$:

26	-0.066073000	0.051900000	-0.750213000
15	-0.747615000	2.525384000	-0.280248000
15	2.276424000	-0.313938000	0.410149000
15	-1.803746000	-1.508962000	0.573220000
7	0.240393000	-0.072812000	-2.665481000
6	-0.960882000	-1.865898000	2.236679000
6	2.716117000	-2.054821000	1.167656000
1	1.767097000	-2.345173000	1.630179000
6	-0.217758000	-0.828598000	2.867333000
6	2.457669000	0.952570000	1.812314000
6	-1.782982000	3.216293000	-1.783503000
1	-1.013300000	3.159563000	-2.564709000
6	-1.444923000	1.562232000	2.325754000
6	1.398752000	2.437709000	3.422106000
1	0.509011000	2.797480000	3.929397000
6	-1.929334000	-3.242185000	-0.283362000
1	-2.395482000	-3.923079000	0.437176000
5	-0.133486000	0.685291000	2.417444000
6	0.453661000	-1.144109000	4.075255000
1	1.044884000	-0.377705000	4.565900000
6	0.367235000	-2.410908000	4.662691000
1	0.882209000	-2.612320000	5.596072000
6	3.692162000	1.541003000	2.124826000
1	4.594425000	1.214861000	1.626765000
6	-0.368162000	-3.414960000	4.029988000
1	-0.433943000	-4.406763000	4.464751000
6	3.787482000	2.558679000	3.085216000
1	4.755111000	2.992917000	3.313589000
6	2.636907000	3.012801000	3.731862000
1	2.696846000	3.809922000	4.465356000
6	3.647930000	0.048265000	-0.932216000
1	3.342245000	-0.658751000	-1.714915000
6	-2.290076000	1.489129000	3.461326000
1	-2.061218000	0.772149000	4.243765000
6	-1.788401000	2.496846000	1.306088000
6	0.637915000	3.874221000	0.019888000
1	1.417384000	3.292432000	0.520075000
6	-3.622565000	-1.100058000	1.162099000
1	-3.437113000	-0.374990000	1.960556000
6	-2.919599000	3.312114000	1.457832000
1	-3.186469000	4.020052000	0.686265000
6	-3.404945000	2.321788000	3.611605000
1	-4.022374000	2.246344000	4.500460000
6	-2.827370000	-3.178101000	-1.531230000
1	-2.788284000	-4.141285000	-2.053980000
1	-2.494585000	-2.404928000	-2.233756000
1	-3.872321000	-2.979105000	-1.281895000
6	-3.721667000	3.235361000	2.604945000
1	-4.586522000	3.883292000	2.700999000
6	-0.525057000	-3.760472000	-0.629383000
1	-0.601134000	-4.750440000	-1.095969000
1	0.106272000	-3.857271000	0.258046000
1	-0.020751000	-3.093376000	-1.338541000
6	-2.301694000	4.667041000	-1.739434000
1	-2.584014000	4.964374000	-2.757873000
1	-1.560794000	5.384183000	-1.383106000
1	-3.198624000	4.766102000	-1.121336000
6	3.489855000	1.476363000	-1.481652000
1	4.166545000	1.618269000	-2.333132000
1	3.743079000	2.226696000	-0.725325000
1	2.472242000	1.680358000	-1.825948000
6	-1.017107000	-3.142177000	2.817611000
1	-1.561561000	-3.944022000	2.333181000
6	1.205628000	4.398362000	-1.312247000
1	2.132113000	4.950281000	-1.111819000
1	0.517309000	5.086827000	-1.810397000
1	1.446888000	3.593716000	-2.013235000

6	-2.917067000	2.252155000	-2.167909000
1	-3.336854000	2.554269000	-3.135830000
1	-3.729259000	2.284480000	-1.434795000
1	-2.570360000	1.218576000	-2.259489000
6	3.035785000	-3.064469000	0.048906000
1	3.042440000	-4.077223000	0.470030000
1	4.019706000	-2.889842000	-0.395768000
1	2.291737000	-3.042808000	-0.753278000
6	0.216603000	5.017310000	0.956105000
1	1.081347000	5.669382000	1.134511000
1	-0.125312000	4.643265000	1.924309000
1	-0.580096000	5.633745000	0.528200000
6	3.790814000	-2.032174000	2.265787000
1	3.884450000	-3.038733000	2.693672000
1	3.526565000	-1.348561000	3.077291000
1	4.774622000	-1.743720000	1.883564000
6	-4.449906000	-0.407930000	0.069750000
1	-5.405104000	-0.076929000	0.496327000
1	-4.677404000	-1.074664000	-0.767490000
1	-3.934296000	0.470048000	-0.321417000
6	5.124122000	-0.236751000	-0.595156000
1	5.702603000	-0.200278000	-1.527522000
1	5.283117000	-1.219347000	-0.149735000
1	5.551788000	0.515700000	0.073127000
6	-4.348133000	-2.314880000	1.760411000
1	-5.305067000	-1.984345000	2.183887000
1	-3.776470000	-2.783174000	2.567155000
1	-4.572016000	-3.075971000	1.004821000
6	1.278523000	1.385859000	2.482217000
7	-0.332285000	-0.718657000	-3.674364000
1	0.983621000	0.530156000	-3.034019000
1	-1.072383000	-1.322091000	-3.278294000
1	1.165215000	-0.888594000	-10.672457000
8	-0.021036000	-1.862499000	-7.612613000
6	1.030579000	-2.109119000	-6.583114000
6	1.209224000	-3.596609000	-6.332473000
1	1.944871000	-1.630559000	-6.943228000
1	0.665632000	-1.580808000	-5.696707000
1	1.970461000	-3.738954000	-5.556761000
1	0.276255000	-4.050006000	-5.983516000
1	1.544902000	-4.129432000	-7.227812000
6	0.300275000	-2.003462000	-9.057754000
6	0.958133000	-0.748749000	-9.605685000
1	0.933874000	-2.887566000	-9.154812000
1	-0.662451000	-2.206731000	-9.530564000
1	0.302145000	0.120189000	-9.496363000
1	1.908804000	-0.537916000	-9.106072000
1	-4.399380000	0.631334000	-6.896427000
8	-2.161670000	-1.050268000	-6.944765000
6	-3.160897000	-2.026678000	-6.426116000
6	-4.028492000	-2.548340000	-7.557151000
1	-2.579394000	-2.821560000	-5.952326000
1	-3.738603000	-1.506674000	-5.658543000
1	-4.604471000	-1.743157000	-8.021608000
1	-3.418987000	-3.032475000	-8.326464000
1	-4.730517000	-3.291684000	-7.163004000
6	-2.263285000	0.382608000	-6.552637000
6	-3.445831000	1.060055000	-7.220272000
1	-2.308642000	0.416644000	-5.460652000
1	-1.312007000	0.807238000	-6.879451000
1	-3.449359000	2.121402000	-6.947808000
1	-3.376349000	0.985526000	-8.309340000
1	-1.082737000	-1.463545000	-7.283490000

$^{6}\mathbf{3}_{dp,RC}$:

26	-0.578055000	0.198251000	-0.842813000
15	-0.787589000	2.736689000	-0.132634000
15	1.827673000	-0.525038000	-0.037752000
15	-2.309514000	-1.125191000	0.692988000
7	-0.684337000	0.268946000	-2.822411000
6	-1.365281000	-1.663757000	2.246241000
6	2.112333000	-2.337883000	0.627882000
1	1.188028000	-2.525103000	1.184289000
6	-0.344968000	-0.820150000	2.767213000
6	2.394050000	0.654059000	1.332173000
6	-1.941202000	3.612487000	-1.438803000
1	-1.322544000	3.498048000	-2.339163000
6	-1.247857000	1.729790000	2.506583000
6	1.822534000	2.158508000	3.155499000
1	1.085718000	2.590171000	3.825548000
6	-2.784949000	-2.777317000	-0.208861000
1	-3.486957000	-3.308344000	0.445326000
5	-0.076465000	0.681743000	2.365193000
6	0.419985000	-1.308490000	3.856157000
1	1.219477000	-0.694109000	4.257539000
6	0.157809000	-2.550058000	4.445392000
1	0.752557000	-2.886992000	5.287717000
6	3.726120000	1.082178000	1.433135000
1	4.480274000	0.685750000	0.768476000
6	-0.865571000	-3.353122000	3.936997000
1	-1.080445000	-4.318564000	4.382547000
6	4.111180000	2.027056000	2.395073000
1	5.148986000	2.336567000	2.458224000
6	3.156582000	2.569350000	3.257063000
1	3.441238000	3.310550000	3.996255000
6	2.986442000	-0.343498000	-1.593381000
1	2.498289000	-1.052327000	-2.274640000
6	-1.938003000	1.697699000	3.744045000
1	-1.709688000	0.910029000	4.454952000
6	-1.593069000	2.757571000	1.582521000
6	0.794822000	3.874287000	-0.001410000
1	1.544374000	3.179291000	0.388368000
6	-3.971173000	-0.394139000	1.419450000
1	-3.611662000	0.511309000	1.917147000
6	-2.571855000	3.703036000	1.922809000
1	-2.840797000	4.483977000	1.226333000
6	-2.897551000	2.658434000	4.082057000
1	-3.397010000	2.611452000	5.043925000
6	-3.470602000	-2.480137000	-1.557556000
1	-3.716877000	-3.431620000	-2.045314000
1	-2.799893000	-1.926661000	-2.224965000
1	-4.396648000	-1.913278000	-1.453955000
6	-3.215630000	3.663984000	3.167620000
1	-3.962164000	4.412875000	3.409980000

6	-1.539143000	-3.646628000	-0.446413000
1	-1.835033000	-4.573444000	-0.953118000
1	-1.021719000	-3.918225000	0.475976000
1	-0.828152000	-3.125171000	-1.097124000
6	-2.248070000	5.114633000	-1.279071000
1	-2.634186000	5.490073000	-2.235454000
1	-1.371948000	5.711969000	-1.022254000
1	-3.022703000	5.302046000	-0.530483000
6	2.852423000	1.065079000	-2.195526000
1	3.444194000	1.123106000	-3.117861000
1	3.229780000	1.832303000	-1.510925000
1	1.812797000	1.293238000	-2.446553000
6	-1.612492000	-2.913546000	2.835288000
1	-2.389005000	-3.559816000	2.442312000
6	1.247827000	4.345042000	-1.395403000
1	2.247984000	4.787755000	-1.313979000
1	0.584735000	5.110894000	-1.808313000
1	1.308930000	3.521234000	-2.112552000
6	-3.236647000	2.807974000	-1.641971000
1	-3.828144000	3.269163000	-2.442637000
1	-3.851085000	2.803948000	-0.735711000
1	-3.033817000	1.772033000	-1.931610000
6	2.190469000	-3.338876000	-0.539281000
1	2.113296000	-4.358358000	-0.142648000
1	3.139842000	-3.267289000	-1.077993000
1	1.379500000	-3.203247000	-1.261576000
6	0.657846000	5.038554000	0.992314000
1	1.616428000	5.570016000	1.051025000
1	0.409251000	4.688575000	1.997633000
1	-0.103425000	5.762433000	0.685858000
6	3.293087000	-2.484614000	1.600460000
1	3.302412000	-3.508864000	1.994578000
1	3.208309000	-1.801193000	2.449414000
1	4.258588000	-2.308994000	1.115772000
6	-4.978454000	0.009818000	0.329446000
1	-5.724951000	0.682143000	0.770037000
1	-5.517482000	-0.858664000	-0.061052000
1	-4.509808000	0.533462000	-0.506917000
6	4.467012000	-0.749596000	-1.463362000
1	4.875552000	-0.892366000	-2.472126000
1	4.616809000	-1.680081000	-0.913702000
1	5.066353000	0.031267000	-0.987107000
6	-4.623567000	-1.310941000	2.464864000
1	-5.509622000	-0.808578000	2.873376000
1	-3.953565000	-1.534296000	3.298652000
1	-4.959793000	-2.256796000	2.023979000
6	1.410713000	1.182235000	2.216184000
7	-0.574253000	-0.843500000	-3.642170000
1	-0.848377000	1.105733000	-3.377796000
1	-0.397196000	-1.655718000	-3.023797000
1	1.269330000	0.073113000	-10.501926000
8	0.801071000	-1.379169000	-7.434710000
6	1.647849000	-0.786549000	-6.361118000
6	2.830611000	-1.684647000	-6.039405000
1	1.955606000	0.207122000	-6.698349000
1	0.963195000	-0.691509000	-5.511720000
1	3.407618000	-1.236133000	-5.222375000
1	2.492532000	-2.674568000	-5.717574000
1	3.505080000	-1.804611000	-6.893077000
6	1.240189000	-1.297302000	-8.851434000
6	0.945791000	0.066201000	-9.455138000
1	2.304675000	-1.541451000	-8.869505000
1	0.688399000	-2.096701000	-9.349735000
1	-0.125814000	0.286550000	-9.428410000
1	1.482819000	0.866016000	-8.935642000
1	-4.198884000	-2.136179000	-6.549181000
8	-1.424368000	-2.049872000	-6.880597000
6	-1.533952000	-3.339438000	-6.133501000
6	-1.960405000	-4.451374000	-7.072945000
1	-0.544321000	-3.515968000	-5.705863000
1	-2.243231000	-3.171221000	-5.320902000
1	-2.951212000	-4.260342000	-7.494382000
1	-1.246307000	-4.564764000	-7.894347000
1	-1.997262000	-5.397515000	-6.521248000
6	-2.377423000	-0.943112000	-6.551758000
6	-3.773884000	-1.260537000	-7.049893000
1	-2.311452000	-0.792000000	-5.471033000
1	-1.949625000	-0.078612000	-7.064093000
1	-4.428171000	-0.407523000	-6.837231000
1	-3.777340000	-1.434671000	-8.129774000
1	-0.340771000	-1.719505000	-7.166339000

^{2}**TS1**$_{d}$:

26	0.276697000	-0.437917000	-1.327778000
15	0.345686000	2.019427000	-1.163096000
15	2.098610000	-1.552084000	-0.083218000
15	-2.125337000	-1.270458000	-1.111359000
7	0.696452000	-0.766113000	-3.052763000
6	-1.952182000	-2.039496000	0.610404000
6	1.712467000	-3.365694000	0.533011000
1	0.632074000	-3.310390000	0.699813000
6	-1.054638000	-1.357263000	1.481609000
6	2.290420000	-0.515499000	1.484837000
6	0.059290000	3.077738000	-2.788432000
1	0.974573000	2.816291000	-3.338155000
6	-1.225123000	1.318153000	1.021024000
6	1.217564000	1.084436000	2.940150000
1	0.344002000	1.661605000	3.231578000
6	-2.578465000	-2.786467000	-2.239309000
1	-3.476454000	-3.259108000	-1.821903000
5	-0.287092000	-0.001182000	1.041505000
6	-0.887870000	-1.923413000	2.772577000
1	-0.211573000	-1.436850000	3.470005000
6	-1.558238000	-3.083710000	3.179381000
1	-1.397934000	-3.481575000	4.178539000
6	3.467951000	-0.398953000	2.240409000
1	4.344648000	-0.981398000	1.985514000
6	-2.432156000	-3.732905000	2.299132000
1	-2.950443000	-4.638827000	2.600285000
6	3.531632000	0.476809000	3.334166000
1	4.452082000	0.571095000	3.903345000
6	2.399983000	1.224849000	3.678533000
1	2.436081000	1.909303000	4.522610000
6	3.829128000	-1.714776000	-0.976190000

1	3.547382000	-2.322677000	-1.847514000
6	-2.267997000	1.496179000	1.964475000
1	-2.467920000	0.698621000	2.675984000
6	-1.005065000	2.390950000	0.109688000
6	1.957095000	2.851307000	-0.403570000
1	2.342356000	2.032407000	0.212427000
6	-3.844710000	-0.342652000	-0.889279000
1	-3.678477000	0.183900000	0.057152000
6	-1.769743000	3.566848000	0.171161000
1	-1.576665000	4.384873000	-0.511771000
6	-3.048084000	2.659352000	2.012887000
1	-3.842094000	2.752457000	2.749881000
6	-2.885506000	-2.321005000	-3.674388000
1	-3.087894000	-3.192408000	-4.312064000
1	-2.030564000	-1.783711000	-4.100709000
1	-3.761189000	-1.667023000	-3.721720000
6	-2.798535000	3.703310000	1.114761000
1	-3.392603000	4.612444000	1.144675000
6	-1.430582000	-3.808752000	-2.235894000
1	-1.688724000	-4.664329000	-2.875336000
1	-1.223713000	-4.188475000	-1.230853000
1	-0.512230000	-3.356031000	-2.625317000
6	-0.010597000	4.614894000	-2.707252000
1	0.095529000	5.035797000	-3.717615000
1	0.775158000	5.050207000	-2.086301000
1	-0.977029000	4.955304000	-2.322650000
6	4.268416000	-0.333948000	-1.488371000
1	5.175712000	-0.428453000	-2.100336000
1	4.493845000	0.344252000	-0.656966000
1	3.484787000	0.122950000	-2.098715000
6	-2.627503000	-3.201968000	1.015887000
1	-3.296316000	-3.716056000	0.331902000
6	3.005308000	3.168623000	-1.485375000
1	3.969599000	3.392528000	-1.009171000
1	2.727549000	4.042703000	-2.083925000
1	3.162161000	2.326753000	-2.166728000
6	-1.137017000	2.533050000	-3.585167000
1	-1.141429000	2.960101000	-4.598114000
1	-2.082557000	2.814362000	-3.108799000
1	-1.103139000	1.442739000	-3.666119000
6	1.974881000	-4.395810000	-0.580013000
1	1.555121000	-5.368144000	-0.289176000
1	3.044911000	-4.543306000	-0.761294000
1	1.507201000	-4.101437000	-1.525155000
6	1.711267000	4.056268000	0.518359000
1	2.661925000	4.370288000	0.972833000
1	1.018719000	3.806018000	1.325981000
1	1.303754000	4.918574000	-0.020822000
6	2.368408000	-3.766582000	1.863589000
1	1.992756000	-4.751972000	2.173481000
1	2.129323000	-3.051920000	2.655782000
1	3.458544000	-3.837775000	1.789450000
6	-4.090491000	0.711860000	-1.979276000
1	-4.991510000	1.292025000	-1.736436000
1	-4.248158000	0.260354000	-2.965272000
1	-3.251948000	1.406726000	-2.052774000
6	4.993986000	-2.427694000	-0.263959000

1	5.806766000	-2.591640000	-0.985833000
1	4.715046000	-3.402473000	0.141951000
1	5.407181000	-1.824224000	0.549734000
6	-5.052877000	-1.276983000	-0.725870000
1	-5.943032000	-0.684282000	-0.472734000
1	-4.903768000	-2.006293000	0.076055000
1	-5.277533000	-1.821220000	-1.650970000
6	1.114742000	0.213805000	1.826493000
7	0.961093000	-0.954130000	-4.205899000
1	3.477295000	2.312475000	-5.163191000
8	1.818724000	-0.222150000	-6.713895000
6	0.829467000	-0.476342000	-7.829282000
6	-0.601903000	-0.244084000	-7.386998000
1	1.153614000	0.189237000	-8.629636000
1	1.033364000	-1.511491000	-8.097654000
1	-1.267870000	-0.518027000	-8.212758000
1	-0.850956000	-0.861831000	-6.520146000
1	-0.790462000	0.804558000	-7.139385000
6	1.971445000	1.173936000	-6.157781000
6	3.353874000	1.296622000	-5.554685000
1	1.826629000	1.820883000	-7.023615000
1	1.173002000	1.306307000	-5.428689000
1	3.480645000	0.596349000	-4.724736000
1	4.131263000	1.117334000	-6.302013000
1	2.285222000	-4.323373000	-3.970924000
8	2.350851000	-2.803998000	-6.250624000
6	3.394593000	-3.329577000	-7.143149000
6	4.446368000	-2.254588000	-7.364735000
1	3.847223000	-4.216196000	-6.684893000
1	2.928462000	-3.636639000	-8.090442000
1	4.019529000	-1.371813000	-7.848560000
1	4.889617000	-1.947661000	-6.411799000
1	5.245304000	-2.646131000	-8.004201000
6	1.338947000	-3.795786000	-5.846503000
6	1.872244000	-4.823185000	-4.852951000
1	0.556014000	-3.191149000	-5.387319000
1	0.943006000	-4.282920000	-6.748959000
1	1.052855000	-5.473392000	-4.524685000
1	2.648026000	-5.462911000	-5.285961000
1	1.840571000	-0.965749000	-6.018808000

^{6}**TS1**$_{d}$:

26	0.424757000	-0.422580000	-1.521914000
15	0.518769000	2.086710000	-1.034920000
15	2.022444000	-1.661864000	0.059705000
15	-2.124627000	-1.370573000	-1.035386000
7	0.876395000	-0.748910000	-3.475567000
6	-2.003083000	-2.005502000	0.749790000
6	1.589552000	-3.443678000	0.728932000
1	0.505768000	-3.368770000	0.865002000
6	-1.126173000	-1.316462000	1.652316000
6	2.192621000	-0.551463000	1.583389000
6	0.257049000	3.151174000	-2.659495000
1	1.124277000	2.807576000	-3.240646000
6	-1.278725000	1.351000000	1.057823000
6	1.284287000	1.254638000	2.923907000
1	0.454460000	1.902781000	3.193094000

6	-2.561228000	-2.970423000	-2.056721000
1	-3.459931000	-3.421403000	-1.618830000
5	-0.407247000	0.068481000	1.366223000
6	-0.948623000	-1.920924000	2.930537000
1	-0.280354000	-1.435483000	3.636543000
6	-1.611170000	-3.085940000	3.321393000
1	-1.450971000	-3.496036000	4.315360000
6	3.409592000	-0.412415000	2.276318000
1	4.246395000	-1.055792000	2.037593000
6	-2.490698000	-3.719004000	2.430998000
1	-3.019918000	-4.622201000	2.720673000
6	3.573400000	0.552548000	3.277860000
1	4.521616000	0.638912000	3.800654000
6	2.502565000	1.403219000	3.590435000
1	2.614848000	2.164563000	4.358186000
6	3.787469000	-1.881589000	-0.766168000
1	3.510500000	-2.498116000	-1.633193000
6	-2.489741000	1.541589000	1.784665000
1	-2.810430000	0.749119000	2.456010000
6	-0.907226000	2.415919000	0.165778000
6	2.114106000	2.871452000	-0.221322000
1	2.435295000	2.068722000	0.448888000
6	-3.829197000	-0.401798000	-0.975582000
1	-3.694762000	0.212633000	-0.078911000
6	-1.670584000	3.596506000	0.106482000
1	-1.365758000	4.407431000	-0.542470000
6	-3.254353000	2.706520000	1.701143000
1	-4.160940000	2.806905000	2.292528000
6	-2.858242000	-2.612845000	-3.525221000
1	-3.027617000	-3.533930000	-4.099792000
1	-2.014656000	-2.083527000	-3.985290000
1	-3.753288000	-1.992974000	-3.630962000
6	-2.835420000	3.754720000	0.867304000
1	-3.406068000	4.676727000	0.804683000
6	-1.404451000	-3.978792000	-1.973890000
1	-1.652394000	-4.882871000	-2.546817000
1	-1.194324000	-4.278349000	-0.943422000
1	-0.489248000	-3.551471000	-2.399350000
6	0.312807000	4.690190000	-2.618599000
1	0.370240000	5.070211000	-3.648753000
1	1.181800000	5.073609000	-2.080355000
1	-0.584749000	5.126179000	-2.170343000
6	4.270668000	-0.520277000	-1.295117000
1	5.195045000	-0.647892000	-1.875012000
1	4.483712000	0.171930000	-0.472774000
1	3.521481000	-0.059876000	-1.947000000
6	-2.671996000	-3.176068000	1.152490000
1	-3.331604000	-3.692486000	0.462091000
6	3.221821000	3.097673000	-1.266434000
1	4.170016000	3.310872000	-0.755071000
1	3.005503000	3.949470000	-1.919284000
1	3.376439000	2.216490000	-1.896968000
6	-1.012313000	2.676326000	-3.386814000
1	-1.060324000	3.121055000	-4.390446000
1	-1.913005000	2.985384000	-2.844956000
1	-1.030750000	1.587105000	-3.493641000
6	1.874394000	-4.516063000	-0.338186000
1	1.421385000	-5.469060000	-0.033959000
1	2.947595000	-4.691342000	-0.467434000
1	1.455664000	-4.245479000	-1.312755000
6	1.860081000	4.122771000	0.633418000
1	2.792090000	4.418794000	1.135166000
1	1.109407000	3.930328000	1.404562000
1	1.521799000	4.975854000	0.035945000
6	2.207271000	-3.802437000	2.089830000
1	1.805070000	-4.766910000	2.430507000
1	1.965194000	-3.051555000	2.846680000
1	3.297218000	-3.898167000	2.042998000
6	-4.005752000	0.545978000	-2.172798000
1	-4.885663000	1.183948000	-2.012159000
1	-4.161850000	0.004035000	-3.111669000
1	-3.136634000	1.195247000	-2.298048000
6	4.921526000	-2.614152000	-0.024468000
1	5.727266000	-2.835612000	-0.738797000
1	4.602061000	-3.561633000	0.414097000
1	5.360522000	-2.002030000	0.768725000
6	-5.060047000	-1.298351000	-0.772642000
1	-5.949610000	-0.672065000	-0.617518000
1	-4.953971000	-1.945832000	0.103370000
1	-5.256046000	-1.930413000	-1.646911000
6	1.063003000	0.264799000	1.925636000
7	0.640860000	-0.993324000	-4.632807000
1	2.920722000	2.429095000	-5.459848000
8	1.616452000	-0.228000000	-7.139323000
6	0.653104000	-0.583989000	-8.251047000
6	-0.793821000	-0.446636000	-7.818276000
1	0.929766000	0.078564000	-9.071791000
1	0.933622000	-1.609493000	-8.487753000
1	-1.434636000	-0.844049000	-8.613039000
1	-0.982921000	-1.011118000	-6.900408000
1	-1.075006000	0.597730000	-7.653466000
6	1.606138000	1.170412000	-6.572900000
6	2.903353000	1.396540000	-5.826172000
1	1.506021000	1.808067000	-7.452170000
1	0.728800000	1.249440000	-5.931883000
1	2.979771000	0.730484000	-4.962157000
1	3.768146000	1.250201000	-6.478830000
1	2.362209000	-3.637018000	-3.942725000
8	2.404840000	-2.637708000	-6.497365000
6	3.557511000	-3.097427000	-7.290801000
6	4.454582000	-1.913154000	-7.611629000
1	4.108077000	-3.848397000	-6.713165000
1	3.182355000	-3.576601000	-8.205518000
1	3.928742000	-1.163314000	-8.208752000
1	4.810520000	-1.437736000	-6.691913000
1	5.326377000	-2.256432000	-8.179595000
6	1.571230000	-3.726108000	-5.952867000
6	2.197798000	-4.381644000	-4.727675000
1	0.633359000	-3.237484000	-5.688006000
1	1.387557000	-4.456801000	-6.752239000
1	1.521803000	-5.150682000	-4.335647000
1	3.153100000	-4.865649000	-4.956479000
1	1.740226000	-0.988682000	-6.452356000

^{2}**TS2**$_{d}$:

26	0.405907000	-0.566150000	-1.173207000
15	0.271194000	1.946768000	-1.196790000
15	2.324960000	-1.418113000	0.241103000
15	-1.966123000	-1.553636000	-0.798841000
7	0.812858000	-1.116554000	-2.861224000
6	-1.694962000	-2.131839000	0.977580000
6	2.087158000	-3.205894000	0.972394000
1	1.007283000	-3.228799000	1.150270000
6	-0.844475000	-1.308767000	1.765009000
6	2.405677000	-0.222633000	1.693863000
6	-0.090631000	2.734985000	-2.943196000
1	0.860671000	2.517501000	-3.447314000
6	-1.242968000	1.293811000	1.046305000
6	1.210324000	1.445474000	2.966801000
1	0.299696000	1.994998000	3.186436000
6	-2.283483000	-3.186076000	-1.791509000
1	-3.133123000	-3.692839000	-1.318314000
5	-0.203111000	0.071670000	1.224754000
6	-0.618026000	-1.729015000	3.099538000
1	0.028292000	-1.130521000	3.734207000
6	-1.199141000	-2.888475000	3.627476000
1	-1.000543000	-3.173786000	4.656692000
6	3.560248000	0.040034000	2.447678000
1	4.476764000	-0.505349000	2.260215000
6	-2.028815000	-3.681562000	2.827597000
1	-2.476233000	-4.588016000	3.223528000
6	3.546431000	1.017656000	3.452079000
1	4.447028000	1.222007000	4.022813000
6	2.367204000	1.725609000	3.705583000
1	2.345966000	2.489648000	4.477575000
6	4.040312000	-1.444669000	-0.674971000
1	3.808368000	-2.085693000	-1.536197000
6	-2.286236000	1.473292000	1.986856000
1	-2.409798000	0.739646000	2.778748000
6	-1.124797000	2.277746000	0.027804000
6	1.789161000	2.985270000	-0.523327000
1	2.250716000	2.273885000	0.169421000
6	-3.729657000	-0.719240000	-0.655384000
1	-3.614375000	-0.130906000	0.260978000
6	-1.995647000	3.377235000	-0.026277000
1	-1.887326000	4.129369000	-0.796923000
6	-3.164610000	2.562928000	1.928216000
1	-3.955268000	2.663437000	2.666359000
6	-2.649938000	-2.866276000	-3.251722000
1	-2.771244000	-3.800837000	-3.814650000
1	-1.861521000	-2.280345000	-3.737480000
1	-3.587445000	-2.308832000	-3.333809000
6	-3.021421000	3.519557000	0.917874000
1	-3.696039000	4.368299000	0.861605000
6	-1.053578000	-4.104441000	-1.716973000
1	-1.245669000	-5.029572000	-2.275575000
1	-0.808001000	-4.376556000	-0.686459000
1	-0.176119000	-3.618136000	-2.157362000
6	-0.336945000	4.253359000	-3.027369000
1	-0.252164000	4.569920000	-4.075806000
1	0.377368000	4.841232000	-2.447363000
1	-1.346134000	4.515963000	-2.696279000
6	4.353621000	-0.034596000	-1.199011000
1	5.250988000	-0.060060000	-1.830292000
1	4.542370000	0.663368000	-0.375808000
1	3.524529000	0.357708000	-1.793578000
6	-2.275243000	-3.297510000	1.502587000
1	-2.909268000	-3.925373000	0.884577000
6	2.805282000	3.309414000	-1.633335000
1	3.729162000	3.687211000	-1.176944000
1	2.437577000	4.083461000	-2.314413000
1	3.069950000	2.430790000	-2.229297000
6	-1.199493000	1.970102000	-3.683674000
1	-1.210725000	2.271317000	-4.739685000
1	-2.184210000	2.203765000	-3.266705000
1	-1.050258000	0.887888000	-3.635357000
6	2.420544000	-4.273804000	-0.085373000
1	2.088461000	-5.256463000	0.272721000
1	3.495581000	-4.342504000	-0.279755000
1	1.914192000	-4.077440000	-1.036059000
6	1.404191000	4.240895000	0.275862000
1	2.314669000	4.703440000	0.680208000
1	0.749195000	3.998827000	1.116411000
1	0.899673000	4.990801000	-0.342915000
6	2.792891000	-3.459820000	2.313333000
1	2.508263000	-4.451724000	2.689126000
1	2.501635000	-2.720482000	3.064839000
1	3.883519000	-3.441032000	2.222085000
6	-4.009071000	0.251980000	-1.811455000
1	-4.960426000	0.770293000	-1.633524000
1	-4.088213000	-0.260476000	-2.776264000
1	-3.223123000	1.005625000	-1.886578000
6	5.259728000	-2.044391000	0.048819000
1	6.088672000	-2.125079000	-0.667541000
1	5.073571000	-3.044183000	0.446325000
1	5.606891000	-1.408931000	0.868697000
6	-4.874533000	-1.722771000	-0.453193000
1	-5.801333000	-1.173909000	-0.239995000
1	-4.689606000	-2.395171000	0.389990000
1	-5.053993000	-2.328389000	-1.348508000
6	1.186691000	0.463432000	1.947955000
7	1.061194000	-1.557033000	-4.020705000
1	5.247923000	-0.146648000	-4.653127000
8	2.147659000	-0.051992000	-6.113529000
6	2.099409000	0.404573000	-7.551100000
6	1.398417000	1.743282000	-7.646835000
1	3.137354000	0.435172000	-7.887014000
1	1.568392000	-0.402891000	-8.050662000
1	1.370592000	2.048503000	-8.699017000
1	0.370080000	1.677298000	-7.281061000
1	1.925219000	2.522176000	-7.087363000
6	3.251853000	0.388313000	-5.190994000
6	4.466770000	-0.506257000	-5.331928000
1	3.440767000	1.425762000	-5.467495000
1	2.798004000	0.341282000	-4.203859000
1	4.227167000	-1.537797000	-5.058740000
1	4.873298000	-0.489485000	-6.347760000
1	-1.080117000	-0.750708000	-7.768395000

8	0.925700000	-2.339054000	-6.858583000
6	1.881107000	-3.391116000	-7.252160000
6	1.982242000	-4.519578000	-6.231876000
1	1.586087000	-3.771352000	-8.238650000
1	2.836290000	-2.870147000	-7.355441000
1	2.289998000	-4.134690000	-5.253621000
1	1.032926000	-5.053006000	-6.116762000
1	2.733834000	-5.246563000	-6.559998000
6	-0.494445000	-2.699536000	-7.018744000
6	-1.326848000	-1.430393000	-6.946438000
1	-0.614304000	-3.199359000	-7.988677000
1	-0.782238000	-3.405608000	-6.230820000
1	-2.391063000	-1.679617000	-7.018932000
1	-1.161830000	-0.910181000	-5.997499000
1	1.603402000	-0.864308000	-5.836877000
1	0.767341000	-2.513189000	-4.264694000

^{6}**TS2**$_{d}$:

26	0.075252000	-0.397226000	-1.329243000
15	0.322495000	2.224297000	-1.101123000
15	2.057662000	-1.645918000	-0.079445000
15	-2.280251000	-1.511410000	-0.747855000
7	0.171609000	-0.647067000	-3.157339000
6	-1.908786000	-2.041324000	1.028449000
6	1.763307000	-3.389057000	0.734542000
1	0.703528000	-3.338057000	1.003019000
6	-0.962692000	-1.271639000	1.778635000
6	2.334495000	-0.410567000	1.318271000
6	-0.155535000	3.216811000	-2.712766000
1	0.644542000	2.879129000	-3.386278000
6	-1.243818000	1.381050000	1.130648000
6	1.479014000	1.398218000	2.683433000
1	0.651618000	2.015879000	3.020093000
6	-2.746949000	-3.151263000	-1.667807000
1	-3.565430000	-3.627573000	-1.115347000
5	-0.309829000	0.111889000	1.326645000
6	-0.624296000	-1.783673000	3.063114000
1	0.100045000	-1.236314000	3.659169000
6	-1.196660000	-2.941978000	3.593974000
1	-0.913789000	-3.281919000	4.586397000
6	3.613098000	-0.178109000	1.857474000
1	4.453415000	-0.785189000	1.546913000
6	-2.139473000	-3.659686000	2.846463000
1	-2.596141000	-4.559591000	3.246715000
6	3.829526000	0.833297000	2.800127000
1	4.823229000	0.993356000	3.206895000
6	2.752141000	1.630958000	3.208037000
1	2.903474000	2.422025000	3.937204000
6	3.673315000	-1.819744000	-1.154461000
1	3.309664000	-2.499112000	-1.937587000
6	-2.376664000	1.525500000	1.982824000
1	-2.604429000	0.718114000	2.672888000
6	-0.995472000	2.472659000	0.230075000
6	1.979488000	3.030290000	-0.472841000
1	2.373805000	2.252977000	0.188467000
6	-3.956607000	-0.535204000	-0.555503000
1	-3.738707000	0.110337000	0.301899000
6	-1.797257000	3.627751000	0.260332000
1	-1.584621000	4.453908000	-0.405968000
6	-3.178573000	2.668424000	1.994665000
1	-4.021208000	2.733529000	2.677487000
6	-3.225251000	-2.847289000	-3.099493000
1	-3.393570000	-3.789321000	-3.637172000
1	-2.474210000	-2.273261000	-3.654979000
1	-4.164723000	-2.288018000	-3.116714000
6	-2.882376000	3.737236000	1.137599000
1	-3.485909000	4.639528000	1.146749000
6	-1.531993000	-4.092492000	-1.686368000
1	-1.785873000	-5.020443000	-2.214898000
1	-1.202903000	-4.357957000	-0.677759000
1	-0.689687000	-3.624030000	-2.209648000
6	-0.132433000	4.756679000	-2.696594000
1	-0.229039000	5.121149000	-3.728528000
1	0.794629000	5.164940000	-2.289626000
1	-0.967977000	5.178021000	-2.130226000
6	3.982636000	-0.462625000	-1.809779000
1	4.797446000	-0.574799000	-2.536855000
1	4.299638000	0.275734000	-1.064980000
1	3.107635000	-0.066059000	-2.335340000
6	-2.483012000	-3.206872000	1.567175000
1	-3.195198000	-3.784577000	0.986750000
6	2.968167000	3.224886000	-1.636648000
1	3.951100000	3.501363000	-1.234347000
1	2.655748000	4.024922000	-2.315400000
1	3.095211000	2.310108000	-2.224284000
6	-1.491956000	2.694099000	-3.267102000
1	-1.671755000	3.116710000	-4.264085000
1	-2.328303000	2.990988000	-2.624930000
1	-1.492550000	1.602346000	-3.349769000
6	1.953437000	-4.513549000	-0.298951000
1	1.609421000	-5.463330000	0.129769000
1	3.004170000	-4.641923000	-0.578341000
1	1.376249000	-4.333774000	-1.211668000
6	1.791150000	4.308610000	0.359031000
1	2.759356000	4.612127000	0.779340000
1	1.100429000	4.145652000	1.190962000
1	1.413435000	5.144743000	-0.238130000
6	2.570517000	-3.633527000	2.018650000
1	2.264713000	-4.590956000	2.461052000
1	2.392196000	-2.848722000	2.758684000
1	3.647387000	-3.685161000	1.827942000
6	-4.246949000	0.366691000	-1.765881000
1	-5.121678000	0.994654000	-1.551859000
1	-4.469487000	-0.209467000	-2.669925000
1	-3.403181000	1.026571000	-1.981531000
6	4.942248000	-2.444566000	-0.545507000
1	5.659809000	-2.642344000	-1.353627000
1	4.752717000	-3.393596000	-0.038580000
1	5.436849000	-1.772360000	0.161336000
6	-5.149346000	-1.437234000	-0.202008000
1	-6.026902000	-0.812281000	0.009740000
1	-4.953471000	-2.044747000	0.687207000
1	-5.417176000	-2.107536000	-1.026792000
6	1.207967000	0.367323000	1.740627000

7	0.272380000	-0.779611000	-4.393014000
1	4.064651000	2.368504000	-6.289800000
8	1.820726000	-0.176840000	-6.757353000
6	1.197169000	-0.111109000	-8.094257000
6	-0.272307000	0.285069000	-8.021540000
1	1.770898000	0.584005000	-8.717516000
1	1.314213000	-1.115294000	-8.510324000
1	-0.700349000	0.312099000	-9.030208000
1	-0.842313000	-0.438710000	-7.429674000
1	-0.401622000	1.278196000	-7.577955000
6	2.316505000	1.105236000	-6.221423000
6	3.717623000	1.425285000	-6.727492000
1	1.605392000	1.898862000	-6.479233000
1	2.303880000	0.973253000	-5.137233000
1	4.422471000	0.636740000	-6.444711000
1	3.739254000	1.535129000	-7.817120000
1	1.615971000	-4.461641000	-3.759295000
8	1.976901000	-2.590478000	-5.710712000
6	3.276527000	-2.836549000	-6.439798000
6	4.425612000	-2.837087000	-5.453792000
1	3.146786000	-3.791859000	-6.949807000
1	3.339444000	-2.027632000	-7.165355000
1	4.497905000	-1.877559000	-4.933394000
1	4.321184000	-3.634457000	-4.713124000
1	5.360693000	-2.997611000	-6.002399000
6	0.915229000	-3.658700000	-5.640447000
6	1.377754000	-4.806516000	-4.768909000
1	0.067014000	-3.122503000	-5.218260000
1	0.705957000	-3.956243000	-6.670933000
1	0.564786000	-5.537727000	-4.699725000
1	2.248998000	-5.320353000	-5.185660000
1	1.631262000	-1.622056000	-5.700727000
1	-0.322941000	-0.113044000	-4.946423000

^{2}**TS3**$_{d}$:

26	-2.046087000	-1.113956000	-11.261006000
15	-2.743086000	1.262030000	-10.777837000
15	-1.366953000	-2.141479000	-9.103290000
15	-4.283316000	-2.318031000	-12.117281000
7	-0.764984000	-1.437843000	-12.439282000
6	-5.049912000	-3.128852000	-10.577041000
6	-1.787992000	-4.029073000	-8.847150000
1	-2.755752000	-4.111594000	-9.349597000
6	-4.931190000	-2.463216000	-9.325811000
6	-2.185336000	-1.204509000	-7.674605000
6	-2.310207000	2.310306000	-12.363114000
1	-1.225832000	2.142946000	-12.404849000
6	-5.206377000	0.202657000	-9.810445000
6	-4.038430000	0.147412000	-6.871194000
1	-5.017072000	0.591529000	-7.024235000
6	-3.857681000	-3.809308000	-13.288496000
1	-4.805353000	-4.293251000	-13.554764000
5	-4.434953000	-0.980890000	-9.108226000
6	-5.417785000	-3.128037000	-8.173051000
1	-5.317774000	-2.648772000	-7.204229000
6	-6.042084000	-4.377748000	-8.250681000
1	-6.416141000	-4.854570000	-7.350767000
6	-1.516222000	-0.956140000	-6.467000000
1	-0.531528000	-1.364440000	-6.291293000
6	-6.168567000	-5.009284000	-9.490168000
1	-6.647147000	-5.980058000	-9.566429000
6	-2.104803000	-0.176635000	-5.460939000
1	-1.569555000	-0.004226000	-4.533149000
6	-3.368353000	0.380126000	-5.664492000
1	-3.827242000	0.997095000	-4.899147000
6	0.570745000	-2.023634000	-8.925073000
1	0.876410000	-2.587364000	-9.816988000
6	-6.614957000	0.195148000	-9.667326000
1	-7.093696000	-0.655510000	-9.192287000
6	-4.610502000	1.316051000	-10.464802000
6	-1.920532000	2.223566000	-9.287810000
1	-1.800114000	1.431297000	-8.542593000
6	-5.855606000	-1.468865000	-12.929753000
1	-6.031742000	-0.639321000	-12.238712000
6	-5.414297000	2.369348000	-10.925268000
1	-4.969856000	3.218819000	-11.424293000
6	-7.407347000	1.258737000	-10.114406000
1	-8.483512000	1.227654000	-9.980222000
6	-3.170628000	-3.302087000	-14.571799000
1	-2.892010000	-4.162946000	-15.192981000
1	-2.258068000	-2.746195000	-14.328139000
1	-3.812397000	-2.656899000	-15.172917000
6	-6.804689000	2.348728000	-10.745426000
1	-7.405179000	3.178327000	-11.103596000
6	-2.941220000	-4.818648000	-12.579070000
1	-2.721847000	-5.652854000	-13.257445000
1	-3.382466000	-5.233637000	-11.670146000
1	-1.993081000	-4.341270000	-12.310761000
6	-2.556144000	3.831627000	-12.346194000
1	-1.970840000	4.285783000	-13.156267000
1	-2.254755000	4.312919000	-11.414826000
1	-3.604062000	4.079873000	-12.536604000
6	1.040279000	-0.567423000	-9.079200000
1	2.136836000	-0.533169000	-9.075441000
1	0.686412000	0.055737000	-8.251395000
1	0.693209000	-0.121216000	-10.014906000
6	-5.665339000	-4.389002000	-10.642346000
1	-5.761132000	-4.903938000	-11.591648000
6	-0.522980000	2.748309000	-9.666825000
1	-0.001159000	3.063966000	-8.755106000
1	-0.574782000	3.615910000	-10.330755000
1	0.092731000	1.985958000	-10.152529000
6	-2.940331000	1.675941000	-13.613271000
1	-2.574429000	2.192573000	-14.509542000
1	-4.031197000	1.770297000	-13.597721000
1	-2.683549000	0.616757000	-13.704809000
6	-0.776240000	-4.921030000	-9.589258000
1	-1.175611000	-5.940565000	-9.649964000
1	0.183641000	-4.976093000	-9.067578000
1	-0.590065000	-4.577417000	-10.611415000
6	-2.804176000	3.322967000	-8.677008000
1	-2.293386000	3.744136000	-7.801412000
1	-3.768795000	2.930986000	-8.344371000
1	-2.992692000	4.144279000	-9.375788000

6	-1.969151000	-4.445844000	-7.379324000
1	-2.282506000	-5.497127000	-7.344869000
1	-2.739667000	-3.851761000	-6.881447000
1	-1.043909000	-4.357372000	-6.801618000
6	-5.585297000	-0.890329000	-14.330288000
1	-6.373511000	-0.166684000	-14.573397000
1	-5.617575000	-1.670934000	-15.096994000
1	-4.625585000	-0.373985000	-14.402263000
6	1.254583000	-2.691252000	-7.714872000
1	2.322912000	-2.800991000	-7.941434000
1	0.863588000	-3.683811000	-7.488695000
1	1.184707000	-2.080843000	-6.810839000
6	-7.101902000	-2.367189000	-12.950805000
1	-7.945039000	-1.797650000	-13.363043000
1	-7.387328000	-2.710038000	-11.953726000
1	-6.958842000	-3.245318000	-13.592005000
6	-3.482433000	-0.661866000	-7.890604000
7	0.568599000	-1.503945000	-12.655894000
1	0.990729000	-0.793523000	-13.270011000
1	1.158767000	-1.856397000	-11.903111000
1	4.987324000	-3.880265000	-15.192882000
8	1.616497000	-3.279073000	-14.817561000
6	0.880698000	-4.495073000	-14.275905000
6	0.406445000	-5.360777000	-15.420733000
1	1.573103000	-5.000223000	-13.601195000
1	0.064723000	-4.047397000	-13.711059000
1	-0.186371000	-6.181095000	-15.000966000
1	-0.229332000	-4.795893000	-16.107200000
1	1.233945000	-5.808001000	-15.977991000
6	2.948514000	-3.392876000	-15.544272000
6	4.052582000	-3.861662000	-14.621111000
1	2.745457000	-4.074784000	-16.368001000
1	3.092947000	-2.381947000	-15.919449000
1	4.188165000	-3.181533000	-13.775267000
1	3.878718000	-4.873606000	-14.244830000
1	4.221806000	0.969190000	-16.036391000
8	2.069401000	-0.351347000	-14.736113000
6	1.389846000	0.388139000	-15.825131000
6	0.075767000	-0.305170000	-16.143221000
1	1.226927000	1.418926000	-15.485981000
1	2.047219000	0.408689000	-16.700087000
1	0.240245000	-1.334667000	-16.476834000
1	-0.588270000	-0.319358000	-15.272693000
1	-0.434079000	0.232005000	-16.950228000
6	3.254705000	0.344486000	-14.178200000
6	4.440039000	0.389269000	-15.134443000
1	2.945088000	1.355230000	-13.883381000
1	3.499588000	-0.222250000	-13.276438000
1	5.289009000	0.867015000	-14.632424000
1	4.750271000	-0.617392000	-15.432195000
1	1.450908000	-2.423441000	-14.332079000

^{4}**TS3**$_d$:

26	0.891582000	1.001884000	2.056318000
15	0.054479000	3.480519000	2.440765000
15	2.700616000	0.665433000	3.906747000
15	-1.112183000	-0.626901000	2.744872000
7	1.898302000	0.778668000	0.558388000
6	-0.915977000	-1.042985000	4.583971000
6	2.899365000	-1.080180000	4.761175000
1	1.860562000	-1.415332000	4.843841000
6	-0.459421000	-0.023477000	5.464688000
6	2.382176000	1.892092000	5.320668000
6	-0.434124000	4.242551000	0.712549000
1	0.546327000	4.205761000	0.218988000
6	-1.483087000	2.359747000	4.589732000
6	0.808983000	3.232233000	6.604175000
1	-0.210855000	3.523157000	6.836526000
6	-0.854010000	-2.313772000	1.825856000
1	-1.583778000	-3.018780000	2.239338000
5	-0.259237000	1.501340000	5.098983000
6	-0.243809000	-0.374204000	6.820153000
1	0.127662000	0.378452000	7.508416000
6	-0.508410000	-1.661441000	7.301324000
1	-0.346335000	-1.892631000	8.348854000
6	3.432081000	2.526569000	6.002013000
1	4.458841000	2.275905000	5.777468000
6	-0.963328000	-2.647362000	6.422954000
1	-1.160965000	-3.652998000	6.778989000
6	3.179070000	3.491033000	6.987850000
1	4.008891000	3.960218000	7.505864000
6	1.863366000	3.846911000	7.289232000
1	1.655836000	4.601358000	8.040753000
6	4.435465000	1.044702000	3.102714000
1	4.430513000	0.303518000	2.292505000
6	-2.672695000	2.210398000	5.345833000
1	-2.711791000	1.452654000	6.122141000
6	-1.472410000	3.348794000	3.561705000
6	1.212959000	4.823547000	3.262894000
1	1.772207000	4.227034000	3.988432000
6	-3.040400000	-0.302735000	2.644068000
1	-3.194244000	0.360569000	3.501119000
6	-2.611345000	4.137895000	3.342968000
1	-2.614627000	4.884653000	2.561824000
6	-3.795140000	3.018622000	5.132683000
1	-4.683964000	2.884378000	5.740074000
6	-1.129835000	-2.141573000	0.321528000
1	-0.919832000	-3.085244000	-0.196765000
1	-0.484330000	-1.367949000	-0.110358000
1	-2.169365000	-1.875762000	0.114894000
6	-3.764301000	3.984554000	4.125908000
1	-4.627439000	4.615263000	3.940671000
6	0.562607000	-2.855098000	2.064241000
1	0.679328000	-3.818849000	1.553232000
1	0.776763000	-3.009764000	3.124882000
1	1.315114000	-2.168057000	1.661165000
6	-0.937143000	5.697880000	0.642855000
1	-0.886238000	6.030850000	-0.402107000
1	-0.340700000	6.391990000	1.236069000
1	-1.981210000	5.792854000	0.954251000
6	4.423999000	2.443130000	2.465554000
1	5.356825000	2.598997000	1.908888000
1	4.355583000	3.230060000	3.224462000
1	3.588154000	2.549135000	1.769055000

6	-1.153505000	-2.338076000	5.068811000
1	-1.481988000	-3.124066000	4.398666000
6	2.213829000	5.394996000	2.241375000
1	2.981796000	5.964382000	2.779420000
1	1.739539000	6.079052000	1.532120000
1	2.722652000	4.611251000	1.673208000
6	-1.385747000	3.309376000	-0.054312000
1	-1.475465000	3.655494000	-1.091594000
1	-2.389894000	3.320111000	0.381911000
1	-1.027756000	2.274884000	-0.072480000
6	3.651100000	-2.058979000	3.839374000
1	3.533029000	-3.078554000	4.226511000
1	4.723433000	-1.847021000	3.800722000
1	3.265564000	-2.046914000	2.815717000
6	0.465967000	5.928959000	4.025481000
1	1.200370000	6.575932000	4.522616000
1	-0.191320000	5.518795000	4.796529000
1	-0.136168000	6.559855000	3.364425000
6	3.496720000	-1.040059000	6.176965000
1	3.492383000	-2.055399000	6.594313000
1	2.911761000	-0.402106000	6.844628000
1	4.531254000	-0.683404000	6.185613000
6	-3.446999000	0.457527000	1.373549000
1	-4.505872000	0.736564000	1.445101000
1	-3.328964000	-0.146915000	0.468536000
1	-2.866443000	1.373359000	1.253452000
6	5.716646000	0.826882000	3.930853000
1	6.571778000	0.812293000	3.242365000
1	5.724678000	-0.114516000	4.481883000
1	5.896802000	1.639803000	4.639664000
6	-3.878508000	-1.573242000	2.850687000
1	-4.937727000	-1.294158000	2.918279000
1	-3.617596000	-2.096647000	3.775350000
1	-3.779044000	-2.272976000	2.013572000
6	1.035044000	2.231846000	5.628018000
7	2.579598000	-0.045309000	-0.224047000
1	3.455087000	0.261148000	-0.665891000
1	2.454098000	-1.056375000	-0.140249000
1	-1.080458000	2.148313000	-3.422573000
8	1.624912000	0.086836000	-3.013725000
6	2.091120000	-0.680883000	-4.236063000
6	1.725555000	-2.145436000	-4.129359000
1	1.613573000	-0.170505000	-5.071888000
1	3.163188000	-0.493548000	-4.246287000
1	2.085026000	-2.654636000	-5.030672000
1	2.198074000	-2.616405000	-3.262802000
1	0.643817000	-2.296324000	-4.072623000
6	0.193757000	0.551806000	-2.826880000
6	-0.040299000	1.841986000	-3.580749000
1	-0.424191000	-0.276883000	-3.173510000
1	0.101386000	0.657745000	-1.745978000
1	0.611198000	2.640694000	-3.216252000
1	0.108105000	1.720751000	-4.657477000
1	6.337361000	2.503237000	-1.555167000
8	4.450215000	0.555327000	-2.282462000
6	5.672375000	-0.265293000	-2.432006000
6	5.432904000	-1.628603000	-1.806480000
1	5.896227000	-0.348430000	-3.503010000
1	6.507482000	0.246996000	-1.942596000
1	5.244281000	-1.544479000	-0.730922000
1	4.585388000	-2.140050000	-2.274513000
1	6.320500000	-2.255573000	-1.942603000
6	4.603963000	1.948985000	-2.762184000
6	5.314513000	2.840307000	-1.751597000
1	5.133647000	1.924158000	-3.722418000
1	3.582940000	2.294197000	-2.941462000
1	5.371471000	3.863238000	-2.140898000
1	4.768052000	2.866435000	-0.803240000
1	2.236424000	0.145128000	-2.229189000

^{6}**TS3**$_{p}$:

26	-0.007609000	-0.516747000	-1.785618000
15	-0.607337000	2.006407000	-1.085299000
15	2.228607000	-1.022723000	-0.392371000
15	-1.842331000	-2.101179000	-0.629807000
7	0.223258000	-0.631298000	-3.735208000
6	-1.193943000	-2.573146000	1.090788000
6	2.548662000	-2.794643000	0.361819000
1	1.549055000	-3.076297000	0.706066000
6	-0.467203000	-1.610954000	1.846756000
6	2.338464000	0.183856000	1.065975000
6	-1.501680000	2.901597000	-2.576086000
1	-0.695022000	2.867491000	-3.320851000
6	-1.567943000	0.836824000	1.358752000
6	1.205641000	1.560703000	2.720571000
1	0.293084000	1.890580000	3.207106000
6	-1.928113000	-3.779959000	-1.590667000
1	-2.539588000	-4.463375000	-0.991449000
5	-0.291136000	-0.079446000	1.501934000
6	0.074796000	-2.023872000	3.089288000
1	0.652718000	-1.315262000	3.673892000
6	-0.125845000	-3.313637000	3.592985000
1	0.290138000	-3.591027000	4.555712000
6	3.556192000	0.752354000	1.468727000
1	4.480228000	0.459637000	0.991107000
6	-0.848280000	-4.242105000	2.840870000
1	-1.004009000	-5.249639000	3.211786000
6	3.606212000	1.703731000	2.497650000
1	4.561460000	2.122898000	2.795590000
6	2.427553000	2.110044000	3.125465000
1	2.453358000	2.853165000	3.915430000
6	3.707665000	-0.703874000	-1.623434000
1	3.468279000	-1.444311000	-2.397873000
6	-2.522916000	0.692711000	2.397010000
1	-2.380460000	-0.086155000	3.139527000
6	-1.797510000	1.853372000	0.386540000
6	0.780593000	3.258789000	-0.514458000
1	1.460113000	2.606306000	0.040266000
6	-3.699952000	-1.636485000	-0.240384000
1	-3.579893000	-0.928519000	0.585177000
6	-2.934621000	2.668406000	0.486360000
1	-3.121607000	3.432914000	-0.253286000
6	-3.638546000	1.530561000	2.502195000
1	-4.340536000	1.400049000	3.318895000

WILEY-VCH

6	-2.606154000	-3.581746000	-2.958082000
1	-2.560552000	-4.521513000	-3.522685000
1	-2.098875000	-2.805816000	-3.543409000
1	-3.659836000	-3.308594000	-2.866282000
6	-3.847176000	2.519499000	1.539663000
1	-4.712361000	3.171450000	1.597438000
6	-0.518946000	-4.368048000	-1.755657000
1	-0.581143000	-5.333709000	-2.272842000
1	-0.024719000	-4.536667000	-0.795387000
1	0.111000000	-3.702108000	-2.356612000
6	-1.916107000	4.379641000	-2.435646000
1	-2.178722000	4.756335000	-3.432779000
1	-1.119933000	5.015317000	-2.046463000
1	-2.798126000	4.513161000	-1.804074000
6	3.588587000	0.695955000	-2.249218000
1	4.375753000	0.825893000	-3.002691000
1	3.715640000	1.482987000	-1.498523000
1	2.625149000	0.836478000	-2.746480000
6	-1.366300000	-3.873176000	1.591603000
1	-1.900975000	-4.618768000	1.014798000
6	1.544358000	3.825788000	-1.725146000
1	2.418760000	4.380973000	-1.363939000
1	0.937937000	4.520097000	-2.314072000
1	1.909111000	3.038411000	-2.391153000
6	-2.669759000	2.054071000	-3.106352000
1	-3.034174000	2.485797000	-4.046845000
1	-3.507578000	2.045719000	-2.402076000
1	-2.371775000	1.020598000	-3.305630000
6	2.979107000	-3.787461000	-0.732337000
1	2.951185000	-4.805126000	-0.324067000
1	4.000101000	-3.602305000	-1.078456000
1	2.310947000	-3.760645000	-1.598212000
6	0.293663000	4.361480000	0.439311000
1	1.159866000	4.943825000	0.779187000
1	-0.196687000	3.946781000	1.324019000
1	-0.402285000	5.055023000	-0.041402000
6	3.494718000	-2.814581000	1.572478000
1	3.533400000	-3.834339000	1.976632000
1	3.147310000	-2.152609000	2.369927000
1	4.516563000	-2.523859000	1.309991000
6	-4.383158000	-0.906109000	-1.406657000
1	-5.335580000	-0.487618000	-1.057911000
1	-4.607732000	-1.576385000	-2.241822000
1	-3.772752000	-0.083673000	-1.783007000
6	5.150428000	-0.970500000	-1.151999000
1	5.798387000	-1.014784000	-2.037084000
1	5.267173000	-1.913227000	-0.614979000
1	5.534974000	-0.165542000	-0.519769000
6	-4.526623000	-2.833600000	0.253081000
1	-5.516335000	-2.478156000	0.566771000
1	-4.067341000	-3.329649000	1.113309000
1	-4.682734000	-3.577363000	-0.536182000
6	1.128504000	0.577669000	1.704554000
7	0.438036000	0.396540000	-4.599428000
1	0.306233000	0.199947000	-5.588429000
1	0.455393000	1.363062000	-4.294032000
1	4.515011000	-3.452593000	-5.037238000
8	1.127996000	-3.026082000	-5.296407000
6	0.489777000	-4.369505000	-5.595211000
6	0.542928000	-4.735334000	-7.062936000
1	1.027396000	-5.068269000	-4.956387000
1	-0.525190000	-4.242878000	-5.220523000
1	0.070387000	-5.716861000	-7.181927000
1	-0.006725000	-4.020929000	-7.679115000
1	1.568502000	-4.820382000	-7.433686000
6	2.591129000	-2.732000000	-5.569428000
6	3.473487000	-3.767257000	-4.909473000
1	2.711648000	-2.693851000	-6.653369000
1	2.709834000	-1.737527000	-5.139983000
1	3.269576000	-3.841094000	-3.838515000
1	3.373983000	-4.756187000	-5.365012000
1	-2.196705000	0.421566000	-8.154184000
8	-0.318733000	-1.217954000	-6.809363000
6	0.264694000	-1.109860000	-8.170282000
6	0.951016000	0.233306000	-8.377583000
1	0.982660000	-1.930020000	-8.259143000
1	-0.530150000	-1.275680000	-8.905180000
1	0.239320000	1.062089000	-8.316495000
1	1.736067000	0.390903000	-7.630124000
1	1.420406000	0.259117000	-9.367735000
6	-1.799534000	-1.195717000	-6.741984000
6	-2.401172000	0.153464000	-7.112667000
1	-2.182907000	-1.994711000	-7.388162000
1	-2.015821000	-1.448765000	-5.702159000
1	-3.489585000	0.110897000	-6.990057000
1	-2.015305000	0.946182000	-6.463913000
1	0.530074000	-2.214560000	-5.353539000

[2]$\mathbf{4}_{ddd}$:

26	-0.091814000	-0.203678000	-1.354664000
15	-0.489868000	2.147619000	-0.951359000
15	2.124588000	-0.904872000	-0.648173000
15	-1.869550000	-1.570871000	-0.407907000
7	-0.094453000	-0.404059000	-2.890398000
6	-1.165642000	-2.178280000	1.245472000
6	2.364541000	-2.734644000	-0.005833000
1	1.381997000	-2.957618000	0.420208000
6	-0.252697000	-1.341501000	1.945448000
6	2.569546000	0.235954000	0.798558000
6	-1.557568000	2.903981000	-2.390601000
1	-0.854829000	2.781763000	-3.224128000
6	-1.166898000	1.200584000	1.656783000
6	1.826753000	1.671965000	2.617230000
1	1.032592000	2.066267000	3.243454000
6	-2.234636000	-3.166539000	-1.455146000
1	-3.065571000	-3.671537000	-0.946062000
5	0.030633000	0.176104000	1.633783000
6	0.362733000	-1.862946000	3.110330000
1	1.083903000	-1.253365000	3.645725000
6	0.045817000	-3.133722000	3.602236000
1	0.520999000	-3.499254000	4.506303000
6	3.877299000	0.699616000	1.000763000
1	4.685529000	0.349194000	0.375010000
6	-0.876423000	-3.929773000	2.918061000

1	-1.131839000	-4.916789000	3.288678000
6	4.166018000	1.622932000	2.016008000
1	5.187233000	1.959142000	2.160533000
6	3.138666000	2.112323000	2.824931000
1	3.350475000	2.837996000	3.602912000
6	3.404617000	-0.693926000	-2.099897000
1	2.981288000	-1.399284000	-2.826572000
6	-1.977644000	1.167015000	2.817806000
1	-1.813992000	0.387016000	3.554666000
6	-1.431856000	2.213318000	0.690624000
6	1.048448000	3.334254000	-0.762828000
1	1.804422000	2.658725000	-0.352600000
6	-3.623968000	-0.852261000	0.101498000
1	-3.370723000	0.157689000	0.436450000
6	-2.442801000	3.156463000	0.923834000
1	-2.649622000	3.928254000	0.196284000
6	-2.971806000	2.124099000	3.050199000
1	-3.565079000	2.080533000	3.957299000
6	-2.656445000	-2.801226000	-2.892405000
1	-2.817181000	-3.729082000	-3.455074000
1	-1.863909000	-2.235149000	-3.393233000
1	-3.577945000	-2.221445000	-2.942159000
6	-3.201705000	3.123331000	2.102454000
1	-3.970857000	3.870528000	2.266393000
6	-1.010213000	-4.093916000	-1.515375000
1	-1.276260000	-5.000222000	-2.072884000
1	-0.642954000	-4.400359000	-0.534028000
1	-0.195718000	-3.599366000	-2.051582000
6	-1.944110000	4.393466000	-2.296539000
1	-2.199458000	4.747088000	-3.303333000
1	-1.145256000	5.032549000	-1.917166000
1	-2.830423000	4.544656000	-1.673656000
6	3.323521000	0.715193000	-2.705397000
1	3.980563000	0.768785000	-3.582514000
1	3.656484000	1.479758000	-1.994904000
1	2.307543000	0.951706000	-3.027757000
6	-1.467123000	-3.456329000	1.738195000
1	-2.166050000	-4.096405000	1.212156000
6	1.540687000	3.842764000	-2.130573000
1	2.534290000	4.289339000	-2.003363000
1	0.885862000	4.616038000	-2.541838000
1	1.631641000	3.042873000	-2.870109000
6	-2.792631000	2.036673000	-2.673847000
1	-3.305708000	2.419612000	-3.564693000
1	-3.503296000	2.070224000	-1.840845000
1	-2.515427000	0.996648000	-2.860509000
6	2.627162000	-3.708632000	-1.169099000
1	2.480012000	-4.735106000	-0.812262000
1	3.652534000	-3.634649000	-1.541869000
1	1.950083000	-3.551935000	-2.013357000
6	0.832154000	4.484254000	0.234418000
1	1.768609000	5.048140000	0.331151000
1	0.558434000	4.118577000	1.227396000
1	0.058157000	5.184002000	-0.097426000
6	3.414451000	-2.869389000	1.108685000
1	3.425975000	-3.909140000	1.459457000
1	3.184383000	-2.229841000	1.964553000
1	4.423830000	-2.625993000	0.762371000
6	-4.611089000	-0.752890000	-1.073032000
1	-5.474443000	-0.157435000	-0.750936000
1	-4.987807000	-1.738459000	-1.364427000
1	-4.184516000	-0.266923000	-1.952370000
6	4.870488000	-1.089592000	-1.833444000
1	5.360740000	-1.257376000	-2.800783000
1	4.981810000	-2.003455000	-1.248409000
1	5.425672000	-0.291374000	-1.332636000
6	-4.260092000	-1.621556000	1.270697000
1	-5.221010000	-1.152752000	1.517353000
1	-3.639053000	-1.606339000	2.168866000
1	-4.463507000	-2.666377000	1.007489000
6	1.511737000	0.710710000	1.626343000
7	1.446022000	-2.432981000	-4.968719000
1	0.907717000	-1.829448000	-4.357274000
1	2.268465000	-1.985479000	-5.352591000
1	1.640114000	-3.342776000	-4.571103000

^{4}**4**$_{ddd}$:

26	-0.060241000	-0.346192000	-1.635208000
15	-0.468996000	2.133539000	-1.016297000
15	2.180283000	-1.012558000	-0.675655000
15	-1.829831000	-1.692347000	-0.443720000
7	0.079296000	-0.023567000	-3.419777000
6	-1.147027000	-2.248221000	1.236595000
6	2.459287000	-2.796461000	0.076162000
1	1.478071000	-3.010152000	0.511752000
6	-0.265880000	-1.365182000	1.913835000
6	2.576845000	0.187168000	0.741896000
6	-1.541359000	2.938337000	-2.427910000
1	-0.875004000	2.761206000	-3.281687000
6	-1.177495000	1.179258000	1.570246000
6	1.783422000	1.647318000	2.516338000
1	0.970336000	2.048154000	3.113573000
6	-2.201142000	-3.352715000	-1.387354000
1	-2.975565000	-3.880999000	-0.816793000
5	0.011246000	0.138334000	1.507259000
6	0.324439000	-1.816127000	3.119073000
1	1.019642000	-1.167484000	3.643479000
6	0.025798000	-3.072350000	3.659676000
1	0.488689000	-3.386635000	4.589532000
6	3.877363000	0.658517000	0.977249000
1	4.704870000	0.294348000	0.386049000
6	-0.861678000	-3.919792000	2.991360000
1	-1.101224000	-4.896775000	3.398343000
6	4.137079000	1.603713000	1.979755000
1	5.153002000	1.947733000	2.144313000
6	3.086115000	2.101217000	2.752098000
1	3.272315000	2.841784000	3.523100000
6	3.556076000	-0.825771000	-2.049757000
1	3.178946000	-1.549188000	-2.785375000
6	-1.975524000	1.146583000	2.739125000
1	-1.812456000	0.358993000	3.468453000
6	-1.432235000	2.206462000	0.617842000
6	1.042602000	3.347875000	-0.771279000
1	1.776595000	2.695276000	-0.287905000

6	-3.587340000	-0.974381000	0.054770000
1	-3.317144000	0.015657000	0.434948000
6	-2.429693000	3.159577000	0.871692000
1	-2.627780000	3.944849000	0.155487000
6	-2.962313000	2.108395000	2.986891000
1	-3.551649000	2.060130000	3.896862000
6	-2.721298000	-3.075193000	-2.812138000
1	-2.810549000	-4.026343000	-3.351949000
1	-2.021494000	-2.437810000	-3.364821000
1	-3.699362000	-2.594337000	-2.827426000
6	-3.188539000	3.119078000	2.050525000
1	-3.952348000	3.870046000	2.224995000
6	-0.935375000	-4.222118000	-1.473429000
1	-1.174667000	-5.167517000	-1.976163000
1	-0.506191000	-4.459419000	-0.497474000
1	-0.169153000	-3.713487000	-2.068830000
6	-1.872199000	4.441922000	-2.377954000
1	-2.236862000	4.750268000	-3.367140000
1	-1.011705000	5.068454000	-2.136890000
1	-2.669347000	4.667819000	-1.663438000
6	3.495874000	0.577315000	-2.677130000
1	4.170627000	0.623415000	-3.541945000
1	3.818750000	1.344380000	-1.964583000
1	2.483493000	0.823848000	-3.010366000
6	-1.438155000	-3.508048000	1.781716000
1	-2.116254000	-4.182728000	1.271057000
6	1.622396000	3.790838000	-2.127866000
1	2.592893000	4.275842000	-1.962428000
1	0.978561000	4.515070000	-2.635663000
1	1.783377000	2.946258000	-2.803554000
6	-2.813902000	2.105039000	-2.648850000
1	-3.347778000	2.472597000	-3.534847000
1	-3.496177000	2.178141000	-1.794466000
1	-2.568474000	1.052416000	-2.810626000
6	2.732216000	-3.832054000	-1.030399000
1	2.656802000	-4.840543000	-0.605095000
1	3.735725000	-3.729538000	-1.453511000
1	2.010586000	-3.767107000	-1.850268000
6	0.780190000	4.539534000	0.163472000
1	1.713988000	5.100984000	0.300995000
1	0.438377000	4.213197000	1.149668000
1	0.035431000	5.232401000	-0.241433000
6	3.502439000	-2.865813000	1.202903000
1	3.529690000	-3.887903000	1.603054000
1	3.252877000	-2.191202000	2.026024000
1	4.510919000	-2.619314000	0.856114000
6	-4.541286000	-0.795657000	-1.138865000
1	-5.359283000	-0.127576000	-0.840989000
1	-4.993581000	-1.748126000	-1.433455000
1	-4.055576000	-0.355805000	-2.012596000
6	5.014267000	-1.217018000	-1.733909000
1	5.546793000	-1.358862000	-2.683618000
1	5.106856000	-2.144325000	-1.166453000
1	5.544905000	-0.429520000	-1.191908000
6	-4.276840000	-1.765458000	1.177477000
1	-5.236816000	-1.287858000	1.413313000
1	-3.682211000	-1.792678000	2.093507000
1	-4.490708000	-2.797698000	0.874233000
6	1.496632000	0.673655000	1.529002000
7	0.629694000	-1.071717000	-4.448749000
1	1.518997000	-0.733820000	-4.825406000
1	0.759405000	-2.007284000	-4.054094000
1	-0.043603000	-1.124773000	-5.216995000

[6] **4**$_{ddd}$:

26	-0.182017000	-0.303595000	-1.757509000
15	-0.398207000	2.224871000	-1.067583000
15	2.172338000	-1.007298000	-0.651547000
15	-1.905813000	-1.762096000	-0.347228000
7	-0.150261000	-0.360505000	-3.595802000
6	-1.127409000	-2.251265000	1.310107000
6	2.469534000	-2.775920000	0.134917000
1	1.488462000	-2.994355000	0.568778000
6	-0.264613000	-1.319653000	1.951267000
6	2.566661000	0.216242000	0.746705000
6	-1.376839000	3.127644000	-2.494336000
1	-0.674535000	2.960890000	-3.321582000
6	-1.198804000	1.214425000	1.491313000
6	1.759799000	1.694490000	2.502663000
1	0.943289000	2.100616000	3.091880000
6	-2.212987000	-3.440503000	-1.263567000
1	-2.837374000	-4.059674000	-0.609381000
5	-0.006354000	0.177337000	1.505882000
6	0.366023000	-1.727248000	3.152317000
1	1.047251000	-1.043861000	3.649679000
6	0.129586000	-2.983019000	3.722954000
1	0.620275000	-3.258028000	4.650856000
6	3.865063000	0.691644000	0.986522000
1	4.697015000	0.319669000	0.406403000
6	-0.724483000	-3.883142000	3.081857000
1	-0.907601000	-4.865212000	3.505414000
6	4.116959000	1.647457000	1.981160000
1	5.131464000	1.993810000	2.149499000
6	3.061023000	2.150906000	2.742694000
1	3.241805000	2.898098000	3.508497000
6	3.526207000	-0.872685000	-2.050840000
1	3.182119000	-1.681790000	-2.709469000
6	-2.044688000	1.179364000	2.627715000
1	-1.911348000	0.388148000	3.359110000
6	-1.414391000	2.252761000	0.537460000
6	1.157189000	3.357951000	-0.738017000
1	1.842329000	2.663486000	-0.244093000
6	-3.682519000	-1.191229000	0.239954000
1	-3.434399000	-0.539282000	1.084472000
6	-2.423971000	3.201265000	0.757063000
1	-2.596469000	3.988234000	0.036505000
6	-3.037402000	2.142091000	2.844900000
1	-3.658933000	2.090512000	3.732784000
6	-2.970930000	-3.189900000	-2.580824000
1	-3.052181000	-4.131121000	-3.138630000
1	-2.442311000	-2.463896000	-3.210300000
1	-3.985113000	-2.818324000	-2.413707000
6	-3.227744000	3.156295000	1.905136000
1	-3.996823000	3.907337000	2.053085000

6	-0.885406000	-4.167466000	-1.526581000
1	-1.079659000	-5.113849000	-2.046435000
1	-0.341928000	-4.394556000	-0.605394000
1	-0.228694000	-3.562988000	-2.163217000
6	-1.636124000	4.643015000	-2.391977000
1	-1.960882000	5.004746000	-3.376597000
1	-0.751613000	5.215534000	-2.109060000
1	-2.437486000	4.882383000	-1.686914000
6	3.375699000	0.456181000	-2.809013000
1	4.048347000	0.463266000	-3.676954000
1	3.648138000	1.306565000	-2.174142000
1	2.348321000	0.593529000	-3.162569000
6	-1.339327000	-3.518342000	1.876050000
1	-1.979083000	-4.240591000	1.381553000
6	1.803190000	3.800712000	-2.064146000
1	2.783176000	4.246407000	-1.851921000
1	1.207453000	4.555919000	-2.584979000
1	1.961406000	2.960607000	-2.746601000
6	-2.676167000	2.375670000	-2.825732000
1	-3.113317000	2.792206000	-3.742413000
1	-3.416973000	2.488881000	-2.026894000
1	-2.494086000	1.310448000	-2.994362000
6	2.758934000	-3.823681000	-0.955959000
1	2.705399000	-4.826858000	-0.514869000
1	3.760116000	-3.706693000	-1.382202000
1	2.033656000	-3.784722000	-1.774731000
6	0.902762000	4.537056000	0.214233000
1	1.853701000	5.048705000	0.412253000
1	0.497840000	4.201966000	1.173084000
1	0.211898000	5.274086000	-0.206557000
6	3.509595000	-2.822092000	1.265494000
1	3.531261000	-3.833961000	1.691498000
1	3.263816000	-2.125787000	2.071457000
1	4.520174000	-2.590831000	0.913640000
6	-4.399943000	-0.346027000	-0.823017000
1	-5.315713000	0.078315000	-0.391796000
1	-4.692631000	-0.937626000	-1.696348000
1	-3.774548000	0.479777000	-1.166162000
6	5.005156000	-1.150801000	-1.716685000
1	5.537877000	-1.371426000	-2.651158000
1	5.149862000	-2.001718000	-1.048344000
1	5.494759000	-0.278165000	-1.274889000
6	-4.560698000	-2.342952000	0.750879000
1	-5.480052000	-1.928042000	1.183947000
1	-4.065305000	-2.928155000	1.531669000
1	-4.859093000	-3.021229000	-0.056484000
6	1.480492000	0.712115000	1.521932000
7	0.309351000	-1.483247000	-4.486251000
1	-0.122680000	-1.379893000	-5.408806000
1	1.326614000	-1.463010000	-4.620910000
1	0.058308000	-2.413925000	-4.132391000

$^{2}\mathbf{4}_{dpd}$:

26	-0.172408000	-0.249214000	-0.840568000
15	-0.561563000	2.198939000	-0.283049000
15	2.152325000	-0.859420000	0.159097000
15	-1.944837000	-1.614163000	0.484203000
7	0.115849000	0.074714000	-2.728029000
6	-1.136795000	-2.168726000	2.099519000
6	2.396875000	-2.657476000	0.877881000
1	1.421847000	-2.858724000	1.330772000
6	-0.270121000	-1.259419000	2.765583000
6	2.512896000	0.346752000	1.573334000
6	-1.519983000	3.052466000	-1.756024000
1	-0.742435000	2.998952000	-2.530905000
6	-1.266899000	1.244503000	2.326534000
6	1.701331000	1.792323000	3.352026000
1	0.885703000	2.178340000	3.955450000
6	-2.270464000	-3.233803000	-0.526073000
1	-2.908219000	-3.865411000	0.102521000
5	-0.042696000	0.248685000	2.363403000
6	0.384379000	-1.712187000	3.936605000
1	1.069293000	-1.046436000	4.452009000
6	0.160512000	-2.992295000	4.456172000
1	0.665285000	-3.304159000	5.364177000
6	3.806233000	0.842105000	1.797461000
1	4.636596000	0.498943000	1.197545000
6	-0.698734000	-3.869313000	3.790195000
1	-0.870248000	-4.868796000	4.175077000
6	4.053464000	1.787026000	2.803195000
1	5.063809000	2.147316000	2.963838000
6	2.998539000	2.263786000	3.583165000
1	3.177058000	3.003424000	4.356173000
6	3.500749000	-0.654589000	-1.237591000
1	3.099798000	-1.339045000	-1.996391000
6	-2.077705000	1.236563000	3.488929000
1	-1.881929000	0.501314000	4.263151000
6	-1.569729000	2.204840000	1.319492000
6	0.968996000	3.380742000	0.051753000
1	1.692681000	2.702391000	0.509568000
6	-3.710498000	-1.042410000	1.106000000
1	-3.448556000	-0.425692000	1.970813000
6	-2.631656000	3.102720000	1.500852000
1	-2.870906000	3.827673000	0.736688000
6	-3.116756000	2.155198000	3.674220000
1	-3.706898000	2.129929000	4.583902000
6	-3.024705000	-2.900485000	-1.827124000
1	-3.136569000	-3.815075000	-2.421602000
1	-2.472669000	-2.172068000	-2.433318000
1	-4.025783000	-2.504291000	-1.642028000
6	-3.397289000	3.088204000	2.674478000
1	-4.206301000	3.800222000	2.796351000
6	-0.963350000	-3.981473000	-0.828392000
1	-1.194828000	-4.914607000	-1.356356000
1	-0.404958000	-4.238398000	0.075240000
1	-0.311066000	-3.388626000	-1.479699000
6	-1.906096000	4.539182000	-1.613385000
1	-2.117835000	4.934828000	-2.614502000
1	-1.119731000	5.155838000	-1.176993000
1	-2.815604000	4.670978000	-1.021505000
6	3.476499000	0.770061000	-1.812413000
1	4.154006000	0.825035000	-2.673193000
1	3.813124000	1.507660000	-1.076750000
1	2.477495000	1.050117000	-2.153005000

6	-1.334299000	-3.460471000	2.608914000
1	-1.975343000	-4.165919000	2.092753000
6	1.573349000	3.931333000	-1.251979000
1	2.516656000	4.435323000	-1.009031000
1	0.926191000	4.670238000	-1.732020000
1	1.803579000	3.148900000	-1.979446000
6	-2.732316000	2.229901000	-2.225534000
1	-3.080814000	2.625181000	-3.187447000
1	-3.562739000	2.313355000	-1.518934000
1	-2.504563000	1.168348000	-2.363123000
6	2.629863000	-3.678450000	-0.250823000
1	2.537161000	-4.689852000	0.162648000
1	3.629125000	-3.594181000	-0.686045000
1	1.897926000	-3.585186000	-1.058237000
6	0.659649000	4.501271000	1.058530000
1	1.588404000	5.047090000	1.265824000
1	0.285003000	4.108883000	2.006582000
1	-0.068399000	5.222531000	0.675031000
6	3.463951000	-2.746087000	1.980602000
1	3.478510000	-3.769832000	2.375112000
1	3.245370000	-2.071057000	2.811923000
1	4.468179000	-2.518783000	1.611187000
6	-4.450818000	-0.162093000	0.091288000
1	-5.368623000	0.219847000	0.555139000
1	-4.742433000	-0.711706000	-0.808635000
1	-3.847148000	0.694737000	-0.207870000
6	4.946438000	-1.095838000	-0.933027000
1	5.485560000	-1.168709000	-1.886078000
1	5.012878000	-2.069183000	-0.446147000
1	5.484734000	-0.368741000	-0.319769000
6	-4.570113000	-2.222938000	1.583039000
1	-5.475538000	-1.827033000	2.059553000
1	-4.055828000	-2.842890000	2.323186000
1	-4.891754000	-2.860579000	0.752507000
6	1.427696000	0.814221000	2.365634000
7	0.479882000	-0.813978000	-3.655154000
1	-0.090987000	0.976264000	-3.160983000
1	0.512350000	-0.573754000	-4.638696000
1	0.703759000	-1.755530000	-3.366149000

$^{4}\mathbf{4}_{\mathrm{dpd}}$:

26	-0.123973000	-0.241789000	-0.862438000
15	-0.568170000	2.301665000	-0.266193000
15	2.212818000	-0.849662000	0.168576000
15	-1.970615000	-1.634231000	0.428390000
7	0.169284000	-0.322996000	-2.843170000
6	-1.147304000	-2.160112000	2.047549000
6	2.451128000	-2.655115000	0.862850000
1	1.477779000	-2.852317000	1.321744000
6	-0.269951000	-1.250241000	2.701652000
6	2.535989000	0.347585000	1.599324000
6	-1.533605000	3.184374000	-1.715002000
1	-0.762037000	3.130795000	-2.494500000
6	-1.268776000	1.263890000	2.313934000
6	1.684041000	1.810663000	3.344365000
1	0.854293000	2.206953000	3.921041000
6	-2.314814000	-3.274827000	-0.539074000
1	-2.949184000	-3.889524000	0.109337000
5	-0.042866000	0.267301000	2.328650000
6	0.394985000	-1.705111000	3.867618000
1	1.087665000	-1.040787000	4.373943000
6	0.170085000	-2.980835000	4.396557000
1	0.683313000	-3.290648000	5.300477000
6	3.826831000	0.828708000	1.864273000
1	4.672191000	0.471678000	1.293523000
6	-0.703312000	-3.854723000	3.745837000
1	-0.879762000	-4.850320000	4.138434000
6	4.052622000	1.775779000	2.873162000
1	5.061556000	2.124185000	3.065890000
6	2.978226000	2.269584000	3.614671000
1	3.139784000	3.011175000	4.389549000
6	3.565462000	-0.609787000	-1.216126000
1	3.170298000	-1.281668000	-1.989380000
6	-2.081844000	1.214693000	3.474495000
1	-1.893665000	0.446386000	4.217838000
6	-1.562888000	2.268970000	1.346821000
6	0.970933000	3.460808000	0.077147000
1	1.685395000	2.769168000	0.531618000
6	-3.723859000	-1.030476000	1.049917000
1	-3.449171000	-0.400917000	1.901577000
6	-2.613850000	3.170428000	1.568737000
1	-2.844758000	3.929451000	0.835243000
6	-3.111626000	2.135375000	3.698797000
1	-3.703050000	2.076458000	4.606136000
6	-3.086123000	-2.968283000	-1.836817000
1	-3.199410000	-3.892789000	-2.415282000
1	-2.553565000	-2.242701000	-2.463144000
1	-4.087716000	-2.577218000	-1.643332000
6	-3.380253000	3.114451000	2.740963000
1	-4.181031000	3.829897000	2.893852000
6	-1.013439000	-4.037085000	-0.829711000
1	-1.246523000	-4.960881000	-1.372472000
1	-0.472899000	-4.309207000	0.080356000
1	-0.335167000	-3.446750000	-1.456600000
6	-1.918515000	4.667052000	-1.544445000
1	-2.158071000	5.075835000	-2.534238000
1	-1.119584000	5.278550000	-1.122601000
1	-2.811106000	4.792261000	-0.925359000
6	3.525792000	0.827468000	-1.760146000
1	4.207640000	0.911097000	-2.615051000
1	3.845440000	1.554146000	-1.006259000
1	2.526029000	1.107025000	-2.103485000
6	-1.347241000	-3.447142000	2.569075000
1	-1.999988000	-4.150990000	2.065904000
6	1.575161000	3.993845000	-1.234433000
1	2.545413000	4.454547000	-1.012645000
1	0.947864000	4.761952000	-1.695606000
1	1.748271000	3.204386000	-1.971969000
6	-2.743875000	2.362569000	-2.187962000
1	-3.108701000	2.776201000	-3.136302000
1	-3.567711000	2.418616000	-1.470058000
1	-2.498571000	1.309067000	-2.355796000
6	2.660122000	-3.657095000	-0.286920000
1	2.576978000	-4.676065000	0.109572000
1	3.649635000	-3.563307000	-0.743015000

1	1.909983000	-3.547734000	-1.075770000
6	0.688986000	4.587238000	1.084417000
1	1.628053000	5.114955000	1.293105000
1	0.305818000	4.200769000	2.032071000
1	-0.025417000	5.322694000	0.701995000
6	3.528194000	-2.772629000	1.952352000
1	3.526252000	-3.798660000	2.341272000
1	3.333519000	-2.097711000	2.789899000
1	4.532660000	-2.564488000	1.572379000
6	-4.452415000	-0.159297000	0.018640000
1	-5.360034000	0.252161000	0.477234000
1	-4.759551000	-0.724264000	-0.866694000
1	-3.832626000	0.677292000	-0.305071000
6	5.012500000	-1.043878000	-0.913911000
1	5.565039000	-1.070005000	-1.861733000
1	5.081383000	-2.037205000	-0.468627000
1	5.533993000	-0.338542000	-0.261677000
6	-4.597429000	-2.189240000	1.553526000
1	-5.495799000	-1.772233000	2.025337000
1	-4.088047000	-2.801015000	2.303998000
1	-4.930685000	-2.839375000	0.737347000
6	1.429749000	0.829076000	2.355601000
7	0.217778000	-1.362214000	-3.676152000
1	0.351690000	0.549085000	-3.345587000
1	0.417498000	-1.247294000	-4.662550000
1	0.047475000	-2.286828000	-3.308460000

^{6}**4**$_{dpd}$:

26	-0.201363000	-0.187309000	-0.840089000
15	-0.524711000	2.356176000	-0.253602000
15	2.157786000	-0.892427000	0.172902000
15	-1.954589000	-1.711224000	0.439132000
7	-0.022655000	-0.066093000	-2.939540000
6	-1.146004000	-2.189369000	2.079488000
6	2.422664000	-2.685695000	0.895536000
1	1.445910000	-2.900596000	1.338956000
6	-0.291212000	-1.254400000	2.728282000
6	2.506316000	0.325370000	1.580296000
6	-1.513476000	3.255756000	-1.678436000
1	-0.793855000	3.140493000	-2.500362000
6	-1.286094000	1.256904000	2.292992000
6	1.670327000	1.812266000	3.313513000
1	0.846205000	2.216747000	3.892528000
6	-2.245718000	-3.364135000	-0.519732000
1	-2.870277000	-3.992538000	0.125069000
5	-0.065625000	0.257383000	2.326961000
6	0.358416000	-1.676976000	3.914764000
1	1.035041000	-0.994148000	4.418348000
6	0.139287000	-2.943703000	4.467098000
1	0.640361000	-3.227525000	5.386251000
6	3.800056000	0.807105000	1.828326000
1	4.640563000	0.439449000	1.257326000
6	-0.711664000	-3.842410000	3.820322000
1	-0.882451000	-4.831592000	4.231089000
6	4.034845000	1.768794000	2.821547000
1	5.045538000	2.119495000	3.000443000
6	2.967393000	2.274006000	3.565460000
1	3.136420000	3.026540000	4.328109000
6	3.488366000	-0.679037000	-1.236935000
1	3.100646000	-1.392315000	-1.975644000
6	-2.138062000	1.185108000	3.424845000
1	-1.985002000	0.391055000	4.148660000
6	-1.533848000	2.298550000	1.349731000
6	1.032872000	3.481428000	0.085889000
1	1.726969000	2.779127000	0.554983000
6	-3.722898000	-1.113248000	1.015639000
1	-3.470805000	-0.467653000	1.862563000
6	-2.573526000	3.212688000	1.571616000
1	-2.769171000	3.999527000	0.857502000
6	-3.156379000	2.118150000	3.649532000
1	-3.776114000	2.041351000	4.536299000
6	-3.003364000	-3.086757000	-1.831473000
1	-3.095382000	-4.022131000	-2.396171000
1	-2.465263000	-2.367375000	-2.460135000
1	-4.012934000	-2.706100000	-1.660600000
6	-3.375172000	3.134816000	2.718823000
1	-4.164490000	3.862658000	2.872540000
6	-0.916279000	-4.081604000	-0.794418000
1	-1.113378000	-5.024149000	-1.318974000
1	-0.366496000	-4.315987000	0.120758000
1	-0.269562000	-3.473078000	-1.436717000
6	-1.802488000	4.762712000	-1.529964000
1	-2.116815000	5.147356000	-2.508418000
1	-0.933308000	5.341333000	-1.215324000
1	-2.619596000	4.963655000	-0.832031000
6	3.408104000	0.733115000	-1.838348000
1	4.080377000	0.798642000	-2.702630000
1	3.719717000	1.494824000	-1.116206000
1	2.398217000	0.971747000	-2.181667000
6	-1.339263000	-3.468049000	2.624075000
1	-1.973553000	-4.190486000	2.123442000
6	1.658685000	3.975277000	-1.231342000
1	2.633429000	4.426131000	-1.009376000
1	1.049271000	4.741140000	-1.718962000
1	1.826706000	3.164829000	-1.946444000
6	-2.797225000	2.493823000	-2.048309000
1	-3.224911000	2.933417000	-2.957610000
1	-3.550360000	2.576509000	-1.258508000
1	-2.619090000	1.432188000	-2.246728000
6	2.682232000	-3.701830000	-0.231340000
1	2.607589000	-4.714963000	0.181538000
1	3.682489000	-3.596573000	-0.660242000
1	1.953308000	-3.622749000	-1.043106000
6	0.764325000	4.627251000	1.074400000
1	1.711997000	5.136774000	1.288568000
1	0.360641000	4.260825000	2.021754000
1	0.070869000	5.372576000	0.673807000
6	3.481379000	-2.759044000	2.007138000
1	3.504697000	-3.781382000	2.404921000
1	3.249267000	-2.084036000	2.834843000
1	4.485971000	-2.521875000	1.644222000
6	-4.436564000	-0.264534000	-0.045138000
1	-5.354624000	0.150198000	0.389048000
1	-4.724755000	-0.848542000	-0.924280000

1	-3.815920000	0.569692000	-0.375162000
6	4.950040000	-1.061276000	-0.933543000
1	5.481341000	-1.161165000	-1.888597000
1	5.053355000	-2.008484000	-0.402556000
1	5.469704000	-0.287803000	-0.361920000
6	-4.595045000	-2.272875000	1.519698000
1	-5.510525000	-1.858660000	1.959980000
1	-4.097156000	-2.862817000	2.295074000
1	-4.898196000	-2.943629000	0.708475000
6	1.407461000	0.818557000	2.339331000
7	0.257341000	-1.092073000	-3.760756000
1	-0.171922000	0.795604000	-3.466906000
1	0.296584000	-0.976295000	-4.765307000
1	0.431074000	-2.000310000	-3.358082000

^{2}**4**$_{dpd,RC}$:

26	-0.630444000	0.159577000	-1.030944000
15	-1.187969000	2.525887000	-0.201255000
15	1.741403000	-0.103905000	-0.566690000
15	-1.956968000	-1.644799000	0.395720000
7	-0.505724000	-0.661579000	-2.682401000
6	-0.838358000	-2.075641000	1.872141000
6	2.364361000	-1.876124000	-0.020957000
1	1.515523000	-2.245982000	0.560981000
6	-0.036808000	-1.048627000	2.440995000
6	2.225076000	1.040756000	0.859965000
6	-2.496406000	3.284745000	-1.442273000
1	-1.886505000	3.314413000	-2.354680000
6	-1.411288000	1.317502000	2.364744000
6	1.593450000	2.188217000	2.906854000
1	0.868194000	2.391621000	3.688836000
6	-2.242855000	-3.362673000	-0.466224000
1	-2.614822000	-4.035368000	0.314412000
5	-0.097140000	0.487960000	2.081776000
6	0.843510000	-1.402919000	3.492650000
1	1.484214000	-0.640976000	3.925469000
6	0.903723000	-2.705942000	3.998602000
1	1.579648000	-2.940880000	4.814122000
6	3.455747000	1.713879000	0.894646000
1	4.190817000	1.551122000	0.120206000
6	0.102322000	-3.700950000	3.435250000
1	0.144175000	-4.718809000	3.808632000
6	3.764191000	2.604095000	1.932635000
1	4.725455000	3.106907000	1.941358000
6	2.830330000	2.842034000	2.942637000
1	3.053652000	3.536736000	3.745497000
6	2.786692000	0.326831000	-2.152307000
1	2.418967000	-0.445734000	-2.840905000
6	-2.023242000	1.113780000	3.625034000
1	-1.642274000	0.334199000	4.277436000
6	-1.947948000	2.332710000	1.524268000
6	0.168067000	3.930540000	-0.036119000
1	1.049362000	3.357838000	0.261904000
6	-3.644084000	-1.322590000	1.343478000
1	-3.319863000	-0.644938000	2.139616000
6	-3.036579000	3.101931000	1.958284000
1	-3.448908000	3.874234000	1.324244000
6	-3.099922000	1.897019000	4.058040000
1	-3.540175000	1.722241000	5.034105000
6	-3.316109000	-3.264417000	-1.565740000
1	-3.455652000	-4.253957000	-2.019013000
1	-3.012334000	-2.577775000	-2.362262000
1	-4.285644000	-2.940890000	-1.180323000
6	-3.609081000	2.891921000	3.221239000
1	-4.447549000	3.502547000	3.539396000
6	-0.934016000	-3.930657000	-1.034033000
1	-1.124998000	-4.918359000	-1.472474000
1	-0.162074000	-4.048788000	-0.268820000
1	-0.539621000	-3.283361000	-1.822893000
6	-3.016775000	4.712153000	-1.173539000
1	-3.458089000	5.099353000	-2.101050000
1	-2.240864000	5.411839000	-0.862676000
1	-3.808029000	4.721568000	-0.418401000
6	2.369914000	1.697080000	-2.710354000
1	2.860037000	1.866529000	-3.677297000
1	2.671889000	2.507331000	-2.038886000
1	1.287701000	1.759265000	-2.861686000
6	-0.753913000	-3.385172000	2.371179000
1	-1.346769000	-4.180294000	1.935501000
6	0.466661000	4.594036000	-1.393471000
1	1.381985000	5.191593000	-1.299886000
1	-0.331779000	5.269851000	-1.711272000
1	0.631140000	3.862896000	-2.189769000
6	-3.685210000	2.342545000	-1.690084000
1	-4.295183000	2.741748000	-2.510348000
1	-4.327832000	2.276093000	-0.806725000
1	-3.379518000	1.330381000	-1.969431000
6	2.558083000	-2.799955000	-1.235391000
1	2.706492000	-3.827990000	-0.883022000
1	3.439334000	-2.528737000	-1.824573000
1	1.687731000	-2.805717000	-1.897594000
6	-0.121528000	4.962720000	1.065002000
1	0.739194000	5.638341000	1.151008000
1	-0.270605000	4.486749000	2.037424000
1	-1.001731000	5.575477000	0.847277000
6	3.598545000	-1.860065000	0.894957000
1	3.814289000	-2.887692000	1.214387000
1	3.429643000	-1.259958000	1.792318000
1	4.491356000	-1.477226000	0.390827000
6	-4.674171000	-0.582482000	0.478343000
1	-5.531118000	-0.295452000	1.101046000
1	-5.057675000	-1.201825000	-0.338869000
1	-4.252935000	0.327172000	0.048630000
6	4.322518000	0.195047000	-2.109914000
1	4.694688000	0.143119000	-3.141074000
1	4.669649000	-0.699594000	-1.590720000
1	4.797083000	1.064992000	-1.648651000
6	-4.237586000	-2.583943000	1.989128000
1	-5.089534000	-2.295717000	2.618377000
1	-3.517141000	-3.101145000	2.629973000
1	-4.611108000	-3.292701000	1.241870000
6	1.266434000	1.263611000	1.885954000
7	-1.471193000	-0.703658000	-3.564787000
1	0.363968000	-0.939130000	-3.155665000

1	-2.368673000	-0.470467000	-3.105749000
1	4.091228000	-1.818518000	-5.879452000
8	0.717529000	-1.725352000	-5.194785000
6	0.322707000	-3.157616000	-5.187892000
6	0.153600000	-3.750058000	-6.582204000
1	1.059717000	-3.717004000	-4.603256000
1	-0.620070000	-3.152966000	-4.637731000
1	-0.264795000	-4.758968000	-6.495711000
1	-0.530789000	-3.148822000	-7.188529000
1	1.105606000	-3.833121000	-7.115737000
6	1.983963000	-1.412305000	-5.902552000
6	3.184619000	-2.205278000	-5.400996000
1	1.834585000	-1.563722000	-6.977055000
1	2.126158000	-0.345159000	-5.717904000
1	3.303651000	-2.104318000	-4.318034000
1	3.113342000	-3.269675000	-5.643766000
1	-1.418339000	1.083081000	-8.784924000
8	-1.256847000	-0.273339000	-6.375570000
6	-2.771483000	-0.307262000	-6.381738000
6	-3.244947000	-1.742735000	-6.407179000
1	-3.093713000	0.242559000	-5.496596000
1	-3.040733000	0.236837000	-7.286069000
1	-2.887514000	-2.263642000	-7.298967000
1	-2.927237000	-2.286513000	-5.513615000
1	-4.340272000	-1.743317000	-6.429536000
6	-0.590197000	1.033156000	-6.775177000
6	-0.448390000	1.092767000	-8.279610000
1	-1.212313000	1.829537000	-6.361255000
1	0.367527000	1.009635000	-6.257780000
1	0.053633000	2.030277000	-8.543143000
1	0.155871000	0.262159000	-8.652806000
1	-0.807041000	-0.749521000	-5.591065000

${}^{4}\mathbf{4}_{dpd,RC}$:

26	-0.681995000	0.105111000	-1.064437000
15	-1.625762000	2.380438000	-0.140153000
15	1.879498000	-0.008906000	-0.283453000
15	-1.857850000	-1.874471000	0.238752000
7	-0.712540000	0.351612000	-3.031411000
6	-0.761340000	-2.261911000	1.734230000
6	2.661577000	-1.720993000	0.228774000
1	1.829308000	-2.207013000	0.746373000
6	-0.099287000	-1.196452000	2.405468000
6	2.062122000	1.142915000	1.211366000
6	-2.980442000	3.090887000	-1.352479000
1	-2.370186000	3.205645000	-2.257897000
6	-1.740467000	0.988560000	2.367321000
6	1.067053000	2.232017000	3.145265000
1	0.232899000	2.364011000	3.826937000
6	-1.818731000	-3.476947000	-0.848072000
1	-2.143778000	-4.301499000	-0.204205000
5	-0.311417000	0.350008000	2.163766000
6	0.772510000	-1.533879000	3.470799000
1	1.309316000	-0.743127000	3.984793000
6	0.949545000	-2.856341000	3.891609000
1	1.611571000	-3.076858000	4.722074000
6	3.211183000	1.925497000	1.397242000
1	4.051975000	1.832540000	0.724863000
6	0.284632000	-3.887501000	3.225001000
1	0.420530000	-4.919078000	3.531520000
6	3.299761000	2.841197000	2.455661000
1	4.202806000	3.428599000	2.582658000
6	2.224184000	2.996916000	3.331353000
1	2.277503000	3.710937000	4.146184000
6	2.977755000	0.665048000	-1.750736000
1	2.679621000	-0.027024000	-2.549478000
6	-2.385753000	0.622510000	3.575416000
1	-1.933662000	-0.137985000	4.204148000
6	-2.375564000	1.969659000	1.550639000
6	-0.377754000	3.854669000	0.135503000
1	0.526970000	3.332833000	0.457364000
6	-3.632929000	-1.830458000	1.058777000
1	-3.464017000	-1.160947000	1.907886000
6	-3.586414000	2.544771000	1.964206000
1	-4.080244000	3.281964000	1.347455000
6	-3.580142000	1.221635000	3.990543000
1	-4.037376000	0.926978000	4.928973000
6	-2.799099000	-3.357098000	-2.027583000
1	-2.716758000	-4.252743000	-2.654932000
1	-2.559087000	-2.492086000	-2.656096000
1	-3.839769000	-3.274287000	-1.707355000
6	-4.182823000	2.185344000	3.180774000
1	-5.113522000	2.654335000	3.481555000
6	-0.393199000	-3.750113000	-1.349220000
1	-0.383238000	-4.677807000	-1.934252000
1	0.322236000	-3.864880000	-0.530993000
1	-0.048082000	-2.939880000	-2.001866000
6	-3.612371000	4.460566000	-1.036384000
1	-4.156927000	4.797884000	-1.927722000
1	-2.880509000	5.230962000	-0.791470000
1	-4.338773000	4.408922000	-0.221066000
6	2.539954000	2.086660000	-2.142804000
1	3.066557000	2.391521000	-3.054919000
1	2.783453000	2.810497000	-1.358423000
1	1.466388000	2.158232000	-2.337334000
6	-0.555083000	-3.588607000	2.143148000
1	-1.039566000	-4.407133000	1.624067000
6	-0.075514000	4.575595000	-1.190947000
1	0.796165000	5.225945000	-1.050407000
1	-0.905728000	5.207977000	-1.517093000
1	0.159357000	3.880095000	-2.002303000
6	-4.071783000	2.048851000	-1.642701000
1	-4.702548000	2.404041000	-2.466769000
1	-4.719845000	1.894963000	-0.774260000
1	-3.653165000	1.082204000	-1.935840000
6	3.016856000	-2.557549000	-1.014440000
1	3.216436000	-3.589938000	-0.702847000
1	3.915780000	-2.189597000	-1.516257000
1	2.205268000	-2.583945000	-1.747054000
6	-0.792770000	4.827364000	1.250692000
1	0.012622000	5.557166000	1.402425000
1	-0.957790000	4.310720000	2.199852000
1	-1.701475000	5.383807000	1.003138000
6	3.834522000	-1.604464000	1.214941000

1	4.150630000	-2.613420000	1.508752000
1	3.549909000	-1.066384000	2.122902000
1	4.701236000	-1.100233000	0.777229000
6	-4.702012000	-1.203306000	0.153479000
1	-5.618913000	-1.055542000	0.737501000
1	-4.956471000	-1.843432000	-0.696273000
1	-4.390305000	-0.231274000	-0.229222000
6	4.512460000	0.594874000	-1.623301000
1	4.944703000	0.769159000	-2.616952000
1	4.875739000	-0.371686000	-1.273902000
1	4.912169000	1.369266000	-0.963336000
6	-4.065760000	-3.205786000	1.589723000
1	-5.000219000	-3.088190000	2.152515000
1	-3.327443000	-3.643335000	2.267636000
1	-4.261005000	-3.915286000	0.778314000
6	0.962079000	1.276646000	2.105385000
7	-1.667803000	0.132866000	-3.932133000
1	0.088086000	0.799425000	-3.485890000
1	-2.508180000	-0.334408000	-3.619575000
1	-0.694880000	-5.407260000	-6.790671000
8	1.081991000	-2.743629000	-5.559558000
6	2.396439000	-2.664485000	-6.216241000
6	3.532362000	-3.034390000	-5.267753000
1	2.404852000	-3.302473000	-7.109230000
1	2.483282000	-1.625053000	-6.543946000
1	4.497843000	-2.932047000	-5.777107000
1	3.534065000	-2.375793000	-4.392588000
1	3.446643000	-4.069798000	-4.920053000
6	0.480404000	-4.086262000	-5.544475000
6	-0.236607000	-4.413043000	-6.850944000
1	1.251571000	-4.834776000	-5.320518000
1	-0.223737000	-4.067033000	-4.708459000
1	-1.026817000	-3.682207000	-7.051701000
1	0.454721000	-4.415837000	-7.700798000
1	-1.977870000	0.186011000	-8.902726000
8	-0.970903000	0.949381000	-6.375251000
6	-1.243594000	2.266925000	-6.970003000
6	-1.476371000	3.266748000	-5.849189000
1	-0.375619000	2.548829000	-7.579568000
1	-2.119264000	2.196421000	-7.624784000
1	-2.345988000	2.987785000	-5.244645000
1	-0.599204000	3.332168000	-5.196863000
1	-1.663545000	4.259679000	-6.271720000
6	-0.500317000	-0.088450000	-7.313005000
6	-1.605843000	-0.595734000	-8.232534000
1	0.336495000	0.331533000	-7.885985000
1	-0.120544000	-0.888389000	-6.671759000
1	-1.219715000	-1.410628000	-8.855508000
1	-2.448181000	-0.983352000	-7.649148000
1	-1.529459000	0.434078000	-4.927441000

$^{6}\mathbf{4}_{dpd,RC}$:

26	-0.912464000	0.069359000	-0.897305000
15	-1.318976000	2.567220000	-0.174218000
15	1.664279000	-0.405901000	-0.564376000
15	-2.200637000	-1.479368000	0.839623000
7	-1.333609000	0.097864000	-2.930207000
6	-0.923780000	-1.905960000	2.172958000
6	2.246149000	-2.172050000	0.027760000
1	1.453660000	-2.447822000	0.729573000
6	0.046141000	-0.933670000	2.546242000
6	2.301381000	0.845896000	0.706925000
6	-2.786732000	3.300642000	-1.228016000
1	-2.355690000	3.222708000	-2.235180000
6	-1.190390000	1.493903000	2.485042000
6	1.867497000	2.296554000	2.609984000
1	1.207654000	2.647739000	3.396921000
6	-2.637108000	-3.162175000	-0.017389000
1	-3.073283000	-3.809149000	0.752734000
5	0.055382000	0.590947000	2.138558000
6	1.043945000	-1.324874000	3.474076000
1	1.809257000	-0.609969000	3.758093000
6	1.058540000	-2.599490000	4.050555000
1	1.827178000	-2.860269000	4.770064000
6	3.570960000	1.429628000	0.585668000
1	4.243644000	1.118749000	-0.200743000
6	0.088255000	-3.534486000	3.683239000
1	0.090100000	-4.528895000	4.116472000
6	3.996876000	2.424743000	1.477141000
1	4.986640000	2.854901000	1.367930000
6	3.142100000	2.861909000	2.490352000
1	3.456618000	3.640645000	3.176808000
6	2.527619000	-0.089421000	-2.282797000
1	1.998290000	-0.815923000	-2.912024000
6	-1.651547000	1.381215000	3.821017000
1	-1.220676000	0.620782000	4.464313000
6	-1.800358000	2.479831000	1.656602000
6	0.138242000	3.857539000	-0.315673000
1	1.013844000	3.248312000	-0.073847000
6	-3.766863000	-0.950819000	1.885226000
1	-3.406443000	-0.038591000	2.368902000
6	-2.803050000	3.309637000	2.179653000
1	-3.274373000	4.056406000	1.557256000
6	-2.635196000	2.230307000	4.339753000
1	-2.952515000	2.126238000	5.371727000
6	-3.671978000	-2.945959000	-1.138260000
1	-3.832966000	-3.896081000	-1.661497000
1	-3.317827000	-2.214744000	-1.874102000
1	-4.638418000	-2.608200000	-0.762559000
6	-3.212639000	3.197519000	3.515606000
1	-3.981774000	3.859682000	3.898347000
6	-1.367464000	-3.817520000	-0.583339000
1	-1.630815000	-4.765191000	-1.067756000
1	-0.618653000	-4.029288000	0.183460000
1	-0.904716000	-3.173611000	-1.340427000
6	-3.190146000	4.772851000	-1.014399000
1	-3.818221000	5.080177000	-1.860159000
1	-2.339490000	5.454344000	-0.974401000
1	-3.786258000	4.914792000	-0.109107000
6	2.191907000	1.319492000	-2.797651000
1	2.587837000	1.435431000	-3.813906000
1	2.642800000	2.096651000	-2.171830000
1	1.113068000	1.490177000	-2.839154000
6	-0.888717000	-3.189501000	2.739096000

1	-1.619796000	-3.936567000	2.452462000
6	0.284002000	4.364533000	-1.761980000
1	1.233013000	4.906414000	-1.852892000
1	-0.515342000	5.057283000	-2.039733000
1	0.299219000	3.549050000	-2.491025000
6	-4.016326000	2.380065000	-1.153534000
1	-4.785573000	2.745896000	-1.844257000
1	-4.448177000	2.372588000	-0.147760000
1	-3.778891000	1.349688000	-1.435374000
6	2.234127000	-3.174896000	-1.140097000
1	2.333973000	-4.190361000	-0.738253000
1	3.068083000	-3.012728000	-1.828643000
1	1.304025000	-3.137973000	-1.715306000
6	0.051085000	5.004868000	0.702872000
1	0.946714000	5.631657000	0.607660000
1	0.010959000	4.634294000	1.730392000
1	-0.820443000	5.644976000	0.536604000
6	3.582478000	-2.176025000	0.786859000
1	3.762410000	-3.185098000	1.178726000
1	3.572310000	-1.485828000	1.634318000
1	4.428904000	-1.916166000	0.143621000
6	-4.991579000	-0.600727000	1.023326000
1	-5.689599000	-0.010280000	1.629259000
1	-5.525959000	-1.498187000	0.698396000
1	-4.735885000	-0.011812000	0.140041000
6	4.036711000	-0.373652000	-2.402763000
1	4.282939000	-0.450500000	-3.468911000
1	4.341665000	-1.306933000	-1.927670000
1	4.645249000	0.436087000	-1.991062000
6	-4.122704000	-1.981044000	2.967677000
1	-4.954875000	-1.590014000	3.566519000
1	-3.289002000	-2.178102000	3.646015000
1	-4.452527000	-2.931585000	2.532815000
6	1.423648000	1.265319000	1.746503000
7	-1.439465000	-0.966740000	-3.745864000
1	-1.536968000	0.958208000	-3.441809000
1	-1.227683000	-1.874028000	-3.356531000
1	4.777622000	-0.256288000	-7.699929000
8	2.198286000	-1.547557000	-5.844561000
6	2.447170000	-2.961554000	-5.527869000
6	1.519479000	-3.897363000	-6.296554000
1	3.499992000	-3.205808000	-5.720006000
1	2.274590000	-3.042036000	-4.451233000
1	1.717449000	-4.938970000	-6.017173000
1	0.471469000	-3.673566000	-6.070500000
1	1.664548000	-3.808538000	-7.378731000
6	2.875094000	-1.061961000	-7.058033000
6	4.313908000	-0.636763000	-6.782162000
1	2.834623000	-1.831989000	-7.839368000
1	2.274481000	-0.211578000	-7.391869000
1	4.343518000	0.157334000	-6.028659000
1	4.920887000	-1.476077000	-6.424859000
1	-2.165634000	-1.527683000	-8.954765000
8	-2.137486000	-0.491243000	-6.313520000
6	-3.435394000	-0.028335000	-6.823586000
6	-4.366927000	0.201214000	-5.644765000
1	-3.273523000	0.897813000	-7.391035000
1	-3.846026000	-0.783072000	-7.503539000
1	-4.503897000	-0.720633000	-5.069776000
1	-3.976754000	0.978162000	-4.978736000
1	-5.348785000	0.525274000	-6.005917000
6	-1.060705000	-0.595109000	-7.313149000
6	-1.293269000	-1.715806000	-8.320979000
1	-0.960390000	0.377570000	-7.813187000
1	-0.160895000	-0.784669000	-6.722030000
1	-0.419854000	-1.805307000	-8.976834000
1	-1.434268000	-2.673088000	-7.808212000
1	-1.734174000	-0.851949000	-4.739868000

[2]**5:**

26	-0.183625000	-0.120285000	-1.222048000
15	-0.337908000	2.304581000	-0.910264000
15	2.102866000	-0.822879000	-0.699082000
15	-1.783249000	-1.749064000	-0.348250000
7	-0.026455000	-0.432239000	-2.700059000
6	-1.119189000	-2.228262000	1.350890000
6	2.286597000	-2.685274000	-0.140760000
1	1.295819000	-2.917565000	0.257424000
6	-0.203702000	-1.353627000	2.002879000
6	2.559309000	0.300151000	0.742966000
6	-1.345630000	3.045045000	-2.400067000
1	-0.608360000	2.937600000	-3.204807000
6	-1.180453000	1.129339000	1.562952000
6	1.837154000	1.660888000	2.619959000
1	1.054981000	2.019203000	3.281386000
6	-2.073498000	-3.347629000	-1.421703000
1	-3.031749000	-3.727934000	-1.044611000
5	0.050779000	0.151943000	1.627576000
6	0.405399000	-1.812843000	3.196495000
1	1.129178000	-1.179340000	3.698666000
6	0.065748000	-3.044943000	3.765975000
1	0.533868000	-3.360383000	4.691717000
6	3.864155000	0.784867000	0.909386000
1	4.659492000	0.475763000	0.246529000
6	-0.875508000	-3.863867000	3.137699000
1	-1.155549000	-4.815421000	3.575460000
6	4.160796000	1.680547000	1.946672000
1	5.177504000	2.035611000	2.071859000
6	3.146996000	2.120290000	2.800771000
1	3.367582000	2.825066000	3.594714000
6	3.290123000	-0.610081000	-2.227091000
1	2.860588000	-1.361262000	-2.899549000
6	-2.096296000	0.998239000	2.641262000
1	-1.979489000	0.172863000	3.334376000
6	-1.387609000	2.215567000	0.657083000
6	1.202551000	3.463245000	-0.591842000
1	1.934492000	2.779028000	-0.156893000
6	-3.557951000	-0.983539000	-0.043339000
1	-3.325219000	0.019219000	0.323124000
6	-2.439006000	3.118293000	0.861819000
1	-2.605390000	3.935247000	0.174639000
6	-3.120704000	1.924739000	2.857505000
1	-3.784315000	1.809162000	3.706938000
6	-2.217406000	-2.978229000	-2.909457000

1	-2.460105000	-3.889249000	-3.468692000
1	-1.276007000	-2.582830000	-3.305951000
1	-3.004499000	-2.249465000	-3.104338000
6	-3.290389000	2.988127000	1.968103000
1	-4.083307000	3.711672000	2.120479000
6	-0.987335000	-4.418711000	-1.252955000
1	-1.301828000	-5.314037000	-1.801870000
1	-0.810926000	-4.711896000	-0.217477000
1	-0.042940000	-4.083913000	-1.686030000
6	-1.736866000	4.532384000	-2.288695000
1	-1.987233000	4.889349000	-3.294768000
1	-0.940024000	5.170806000	-1.904751000
1	-2.625450000	4.676858000	-1.668712000
6	3.163381000	0.766839000	-2.889072000
1	3.766757000	0.771162000	-3.804428000
1	3.543622000	1.561131000	-2.239366000
1	2.132462000	0.995139000	-3.170220000
6	-1.452692000	-3.463516000	1.924012000
1	-2.163756000	-4.124377000	1.442647000
6	1.763304000	4.029079000	-1.907802000
1	2.732684000	4.493970000	-1.693076000
1	1.118369000	4.802153000	-2.333095000
1	1.928607000	3.261953000	-2.667484000
6	-2.567789000	2.175904000	-2.729933000
1	-3.063176000	2.587476000	-3.616831000
1	-3.295134000	2.175814000	-1.911848000
1	-2.288845000	1.143539000	-2.955894000
6	2.572307000	-3.605130000	-1.340560000
1	2.427076000	-4.643348000	-1.021290000
1	3.602357000	-3.513587000	-1.693519000
1	1.903982000	-3.424191000	-2.187657000
6	0.889704000	4.566234000	0.432846000
1	1.819666000	5.107922000	0.643355000
1	0.518897000	4.160932000	1.377256000
1	0.162906000	5.292484000	0.056452000
6	3.319742000	-2.854619000	0.985700000
1	3.313950000	-3.904860000	1.301021000
1	3.082465000	-2.242066000	1.858567000
1	4.335645000	-2.613023000	0.659015000
6	-4.387689000	-0.870498000	-1.330917000
1	-5.293164000	-0.295774000	-1.103225000
1	-4.708903000	-1.850929000	-1.695816000
1	-3.860944000	-0.350030000	-2.133645000
6	4.770221000	-0.961944000	-1.971178000
1	5.233335000	-1.190712000	-2.938254000
1	4.917850000	-1.828753000	-1.325497000
1	5.318344000	-0.116745000	-1.546731000
6	-4.317588000	-1.761951000	1.043181000
1	-5.280574000	-1.265312000	1.212036000
1	-3.782797000	-1.785952000	1.995137000
1	-4.530604000	-2.791661000	0.734251000
6	1.515527000	0.723627000	1.609620000

^{4}**5:**

26	-0.295892000	-0.178346000	-1.791783000
15	-0.624615000	2.288868000	-1.404648000
15	2.670329000	-0.885512000	-0.239382000
15	-2.042375000	-1.564778000	-0.558173000
7	0.959166000	-0.346316000	-0.349320000
6	-1.169046000	-2.155807000	1.022241000
6	2.678060000	-2.814973000	-0.017419000
1	1.701223000	-2.988231000	0.441188000
6	-0.109019000	-1.431714000	1.627847000
6	3.019810000	0.068057000	1.306930000
6	-1.910492000	3.062173000	-2.640797000
1	-1.370066000	2.928391000	-3.587235000
6	-0.792402000	1.165329000	1.240905000
6	1.911385000	1.325342000	3.022027000
1	1.014033000	1.727002000	3.479686000
6	-2.402410000	-3.166788000	-1.595759000
1	-3.227712000	-3.691687000	-1.099052000
5	0.439948000	0.081571000	1.079288000
6	0.476995000	-1.979165000	2.791818000
1	1.295581000	-1.454817000	3.270283000
6	0.013974000	-3.168837000	3.366246000
1	0.480577000	-3.548518000	4.269387000
6	4.275163000	0.319140000	1.873338000
1	5.187710000	-0.067887000	1.435953000
6	-1.043315000	-3.860463000	2.771561000
1	-1.406976000	-4.789603000	3.196967000
6	4.337502000	1.097809000	3.034709000
1	5.298444000	1.312843000	3.488537000
6	3.158393000	1.601814000	3.599713000
1	3.210990000	2.212976000	4.494876000
6	3.706400000	-0.423781000	-1.789504000
1	3.293616000	-1.076339000	-2.566962000
6	-1.390567000	1.135596000	2.527773000
1	-1.069740000	0.375594000	3.232097000
6	-1.281103000	2.168017000	0.355474000
6	0.914829000	3.472258000	-1.338086000
1	1.687724000	2.825727000	-0.909752000
6	-3.752907000	-0.838630000	0.055575000
1	-3.475208000	0.183611000	0.332886000
6	-2.287205000	3.066875000	0.763718000
1	-2.643355000	3.826779000	0.081787000
6	-2.388603000	2.026078000	2.931320000
1	-2.809941000	1.949894000	3.928515000
6	-2.826791000	-2.787129000	-3.029497000
1	-3.038723000	-3.704666000	-3.591690000
1	-2.017027000	-2.260764000	-3.551744000
1	-3.720204000	-2.162364000	-3.066005000
6	-2.843952000	3.002899000	2.043063000
1	-3.620205000	3.702636000	2.333512000
6	-1.162779000	-4.076265000	-1.649608000
1	-1.396167000	-4.964898000	-2.248428000

1	-0.833451000	-4.410808000	-0.663928000
1	-0.326357000	-3.560885000	-2.137138000
6	-2.247387000	4.558131000	-2.499235000
1	-2.816810000	4.863781000	-3.385997000
1	-1.361271000	5.192969000	-2.445906000
1	-2.873979000	4.767933000	-1.628359000
6	3.522065000	1.040239000	-2.196280000
1	4.136863000	1.233586000	-3.082233000
1	3.846173000	1.726669000	-1.407874000
1	2.485860000	1.259619000	-2.458171000
6	-1.631181000	-3.349361000	1.609113000
1	-2.450446000	-3.898177000	1.158320000
6	1.342390000	3.889537000	-2.756390000
1	2.313952000	4.394772000	-2.705681000
1	0.632092000	4.590045000	-3.205821000
1	1.446943000	3.034543000	-3.432977000
6	-3.181983000	2.199699000	-2.686261000
1	-3.851555000	2.581510000	-3.466124000
1	-3.723979000	2.230480000	-1.735505000
1	-2.956450000	1.155020000	-2.923225000
6	2.750119000	-3.530100000	-1.374023000
1	2.585363000	-4.600059000	-1.205016000
1	3.729446000	-3.421180000	-1.850030000
1	1.980664000	-3.186768000	-2.072062000
6	0.731184000	4.674664000	-0.397156000
1	1.677223000	5.228218000	-0.344853000
1	0.470936000	4.360277000	0.617230000
1	-0.038340000	5.367978000	-0.748778000
6	3.782053000	-3.250273000	0.959994000
1	3.677401000	-4.327644000	1.130534000
1	3.697408000	-2.749420000	1.927956000
1	4.785910000	-3.075483000	0.561208000
6	-4.797800000	-0.785616000	-1.070911000
1	-5.667838000	-0.220572000	-0.715383000
1	-5.148218000	-1.786961000	-1.341784000
1	-4.427424000	-0.289141000	-1.970702000
6	5.188053000	-0.776651000	-1.551148000
1	5.726711000	-0.624064000	-2.492502000
1	5.342776000	-1.815760000	-1.250857000
1	5.642513000	-0.121208000	-0.802606000
6	-4.321136000	-1.550998000	1.292537000
1	-5.243422000	-1.038677000	1.593655000
1	-3.633433000	-1.527899000	2.140221000
1	-4.579874000	-2.594835000	1.081244000
6	1.805495000	0.541171000	1.859530000

[6]**5:**

26	-0.530460000	-0.233417000	-1.781845000
15	-0.586245000	2.306973000	-1.440082000
15	2.533148000	-0.861312000	-0.343992000
15	-2.047235000	-1.752005000	-0.392306000
7	0.818791000	-0.361948000	-0.432226000
6	-1.107102000	-2.230260000	1.177247000
6	2.642108000	-2.751037000	0.092671000
1	1.681313000	-2.922898000	0.584776000
6	-0.120624000	-1.371412000	1.734372000
6	2.919072000	0.235102000	1.101918000
6	-1.664746000	3.274052000	-2.732455000
1	-1.016035000	3.191965000	-3.614880000
6	-0.927698000	1.148664000	1.161294000
6	1.831759000	1.524468000	2.809776000
1	0.935700000	1.892123000	3.299203000
6	-2.339563000	-3.386649000	-1.378002000
1	-2.929509000	-4.041224000	-0.726216000
5	0.330772000	0.078616000	1.045818000
6	0.485657000	-1.799296000	2.937040000
1	1.241194000	-1.170670000	3.395221000
6	0.146691000	-3.005371000	3.563394000
1	0.637535000	-3.290394000	4.488545000
6	4.185336000	0.597930000	1.576240000
1	5.095748000	0.238447000	1.111800000
6	-0.814423000	-3.839914000	2.989621000
1	-1.077789000	-4.784583000	3.453248000
6	4.263361000	1.451232000	2.683241000
1	5.232995000	1.753294000	3.063268000
6	3.088798000	1.913873000	3.292340000
1	3.153941000	2.579303000	4.147206000
6	3.500285000	-0.549384000	-1.971425000
1	3.085086000	-1.302294000	-2.650905000
6	-1.578508000	1.123955000	2.423677000
1	-1.264711000	0.385879000	3.153431000
6	-1.395447000	2.142844000	0.251648000
6	1.037879000	3.335144000	-1.155263000
1	1.733646000	2.579962000	-0.777245000
6	-3.770186000	-1.124496000	0.253997000
1	-3.469641000	-0.474166000	1.083184000
6	-2.457468000	3.003961000	0.592584000
1	-2.809722000	3.741074000	-0.115883000
6	-2.615844000	1.992810000	2.770998000
1	-3.071212000	1.924910000	3.753823000
6	-3.139120000	-3.101873000	-2.661891000
1	-3.245949000	-4.031813000	-3.232830000
1	-2.621437000	-2.375727000	-3.301488000
1	-4.143563000	-2.724064000	-2.456249000
6	-3.070684000	2.935143000	1.844580000
1	-3.884196000	3.609589000	2.089344000
6	-0.997496000	-4.063459000	-1.698713000
1	-1.184324000	-5.006647000	-2.225805000
1	-0.420477000	-4.290328000	-0.798142000
1	-0.385820000	-3.433132000	-2.356479000
6	-1.921711000	4.771734000	-2.476209000
1	-2.349941000	5.205557000	-3.388457000
1	-1.012079000	5.328911000	-2.244984000
1	-2.639943000	4.943975000	-1.670505000
6	3.247720000	0.847450000	-2.541921000
1	3.792031000	0.941950000	-3.488090000
1	3.610034000	1.629949000	-1.868308000

WILEY-VCH

1	2.189546000	1.017266000	-2.748286000
6	-1.440482000	-3.447012000	1.800864000
1	-2.182070000	-4.105709000	1.362475000
6	1.578762000	3.903869000	-2.479317000
1	2.587791000	4.298449000	-2.311882000
1	0.963233000	4.729531000	-2.847276000
1	1.643344000	3.151648000	-3.271457000
6	-2.971858000	2.521455000	-3.031766000
1	-3.464437000	2.985468000	-3.894476000
1	-3.667934000	2.569596000	-2.188708000
1	-2.795589000	1.468391000	-3.275970000
6	2.740415000	-3.621386000	-1.169213000
1	2.650915000	-4.670979000	-0.867284000
1	3.702739000	-3.509745000	-1.677709000
1	1.939414000	-3.417193000	-1.885009000
6	0.876610000	4.419355000	-0.076408000
1	1.844443000	4.914692000	0.070515000
1	0.567543000	3.995238000	0.882316000
1	0.150636000	5.185830000	-0.363075000
6	3.776751000	-3.016101000	1.096825000
1	3.732165000	-4.071288000	1.389353000
1	3.674089000	-2.413399000	2.002748000
1	4.767009000	-2.835306000	0.666962000
6	-4.499506000	-0.273480000	-0.796929000
1	-5.370776000	0.202279000	-0.330703000
1	-4.863958000	-0.874193000	-1.636326000
1	-3.857405000	0.516887000	-1.191734000
6	5.004139000	-0.820949000	-1.765950000
1	5.493923000	-0.777861000	-2.744694000
1	5.212128000	-1.804636000	-1.338347000
1	5.467549000	-0.058804000	-1.132655000
6	-4.654367000	-2.257085000	0.798367000
1	-5.562416000	-1.819325000	1.231091000
1	-4.158187000	-2.829127000	1.587253000
1	-4.968993000	-2.947665000	0.008011000
6	1.711570000	0.667243000	1.700861000

WILEY-VCH
